# Porcine anti-human lymphocyte immunoglobulin depletes the lymphocyte population to promote successful kidney transplantation

**DOI:** 10.3389/fimmu.2023.1124790

**Published:** 2023-03-09

**Authors:** Limin Zhang, Haoyong Zou, Xia Lu, Huibo Shi, Tao Xu, Shiqi Gu, Qinyu Yu, Wenqu Yin, Shi Chen, Zhi Zhang, Nianqiao Gong

**Affiliations:** ^1^ Institute of Organ Transplantation, Tongji Hospital, Tongji Medical College, Huazhong University of Science and Technology; Key Laboratory of Organ Transplantation, Ministry of Education; NHC Key Laboratory of Organ Transplantation, Key Laboratory of Organ Transplantation, Chinese Academy of Medical Sciences, Wuhan, China; ^2^ Department of Research and Development, Wuhan Institute of Biological Products, Wuhan, China; ^3^ Department of Intensive Care Unit, Wuhan Fourth Hospital, Wuhan, China

**Keywords:** porcine anti-human lymphocyte immunoglobulin, lymphocyte-depleting, kidney transplantation, induction therapy, lymphocyte

## Abstract

**Introduction:**

Porcine anti-human lymphocyte immunoglobulin (pALG) has been used in kidney transplantation, but its impacts on the lymphocyte cell pool remain unclear.

**Methods:**

We retrospectively analyzed 12 kidney transplant recipients receiving pALG, and additional recipients receiving rabbit anti-human thymocyte immunoglobulin (rATG), basiliximab, or no induction therapy as a comparison group.

**Results:**

pALG showed high binding affinity to peripheral blood mononuclear cells (PBMCs) after administration, immediately depleting blood lymphocytes; an effect that was weaker than rATG but stronger than basiliximab. Single-cell sequencing analysis showed that pALG mainly influenced T cells and innate immune cells (mononuclear phagocytes and neutrophils). By analyzing immune cell subsets, we found that pALG moderately depleted CD4^+^T cells, CD8^+^T cells, regulatory T cells, and NKT cells and mildly inhibited dendritic cells. Serum inflammatory cytokines (IL-2, IL-6) were only moderately increased compared with rATG, which might be beneficial in terms of reducing the risk of untoward immune activation. During 3 months of follow-up, we found that all recipients and transplanted kidneys survived and showed good organ function recovery; there were no cases of rejection and a low rate of complications.

**Discussion:**

In conclusion, pALG acts mainly by moderately depleting T cells and is thus a good candidate for induction therapy for kidney transplant recipients. The immunological features of pALG should be exploited for the development of individually-optimized induction therapies based on the needs of the transplant and the immune status of the patient, which is appropriate for non-high-risk recipients.

## Introduction

Kidney transplantation (KTx) is the most effective treatment for end-stage renal disease (1, 2). The development of immunosuppressant therapies has greatly improved organ and recipient survival (3–5). However, acute rejection (AR) and delayed graft function (DGF) remain treatment obstacles that impair KTx outcomes, especially in the current era of donation after circulatory death (DCD) (6, 7). Induction therapy, based on the conventional triple maintenance immunosuppressive therapy, can reduce the incidence of postoperative AR and DGF, allow for reduced immunosuppressant doses, and prolong transplanted kidney and recipient survival (8–10). To date, induction therapy has been used in the majority of recipients as a part of immunosuppressive protocol.

Induction therapy can be subdivided into two strategies involving either the use of non-lymphocyte-depleting monoclonal antibodies or lymphocyte-depleting polyclonal antibodies (11). The non-lymphocyte-depleting strategy is represented by the interleukin-2 (IL-2) receptor antagonist basiliximab, which inhibits lymphocyte proliferation (12, 13). The lymphocyte-depleting strategy is represented by the rabbit anti-human thymocyte immunoglobulin (rATG). In the current era of DCD, given the weaker immunosuppressive potency of non-depleting treatment compared to lymphocyte-depleting drugs, basiliximab use is limited to recipients at high risk of AR or DGF (14). In addition, while the depleting rATG effectively inhibits the incidence of AR, it also increases the risks of corresponding complications such as lymphopenia and infection (15, 16); however, little is known about its effects on the lymphocyte populations (17, 18).

In addition to rATG, the porcine anti-human lymphocyte immunoglobulin (pALG) is also a lymphocyte-depleting agent. pALG is produced by immunizing pigs with human-derived thymocytes. Unlike rATG which is made in rabbits, pALG origins are from a genetically closer species to humans and therefore improved antigenic similarity, which may result in weaker cytolytic capacity on human lymphocytes than rATG (19). pALG has been successfully used as first-line treatment in acquired severe aplastic anemia (20–22). It has also been employed for other hematological indications such as graft versus host disease prophylaxis (23). Meanwhile, pALG has been used in transplantation including hematopoietic stem cell transplantation (19, 24, 25) and kidney transplantation (26). Pharmacokinetic studies show that, in humans, pALG reaches peak concentration immediately after administration and then markedly decreases over 2-3 months to undetectable levels (27). However, its immediate effects on the lymphocyte population remain unknown; such effects can directly influence the clinical course by affecting the propensity for AR, kidney function, inducing a cytokine storm, or inducing immunocompromise and facilitating the development of secondary infections during its period of physiological activity.

In this study, we retrospectively assessed a cohort of kidney recipients with organs from DCD donors who received induction therapy using pALG. The binding capability and the modulatory impact of pALG on peripheral blood mononuclear cells (PBMCs) were analyzed, and the outcomes were tracked. Our work provides important data to optimize immunosuppressive protocols by considering individual patient variables in treatment planning.

## Materials and methods

### Study cohort

This retrospective study was designed to elucidate the impact of pALG on lymphocyte populations in kidney transplant recipients. The inclusion criteria were as follows: over 18 years of age; first-time KTx; negative PRA before transplantation; using a triple immunosuppressive regime as tacrolimus (Tac)+ mycophenolic acid (MPA)+prednisone. We excluded individuals with multiple organ transplantation; previous organ transplantation; or dual kidney transplantation. From March 2022 to September 2022, 12 KTx recipients with DCD kidneys were administered pALG as the induction therapy and enrolled in this study. The demographic characteristics of these recipients are shown in **Table 1**. We also recruited six additional recipients with DCD kidneys who received rATG (n=3) or basiliximab (n=3), and three additional recipients with live donor kidneys who did not receive any induction therapy (untreated). These nine additional recipients had the same demographic characteristics as the pALG group. This study adheres to the Declaration of Helsinki and the Declaration of Istanbul, and was been approved by the Medical Ethics Committee of Tongji Hospital, Tongji Medical College, Huazhong University of Science and Technology (No. TJ-IRB20221216).

**Table 1:** Demographic characteristics of donors and recipients.

Characteristic	pALGn=12	rATGn=3	Basiliximabn=3	Untreatedn=3	*P* value
Donors					
Male gender, *n* (%)	10 (83.3)	1 (33.3)	3 (100.0)	2 (66.7)	0.265
Mean age, yrs (SD)	53.3 (10.7)	55.7 (14.8)	52.3 (8.1)	50.0 (16.7)	0.947
Mean BMI, kg/m^2^ (SD)	24.2 (4.1)	20.9 (1.9)	24.6 (6.2)	22.7 (3.0)	0.613
Mean HLA mismatch, n (SD)	4.7 (0.7)	3.7 (1.2)	4.0 (1.0)	3.3 (0.6)	0.053
Donation classification, n (%)					
Deceased donor	12 (100.0)	3 (100.0)	3 (100.0)	0 (0.0)	
Living donor	0 (0.0)	0 (0.0)	0 (0.0)	3 (100.0)	
Mean creatinine before donation, μmol/L (SD)	87.1 (48.5)	27.0 (7.0)	73.3 (19.8)	75.7 (8.0)	0.181
Mean eGFR before donation, mL/min/1.73m^2^ (SD)	95.7 (27.5)	123.2 (3.4)	103.1 (18.4)	95.5 (12.6)	0.352
Mean warm ischemia time, min (SD)	20.6 (2.3)	17.5 (2.1)	19.0 (2.8)	1.7 (0.3)	
Mean cold ischemia time, h (SD)	9.0 (3.3)	8.5 (0.5)	10.7 (2.7)	1.6 (0.2)	
Recipients					
Male gender, *n* (%)	9 (75.0)	1 (33.3)	2 (66.7)	2 (66.7)	0.701
Mean age, yrs (SD)	37.2 (7.1)	37.7 (5.1)	37.9 (7.0)	34.3 (9.5)	0.310
Mean BMI, kg/m^2^ (SD)	20.1 (2.6)	21.7 (2.9)	21.3 (2.7)	23.0 (1.9)	0.112
Dialysis type, *n* (%)					0.686
HD	11 (91.7)	3 (100.0)	2 (66.7)	3 (100.0)	
PD	1 (8.3)	0 (0.0)	1 (33.3)	0 (0.0)	
Mean duration of dialysis, months (SD)	44.5 (30.2)	25.0 (15.4)	28.7 (5.7)	8.7 (5.8)	0.167

yrs, years; BMI, body mass index; SD, standard deviation; h, hours; eGFR, estimated glomerular filtration rate; HD, hemodialysis; PD, peritoneal dialysis; min, minutes.

### Immunosuppressive therapy

**Figure 1:**
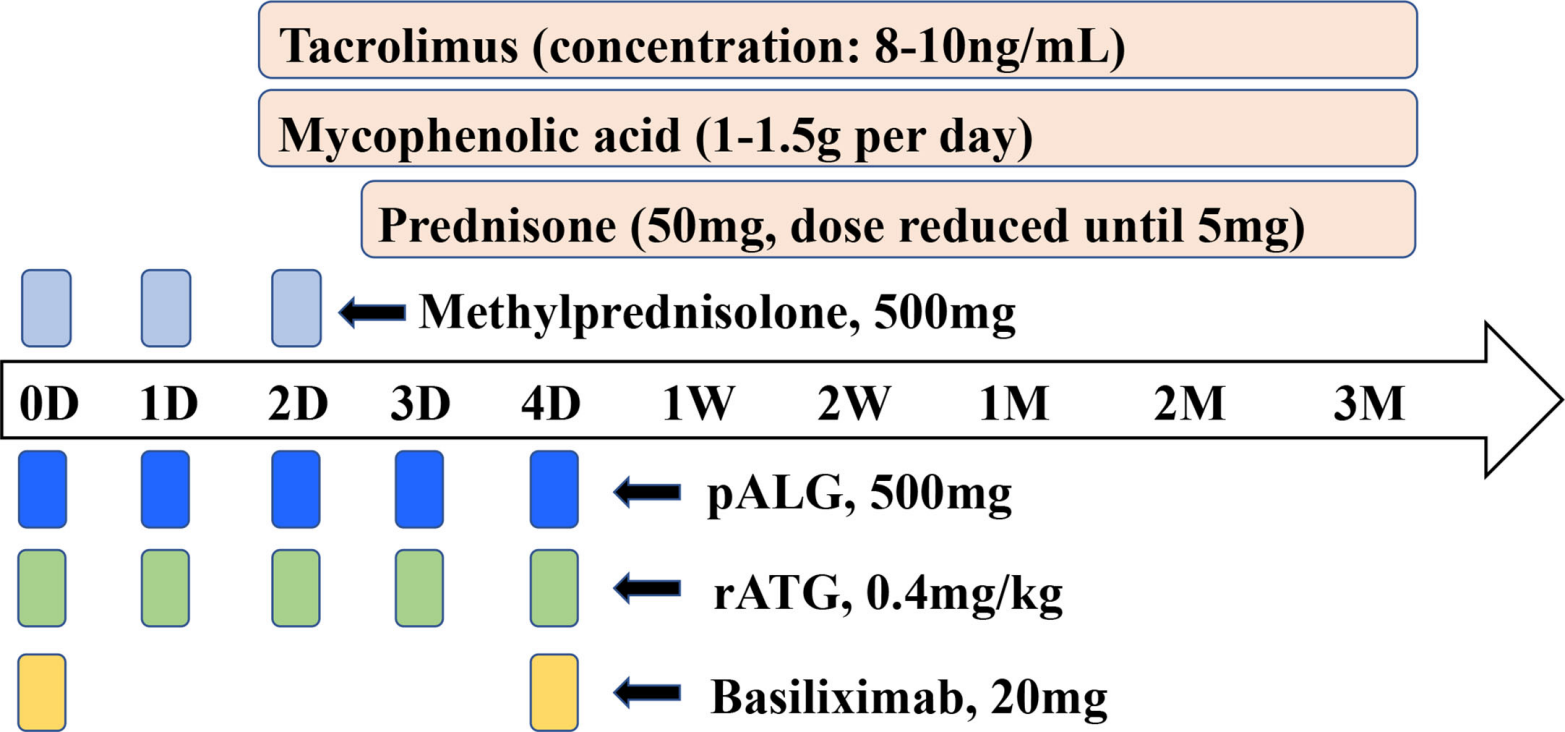
Induction therapy and immunosuppressive protocol for kidney transplantation. Induction therapy was provided using pALG, rATG, basiliximab or untreated. Methylprednisolone was administrated at a daily dose of 500mg to all recipients from POD0 to POD2. Initial and maintenance immunosuppression therapy was tacrolimus + mycophenolic acid+ prednisone. The dosage and usage were shown. D, day. W, week. M, month.

The agents used for induction therapy included pALG, rATG or basiliximab. The initiation and maintenance immunosuppressive regimes were administered as Tac+ MPA+ prednisone. The detailed dosages and drug regimens are shown in **Figure 1**.

### Recipient management

Postoperatively, recipients were monitored daily for the first week and every 2 days for the second week. All recipients were followed up for 3 months. Preoperative data on postoperative day (POD) 3, 5, 7, 14, 30, 60 and 90 were documented, including red blood cell (RBC), white blood cell (WBC), neutrophil, lymphocyte, serum creatinine (sCr), and the estimated glomerular filtration rate (eGFR). Meanwhile, within one week of transplantation, peripheral blood mononuclear cells (PBMCs) were isolated for further analysis. Clinical sequela including DGF and AR was recorded. DGF was defined as the requirement of hemodialysis in the first week after transplantation (28). AR was diagnosed based on the Banff 2019 criteria (29).

### pALG quantification and binding affinity

In the recipients who received pALG, we tested the serum concentration of pALG and the pALG binding affinity to PBMCs based on fluorescence signal intensity preoperatively and on POD 3, 5, and 7. To determine the serum pALG concentrations, ELISA plates were coated with rabbit anti-swine IgG (Fab’2) (5 μg/mL, Sigma, Billerica MA, USA) overnight at 4°C. The plates were blocked with 10% fetal calf serum (Gibco, Thornton, Australia)/phosphate buffered solution (PBS) (HyClone, Utah, USA) for 2-3 hours at room temperature. 50 μL of plasma samples were diluted with 1% fetal calf serum/PBS and added to each well and incubated at room temperature for 1 hour. Series diluted pALG were used as standards. Plates were washed 5 times with PBS containing 0.05% Tween-20 (Sigma, Louis, MO, USA). 100 μL of diluted goat anti-porcine IgG-HRP antibody (Southern Biotech, Birmingham, AL, USA) was added and incubated for 1 hour. Plates were washed 7 times followed by the addition of 100 μL Tetramethylbenzidine Substrate Solution (Sigma, Louis, MO, USA) and incubated until color development was complete. 100 μL 2N H_2_SO_4_ stopping solution was then added. The optical density was measured at 450 nm with a microtiter plate reader.

PBMCs were isolated from 5ml fresh anticoagulant whole blood by density gradient centrifugation using Ficoll-Paque Plus medium (GE Healthcare, Uppsala, Sweden) and washed with Ca/Mg-free phosphate buffered solution (PBS) (HyClone, Utah, USA). To remove the red blood cells, 2 mL GEXSCOPE^®^ red blood cell lysis buffer (Singleron, Nanjing, China) was added at 25°C for 10 minutes. The solution was then centrifuged at 500g for 5 min and suspended in PBS. The blood samples were centrifuged at 400g for 5 min at 4°C, and the supernatant was discarded. After removing red blood cells, PBMCs were isolated by centrifugation at 400g for 10 min at 4°C. The supernatant was discarded and the PBMCs were resuspended by PBS to obtain a single-cell suspension. After that, PBMCs were incubated with fluorescent-conjugated goat anti-porcine IgG (Southern Biotech, Birmingham, AL, USA), and then detected by flow cytometry. To determine the pALG binding affinity in serum, PBMCs were obtained from normal human peripheral blood by the method described above. The isolated PBMCs were diluted to 2×10^6^ cells/mL. A pALG gradient was diluted over at least 7 concentrations to generate a set of standards. 100 μL PBMC suspension was placed in a centrifuge tube and 10 μL pALG solutions of different concentrations or 10 μL recipient serum were added. After incubation at 4°C for 1h, the PBMC suspension was centrifuged and the supernatant was removed. Then 100 μL of diluted fluorescent-conjugated goat anti-porcine antibody (Southern Biotech, Birmingham, AL, USA) was added to each tube. After incubation in dark at 4°C for 30 min, the binding affinity was detected by flow cytometry.

### Single-cell sequencing analysis

PBMCs from the pALG group were isolated by the previously described methods (30). Single-cell suspensions (2×10^5^ cells/mL) with PBS (HyClone, Utah, USA) were loaded onto microwell chip using the Singleron Matrix^®^ Single Cell Processing System. Barcoding Beads were then collected from the microwell chip, followed by reverse transcription of the mRNA captured by the Barcoding Beads to obtain cDNA, and conduct PCR amplification. The amplified cDNA was then fragmented and ligated with sequencing adapters. The scRNA-seq libraries were constructed according to the protocol of the GEXSCOPE^®^ Single Cell RNA Library Kits (Singleron, Hangzhou, China) (31). Individual libraries were diluted to 4 nM, pooled, and sequenced on Illumina novaseq 6000 with 150 bp paired end reads. Scanpy v1.8.2 was used for quality control, dimensionality reduction and clustering under Python 3.7 (32). For each sample dataset, after filtering expression matrix, 15,243 cells were retained for the downstream analyses. The raw count matrix was normalized by total counts per cell and logarithmically transformed into normalized data matrix. Principle component analysis was performed on the scaled variable gene matrix, and top 20 principle components were used for clustering and dimensional reduction. Cell clusters were visualized by using Uniform Manifold Approximation and Projection (UMAP). To identify differentially expressed genes (DEGs), we used the Seurat FindMarkers function based on Wilcox likelihood-ratio test with default parameters, and selected the genes expressed in more than 10% of the cells in a cluster and with an average log (Fold Change) value greater than 0.25 as DEGs. The cell type identity of each cluster was determined with the expression of canonical markers found in the DEGs using the SynEcoSys database. The cluster identification criteria involved two steps. The first was classification and the second was annotation of cell type. First, unsupervised clustering was carried out. According to the similarity of the transcriptome, corresponding expression patterns were gathered together for clustering. Then the cells were annotated according to the up-regulated marker genes in each cell group. The total number of cells and the number of subjects were shown in **Supplementary Table 1**. Heatmaps were generated by Seurat v3.1.2 DoHeatmap. The genes in the Heatmap were all DEGs, and the top 3 genes (ranked by AVG_logFC value) were then selected and shown in the plot. Mean expression in groups of heatmaps was the average expression of the gene in the subpopulation.

### Immune cell subset profiling and cytokine quantification

Recipient whole blood was collected preoperatively and on POD 3, 5, and 7. Erythrocytes were lysed. The resulting cell suspension was incubated with Pacific Blue-conjugated anti-CD3, PE-Cy5-conjugated anti-CD4, FITC-conjugated anti-CD8, PE-conjugated anti-CD16, APC-conjugated anti-CD19, PE-conjugated anti-CD25, APC-Cy7-conjugated anti-CD127, and PE-conjugated anti-CD56 antibodies (4A Biotech, Beijing, China) in dark at 4°C for 20 minutes. We gated mononuclear cells with PE-Cy7-conjugated anti-CD45 antibodies after excluding cell debris or non-single cells on the basis of FSC/SSC plots. In parallel, PBMCs were thawed and washed in PBS once and incubated with PE-conjugated anti-CD11c, FITC-conjugated anti-CD68, and FITC-conjugated anti-HLA-DR antibodies (4A Biotech, Beijing, China) in dark at 4°C for 20 minutes. The catalog numbers of the antibodies were shown in **Supplementary Table 2**. Human Fc receptor blocking solution (4A Biotech, Beijing, China) was used as an Fc block. The proportions of DC (CD11C+, HLA-DR+), CD4^+^T cell (CD3+, CD4+), CD8^+^T cell (CD3+, CD8+), Treg (CD3+, CD4+, CD25+, CD127-), B cell (CD3-, CD19+), macrophage (Mϕ, CD68+), NK cell (CD3-, CD16+, CD56+), NKT cell (CD3+, CD16+, CD56+) were detected by flow cytometry.

Serum cytokine proteins were detected by using the Bioagent LEGENDplex TM test kit (Biolegend, San Diego, CA, USA), with IL-2 and IL-6 as targets.

### Statistics

Count data are summarized as proportions (%). Measurement data are summarized as mean ± standard deviation or mean. Fisher’s exact test was used to test differences between categorical variables. Univariate general linear models or Kruskal-Wallis H tests were used to compare differences according to the results of normal distribution and homogeneity of variance. All statistical analyses were performed by using IBM SPSS Statistics 24.0 (IBM, Armonk, NY, USA). P<0.05 indicates a statistically significant difference.

## Results

### Demographics

The demographic characteristics of all recipients and their corresponding donors are shown in **Table 1**. The donor gender, age, BMI, human leukocyte antigen mismatch, sCr, and eGFR before the donation were similar between groups. The recipient gender, age, BMI, type of dialysis and duration of dialysis were also comparable.

### pALG has a high binding affinity to PBMCs

**Figure 2:**
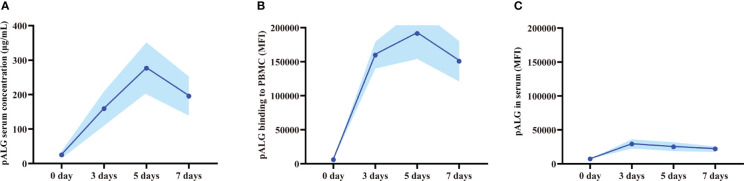
Serum pALG concentration and its binding affinity to PBMCs within 7 days after kidney transplantation. **(A)** The serum concentration of pALG. **(B)** The affinity of pALG binding to recipient PBMCs. **(C)** Serum pALG binding affinity to PBMCs from healthy volunteer. Data are expressed as mean (solid line) and SD (shaded area). PBMC, peripheral blood mononuclear cell.

After drug administration, we tested the serum concentration of pALG. A cumulative effect was seen during the 5-dose injection cycle with the concentration of up to 277 μg/mL at day 5 (**Figure 2A**). Thereafter, the serum level decreased gradually. As a lymphocyte-depleting antibody, the affinity of the bound pALG on PBMCs was examined by its flow cytometry MFI values within one week after drug administration. On day 5, the MFI values peaked at 191,457 (**Figure 2B**), suggesting that pALG has a high affinity to its target cells. After incubating PBMCs derived from healthy volunteers with serum, the MFI value showed a peak of only 29,193 at day 3 and then decreased (**Figure 2C**). These findings indicate that most of the administered pALG quickly binds to its target PBMCs to exert its effects.

### pALG depletes blood lymphocytes

**Figure 3:**
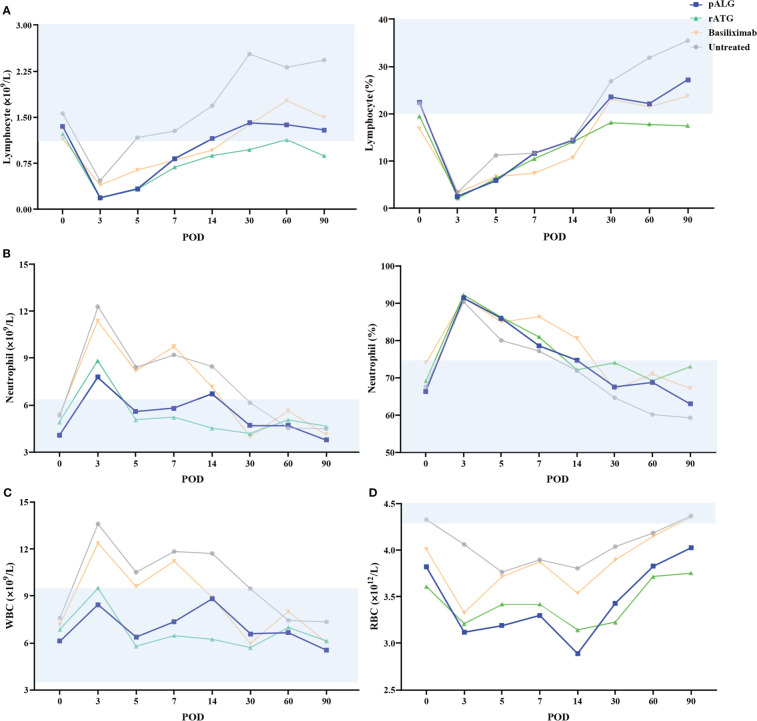
Changes in routine blood tests during the 3 months of follow-up. **(A)** The number and proportion of lymphocytes. **(B)** The number and proportion of neutrophils. **(C)** The number of WBC. **(D)** The number of RBC. Shaded area represents the normal range. WBC, white blood cell. RBC, red blood cell. POD, postoperative day.

After binding to its target cells, pALG directly depletes them which should be reflected by changes in blood cell counts. Routine blood tests showed that the number and proportion of lymphocytes decreased immediately and markedly after pALG administration; this effect was maximal on POD3 at 0.19◊10^9^/L and 2.6%, respectively. Afterward, the number and proportion of lymphocytes gradually increased reaching a clinically normal range within 3 months (**Figure 3A**). These trends indicate that pALG effectively and temporarily depletes lymphocytes. Interestingly, the decrease and recovery of lymphocytes were faster in recipients who received pALG compared to those who received rATG, but slower than those receiving basiliximab.

We also evaluated neutrophils and whole WBCs. The number and proportion of neutrophils increased significantly from POD0 to POD3 and then decrease gradually (**Figure 3B**). These changes likely stem from the high-dose methylprednisolone and stress responses to the transplant. WBCs showed similar trends as neutrophils (**Figure 3C**). We also tested RBCs but found no relationship with pALG administration (**Figure 3D**).

Therefore, pALG treatment resulted in robust but moderate lymphocyte depletion, with relatively fast recovery to normal levels after the cessation of treatment.

### pALG effects on immune cell composition

**Figure 4:**
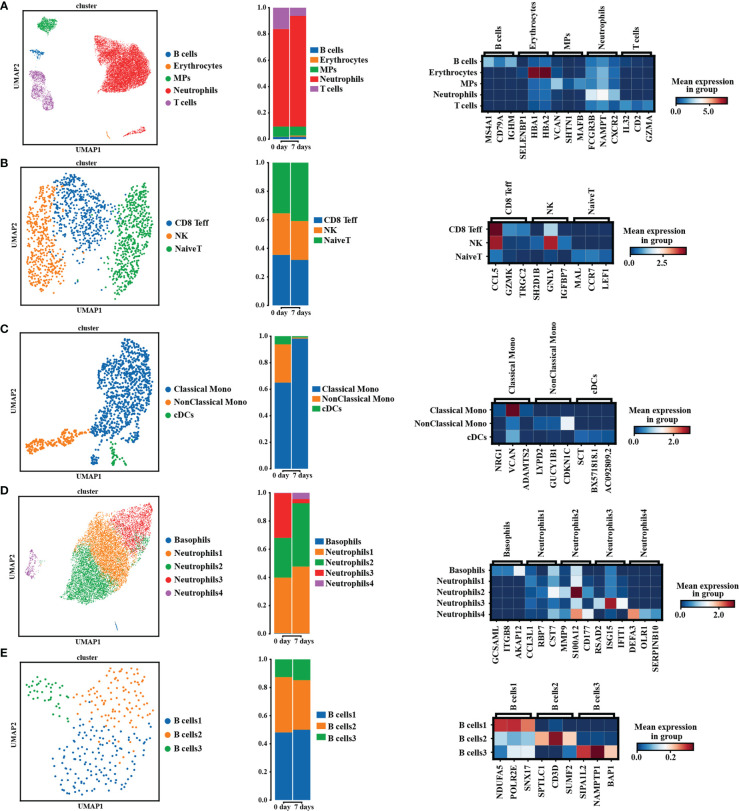
Single-cell sequencing analysis. The analysis of PBMCs’ composition in pALG group on POD0 and POD7. [**(A–E)**, left] UMAP cluster cell analysis of PBMCs, T cells, MPs, neutrophils and B cells. Each dot corresponds to a single cell. [**(A–E)**, middle] Percentage of different cell types. [**(A–E)**, right] Heatmaps shows the 3 genes with the highest expression levels in each subcluster, with columns representing selected marker genes. UMAP, uniform manifold approximation and projection. MPs, mononuclear phagocytes. CD8 Teff, CD8 effector T cells. Mono, monocytes. cDCs, conventional DCs.

To more precisely determine the effects of pALG on the immune cell pool, we performed single-cell sequencing on PBMCs obtained from one recipient preoperatively and on POD7. In the cell cluster analysis, we identified 5 cell types (**Figure 4A**, left) including B cells, erythrocytes, mononuclear phagocytes (MPs), neutrophils and T cells. The bar diagram shows the percentage of each cluster (**Figure 4A**, middle. Neutrophils, T cells and MPs were the main cell populations. Compared to the preoperative state, at POD7 neutrophils were more abundant while T cells and MPs were well less abundant. We show the highly expressed gene markers of each cell cluster in **Figure 4A**, right. After that, we analyzed the compositions of T cell subsets (**Figure 4B**), MPs (**Figure 4C**), neutrophil subsets (**Figure 4D**) and B cells (**Figure 4E**); this included cell clustering analysis (left), percentage of different cell clustering (middle) and the marker genes expressed in different cell subsets (right). We identified pALG-associated trends among the different cell types. At POD7, pALG treatment increased naïve T cells and reduced CD8 effector T cells (CD8 Teff), reduced non-classical monocytes and cDCs but increased classical monocytes, and increased neutrophils 1, 2, and 4 but reduced neutrophils 3. The neutrophils identified in this study should be the low-density neutrophils which could be co-segregated with PBMCs as previous study reported (33). There were few B cells and we found little difference after pALG. These findings show that pALG most affects the T cell and innate immune cell (MPs and neutrophils) populations.

### pALG modulates the component of lymphocyte repertoire

**Figure 5:**
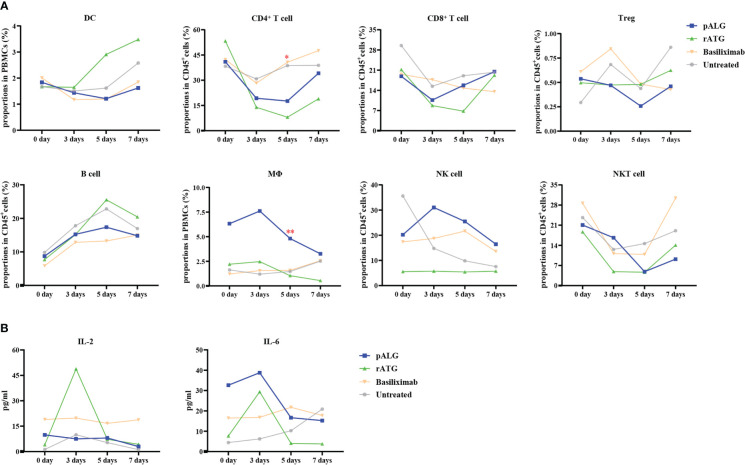
Recipients’ immune cell populations after kidney transplantation. **(A)** Proportions of DC, CD4^+^T cell, CD8^+^T cell, Treg, B cell, Mϕ, NK cell and NKT cell in PBMCs or CD45^+^T cells at 0 day, 3 days, 5 days and 7 days. Mϕ, macrophage. **(B)** Serum levels of IL-2 and IL-6. (*p<0.05, **p<0.01).

The change in immune cell subpopulations after transplantation reflects the effect of pALG on the immune system, as shown in **Figure 5A**. As antigen-presenting cells, DCs showed a mild decrease in abundance within 5 days and recovery at POD7. CD4^+^T cells, CD8^+^T cells, Tregs, and NKT cells all decreased significantly to their lowest level on POD3 - 5 and then gradually recovered, indicating that pALG robustly depletes T cells. On the contrary, we observed a small increase in B cells. Mϕ and NK cells increased on POD3 and then began to decrease; this may reflect acute immune responses to surgery. The potency of pALG on immune cell depletion was between that of rATG and basiliximab/untreated control. Furthermore, the proportion of CD4^+^T cells and Mϕ on POD5 among different groups had statistically significant differences. These findings suggest that pALG is a mild depletor of antigen-presenting cells, moderate depletor of T lymphocytes, and does not deplete B cells.

After administration, lymphocyte-depleting antibody immediately depletes lymphocyte, thereby liberating intracellular cytokines, thus inducing a cytokine storm. Compared with rATG administration, pALG administration resulted in lower IL-2 levels. pALG administration increased IL-6 to higher levels than seen after rATG administration, but given the baseline differences the fold increase after pALG administration was much lower than after rATG administration, and IL-6 decreased quickly thereafter (**Figure 5B**). Although we compared the trend of IL-2 and IL-6 levels between pALG and rATG group, there were no statistical differences. These findings indicate that pALG induces moderate lymphocytolysis compared to rATG, and therefore may reduce the risk of a cytokine storm.

### pALG results in a low rejection risk

We observed no AR after pALG administration, other induction therapies, or in the no therapy controls during the 3 months of follow-up.

### Renal function and graft and recipient survival

**Figure 6:**
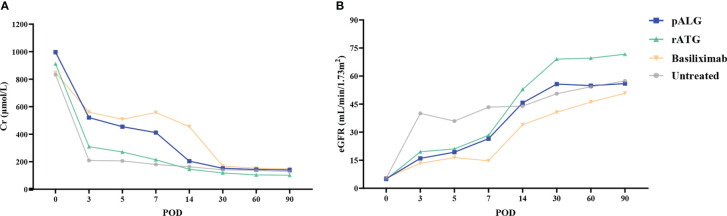
Kidney function during the 3 months after kidney transplantation. **(A)** Serum Cr level. **(B)** eGFR. Cr, creatinine. eGFR, estimated glomerular filtration rate.

Renal function was evaluated by sCr levels and eGFR. In recipients who received pALG, sCr gradually decreased to approximately 200 μmol/L post-transplantation, and was maintained around 200 μmol/L at 3 months after KTx (**Figure 6A**); eGFR increased gradually and was maintained at around 50 mL/min/1.73m^2^ during the follow-up (**Figure 6B**). Recovery of renal function in the recipients receiving pALG therapy was similar to those receiving rATG or basiliximab, and no statistical differences were observed. At 3 months post-transplantation, eGFR in the rATG group showed a small advantage over the other groups. All the kidney grafts and recipients survived during the observation period.

### Complications

All recorded complications are shown in **Table 2**. One recipient receiving pALG underwent DGF and recovered at POD10. There were three cases of infection; one pALG case developed a mild pulmonary infection with no specific identified pathogen and was cured after 5 days of treatment with cefoperazone sodium and sulbactam sodium, one pALG case was infected with JC viruria and virus cleared 1 month after the Tac was decreased, and one basiliximab case developed *Pneumocystis jiroveci* pneumonia which was cured by using caspofungin and a reduced Tac dose. Two donor-derived infections (DDI) were identified; *Candida parapsilosis* in one pALG case and *Candida glabrata* in one rATG case, both of which were cured by appropriate antibiotics following sensitivity testing.

**Table 2:** Complications after kidney transplantation.

Complication	pALG n=12	rATGn=3	Basiliximab n=3	Untreated n=3
DGF, n	1	0	0	0
Infections, n	0	0	0	0
Pneumonia	1	0	1	0
JC viruria	1	0	0	0
DDI, n	1	1	0	0

DGF, delayed graft function; DDI, donor derived infection.

## Discussion

Induction therapy is integrated into post-transplant immunosuppression protocols to modulate immunological reactivity and to protect the donor organ (16). In recipients who are at low risk for rejection, non-lymphocyte-depleting antibodies are used most often, while high-risk recipients and those with low-quality donor organs usually receive lymphocyte-depleting antibodies. Precisely targeted treatments are critical for successful transplantation (8, 11). In the current DCD era, donor organs carry greater immunogenicity potential and are of lower quality; as such, lymphocyte-depleting antibody treatment has been extended to low-risk DCD recipients at relatively lower doses (34–36). The optimal balance between treatment benefits (immunological and kidney protective) and risks (infections) of the typical lymphocyte-depleting antibody rATG still remains to be properly elucidated. pALG has been already used in the hematology and transplantation fields (20, 23), and offers an appropriate option to build an individualized induction therapy based on relevant immunological characteristics.

The first step toward elucidating the immunological characteristics of pALG is to determine its binding affinity to the target cells. In this study, we isolated PBMCs from 12 recipients who received pALG and used flow cytometry to test the binding affinity within one week of administration. The mean MFI values reached 191,457 at POD5. We also incubated serum pALG with PBMCs derived from healthy volunteers to examine its affinity potency, finding only a low MFI of 29,193 on POD3. We conclude that pALG has a high binding affinity to PBMCs and most pALG binds to its target PBMCs immediately after administration. This finding also indicates that continuous monitoring of pALG after the therapy is ceased imparts no clinical value.

The bound pALG should deplete the target cells, which can be measured directly by counting peripheral blood cell populations. To elucidate the lymphocyte depletion effects of pALG, matched recipients who received rATG, basiliximab, or no induction were also tested. We found that lymphocytes were markedly decreased immediately after pALG administration, while basiliximab exhibited only mild effects on lymphocyte numbers. Moreover, pALG showed a similar ability to deplete lymphocytes as rATG, but allowed a faster recovery after treatment cessation. Neutrophils, WBCs, and RBCs were also changed after transplantation, but this was most likely due to methylprednisolone and/or surgery-related factors rather than the induction therapy.

PBMCs and lymphocytes are complex cells with myriad subtypes. To further characterize the effect of pALG impact on immune cells, we conducted longitudinal single-cell sequencing of PBMCs that were derived from one pALG recipient. We found that T cells and innate immune cells (MPs and neutrophils) were most affected by pALG administration. PBMCs were then analyzed by flow cytometry. DCs showed a small decrease within 5 days and recovered by POD7, indicating that pALG may inhibit the initiation of immune responses by suppressing DC antigen presentation. T cells, including CD4^+^T cells, CD8^+^T cells, Tregs, and even NKT cells all decreased significantly to their lowest level 3-5 days after pALG administration and then recovered gradually. We also found a modest increase in B cells, which indicates that pALG does not deplete this population. Mϕ and NK cells were increased on POD3 and then began to decline. Because pALG significantly depleted lymphocytes, the absolute number and proportion of neutrophils were increased within three days of surgery, which was followed by a slow decline after the cessation of pALG. Furthermore, Hassani M et al. found that low-density neutrophils were co-segregated with PBMCs from whole blood by Ficoll separation (33). Therefore, the neutrophils identified by single-cell sequencing analysis should be the low-density neutrophils. And the proportion of neutrophils in this study was consistent with previous study that the proportion of neutrophils in PBMCs of patients after kidney transplantation could reach 70% or even higher through the single-cell sequencing analysis (37). These differences in innate immune cells may reflect the complex post-operative immune changes that occur in response to the surgery, inflammation, and pALG administration. Taken together, pALG modulates the lymphocyte repertoire mainly by depleting T cells, which gradually recovers after drug cessation.

The use of lymphocyte-depleting antibodies can trigger hyperactive immune responses, which are sometimes termed “the cytokine storm”, due to strong lymphocytolysis. In this work, we used IL-2 and IL-6 as a proxy measure of lymphocytolysis and the development of a cytokine storm (38–40). pALG administration led to lower IL-2 (absolute) and IL-6 (relative) compared with rATG administration. However, because our groups showed markedly different baseline values of these cytokines, larger studies are needed to verify this potential safety advantage of pALG over rATG.

Knowing that pALG drops to undetectable levels within 2-3 months (27), we conducted our observation over 3 months. Within the first week of administration, we tested the concentration kinetics, binding affinity, and lymphocyte populations. We also closely followed the clinical recovery of the cohort. Over the three months, we did not identify any cases of organ rejection and found a low rate of complications. sCr levels were found to gradually decrease and were maintained at < 200 μmol/L, and eGFR increased and maintained at approximately 50 mL/min/1.73m^2^. Based on previous experience with pALG, we administered it in five daily doses of 500mg each, from POD 0 to POD 4. Our immunological analyses and clinical outcome measures demonstrate that this protocol successfully balances treatment efficacy and patient safety. Our data suggest that our induction therapy strategy was appropriate. Although we aimed to elucidate the acute effects of pALG administration on immune cells, longer-term investigations are necessary and ongoing. In addition, due to the small sample size, there were no statistical differences among those groups for most of the parameters. Therefore, we will further expand the sample size in future studies to compare the effects between pALG and rATG or basiliximab.

In conclusion, pALG modulates lymphocytes mainly by a moderate T cell depletion effect. pALG can be used as induction therapy for kidney transplant recipients with high efficacy and safety. These immunological features of pALG treatment should inform the development of optimal and individualized induction therapies for patients according to their transplant and immune status needs, especially for non-high-risk recipients. The current cohort study should be followed-up by large sample, multi-center prospective studies.

## Data availability statement

The data of single-cell sequencing presented in the study are deposited in the Gene Expression Omnibus repository, accession number GSE226328.

## Ethics statement

The studies involving human participants were reviewed and approved by Medical Ethics Committee of Tongji Hospital, Tongji Medical College, Huazhong University of Science and Technology. The patients/participants provided their written informed consent to participate in this study.

## Author contributions

NG and ZZ: study design and manuscript reviewing. LZ, HZ, HS, TX, SG and SC: literature research and manuscript drafting. LZ, HZ, XL, HS, SG, SC, ZZ and NG: clinical studies conduct and data collection. LZ, HZ, XL, TX, QY and WY: data collection, interpretation and statistical analysis. All authors contributed to the article and approved the submitted version.
