# Supplementary figures and images for: Porcine anti-human lymphocyte immunoglobulin depletes the lymphocyte population to promote successful kidney transplantation (part 2 of 2)
Source: Front Immunol. 2023 Mar 9;14:1124790. doi: 10.3389/fimmu.2023.1124790 (PMC10033525; doi:10.3389/fimmu.2023.1124790)

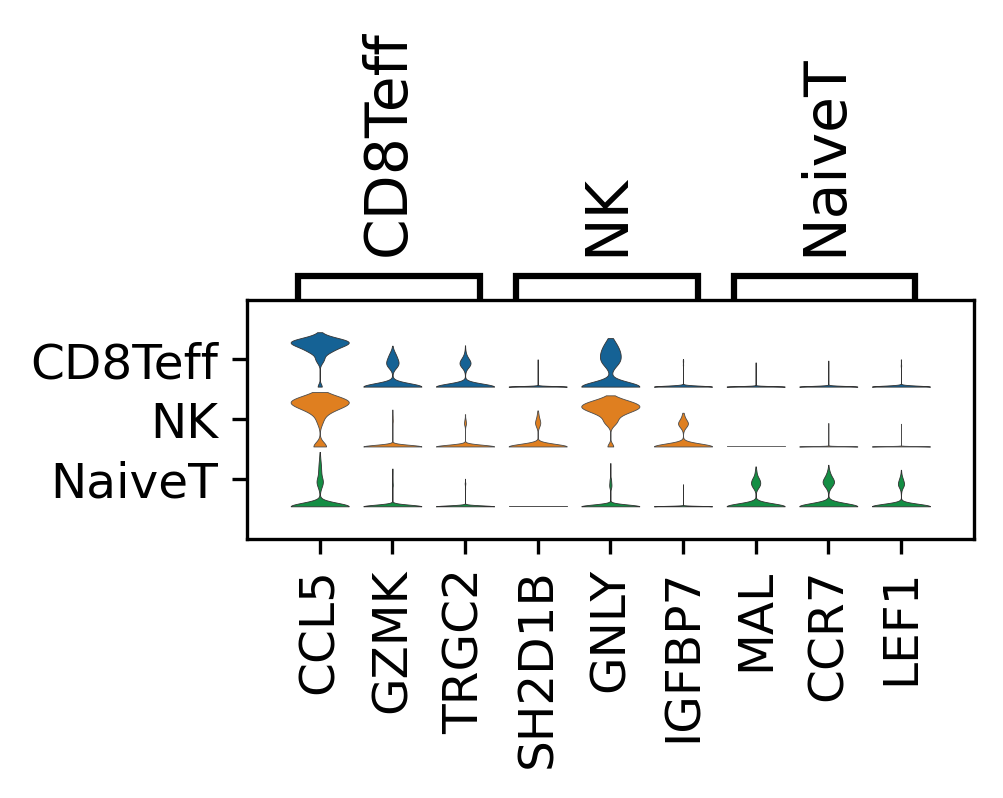

Supplement: Supplementary file 1 [file DataSheet_1.zip › Single-cell sequencing analysis/T cells/P22082602_TopStackedViolin.png]

gname

UMAP2

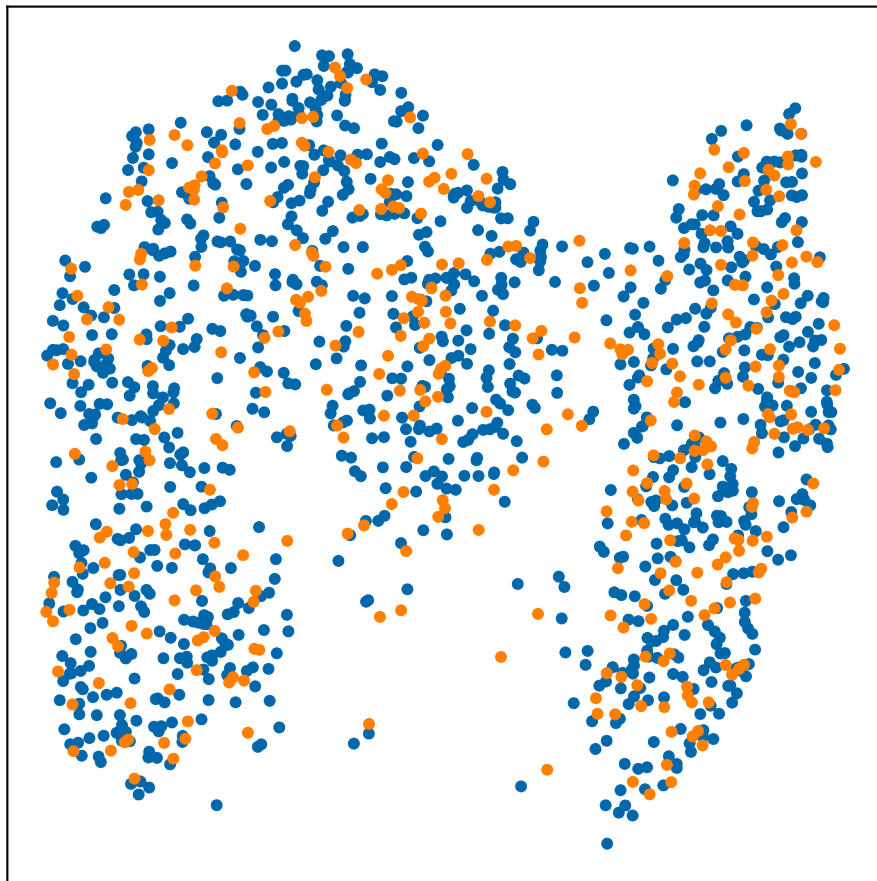

● ZZH20220826-0

● ZZH20220901-7

UMAP1

Supplement: Supplementary file 1 [file DataSheet_1.zip › Single-cell sequencing analysis/T cells/P22082602_umap_groups.pdf]

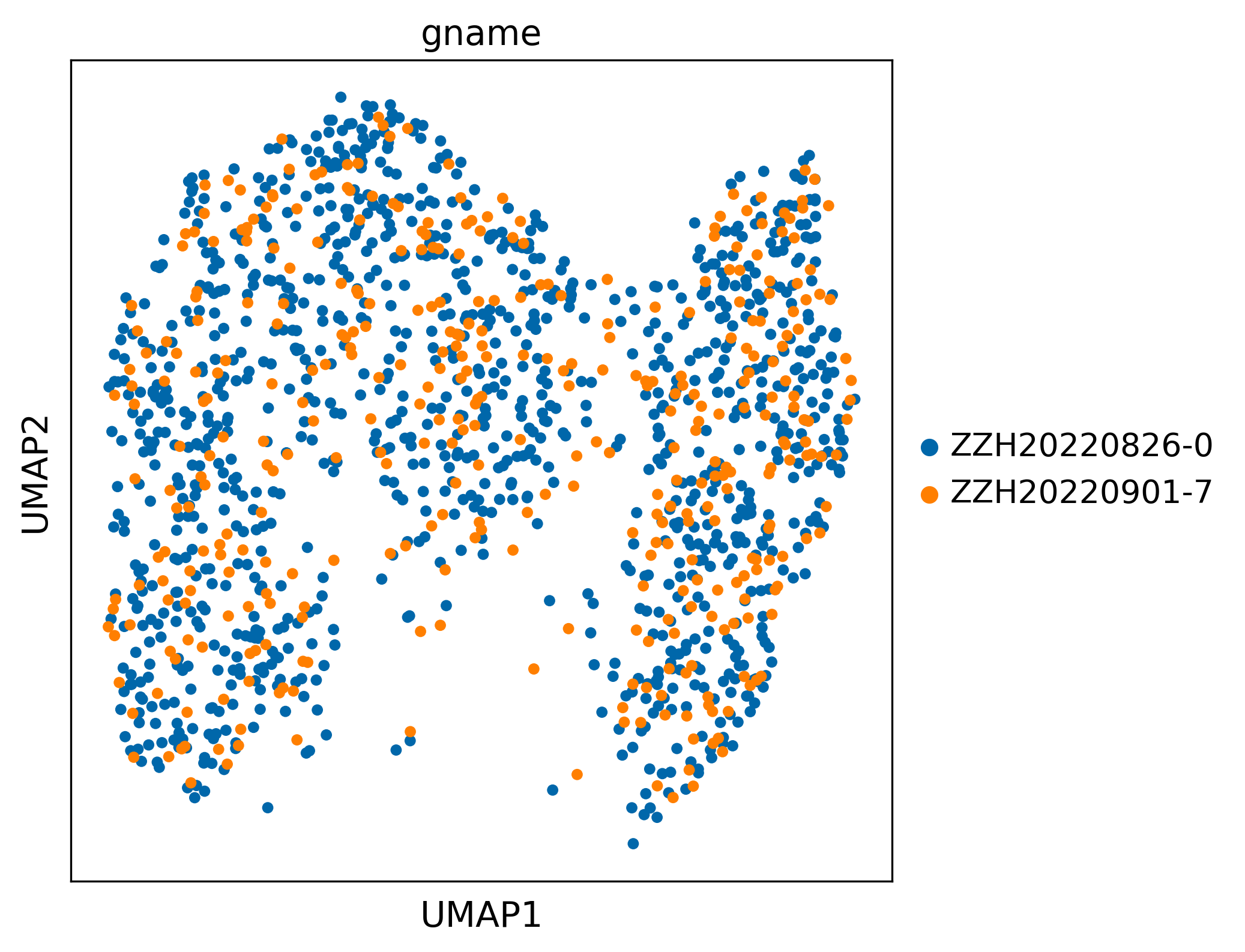

Supplement: Supplementary file 1 [file DataSheet_1.zip › Single-cell sequencing analysis/T cells/P22082602_umap_groups.png]

sample

UMAP2

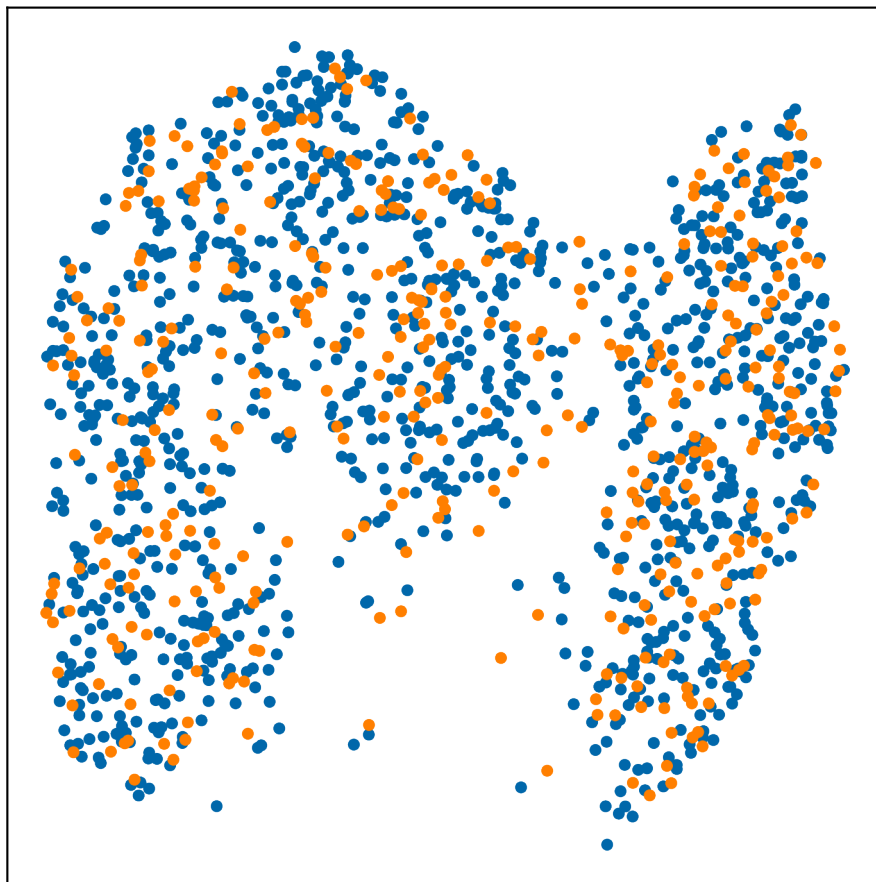

● ZZH20220826-0

● ZZH20220901-7

UMAP1

Supplement: Supplementary file 1 [file DataSheet_1.zip › Single-cell sequencing analysis/T cells/P22082602_umap_samples.pdf]

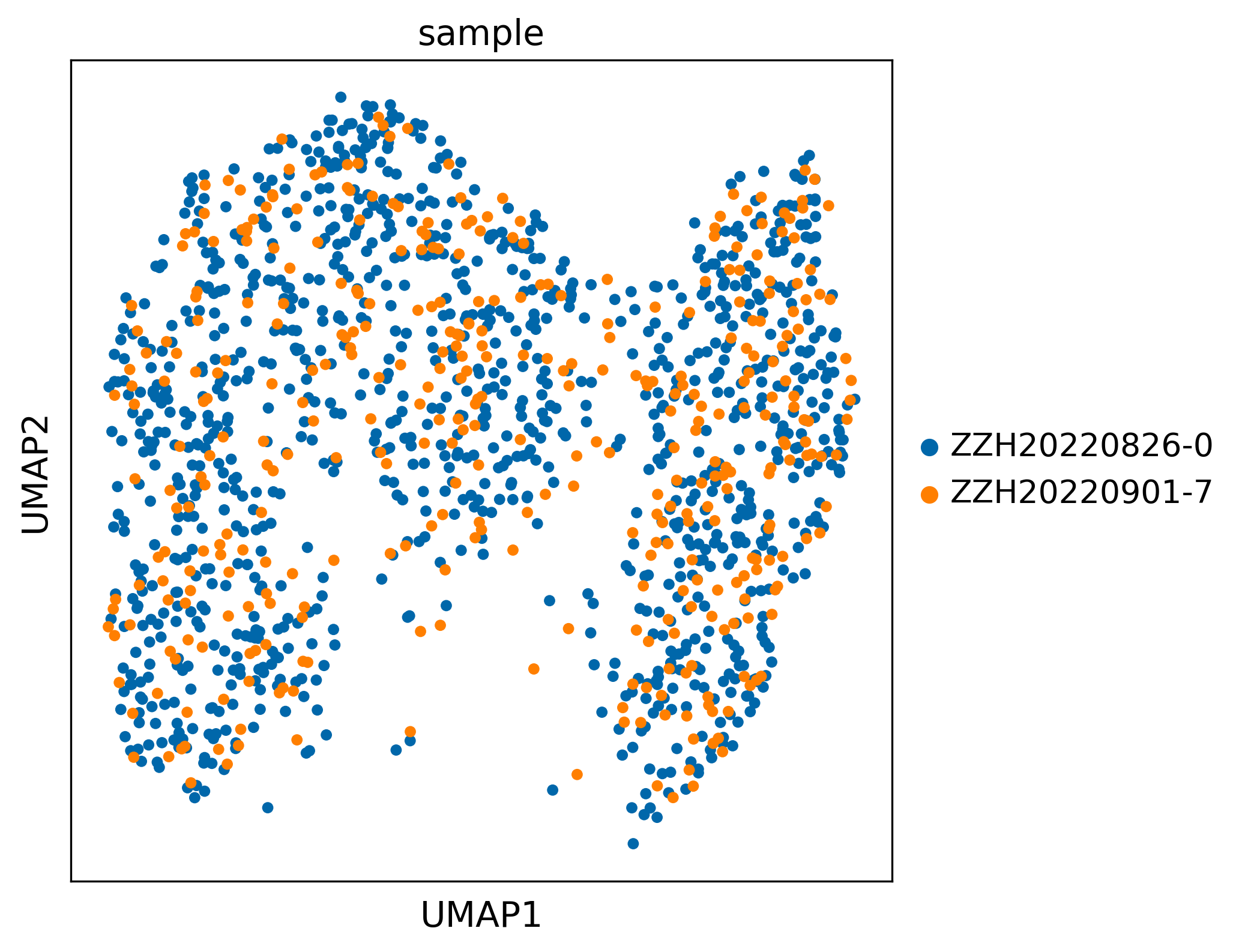

Supplement: Supplementary file 1 [file DataSheet_1.zip › Single-cell sequencing analysis/T cells/P22082602_umap_samples.png]

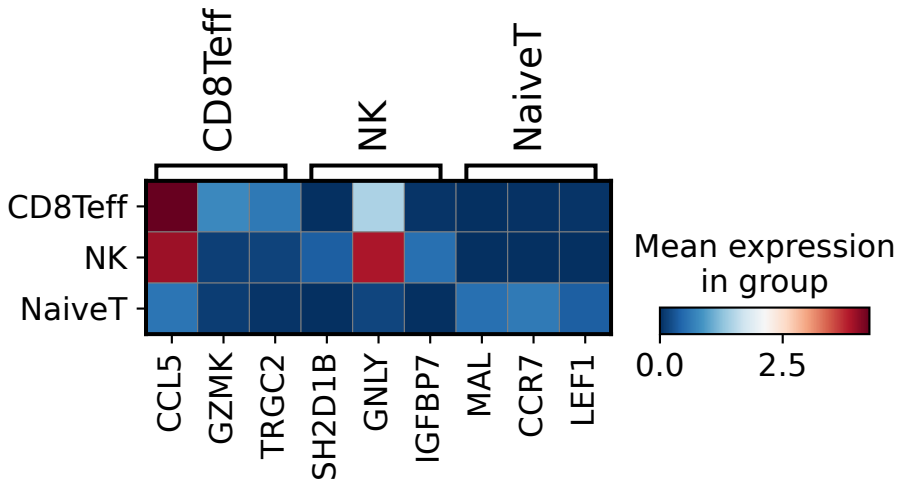

Supplement: Supplementary file 1 [file DataSheet_1.zip › Single-cell sequencing analysis/T cells/P22082602_Zscore_matrixplot.pdf]

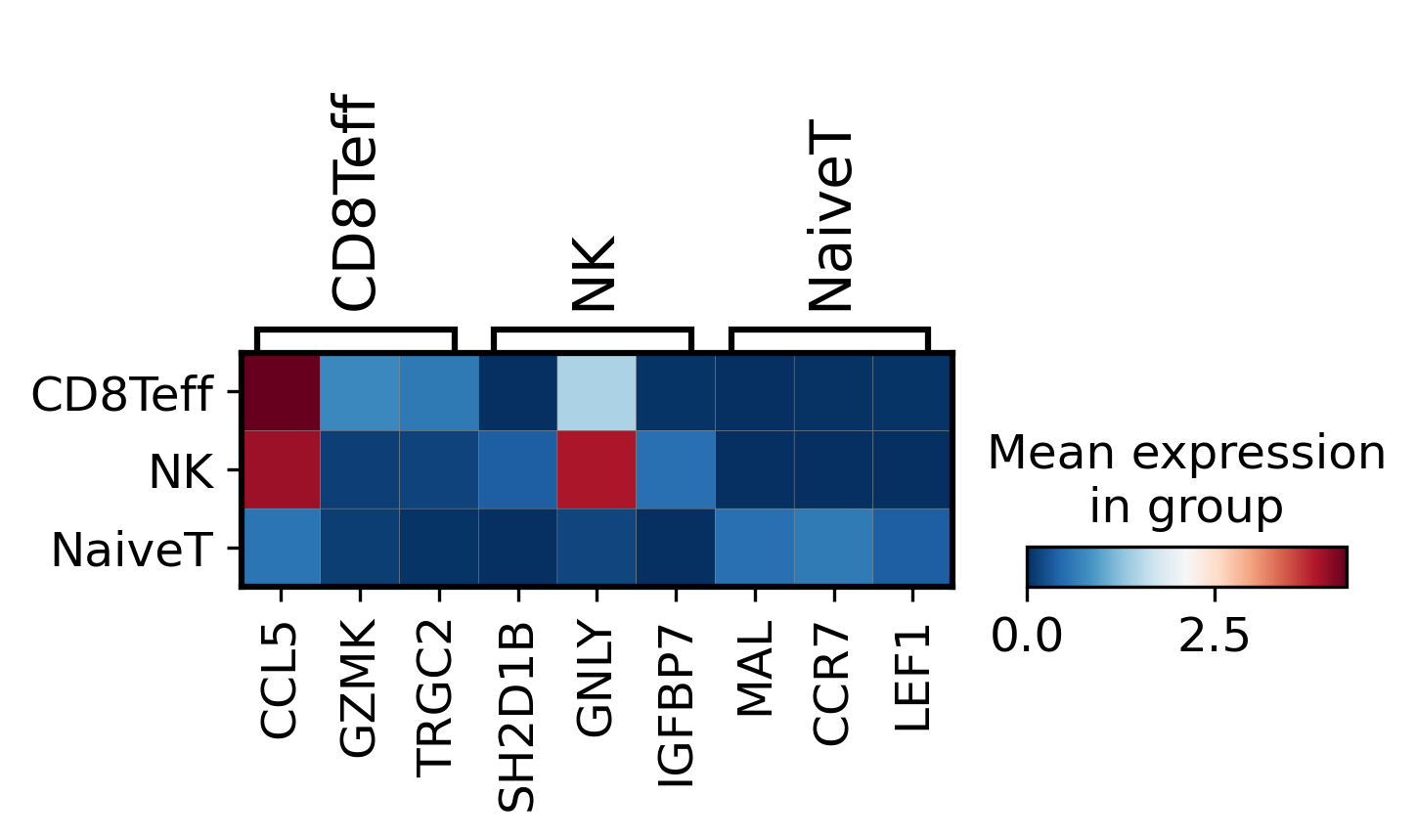

Supplement: Supplementary file 1 [file DataSheet_1.zip › Single-cell sequencing analysis/T cells/P22082602_Zscore_matrixplot.png]
