# Supplementary figures and images for: Porcine anti-human lymphocyte immunoglobulin depletes the lymphocyte population to promote successful kidney transplantation (part 1 of 2)
Source: Front Immunol. 2023 Mar 9;14:1124790. doi: 10.3389/fimmu.2023.1124790 (PMC10033525; doi:10.3389/fimmu.2023.1124790)

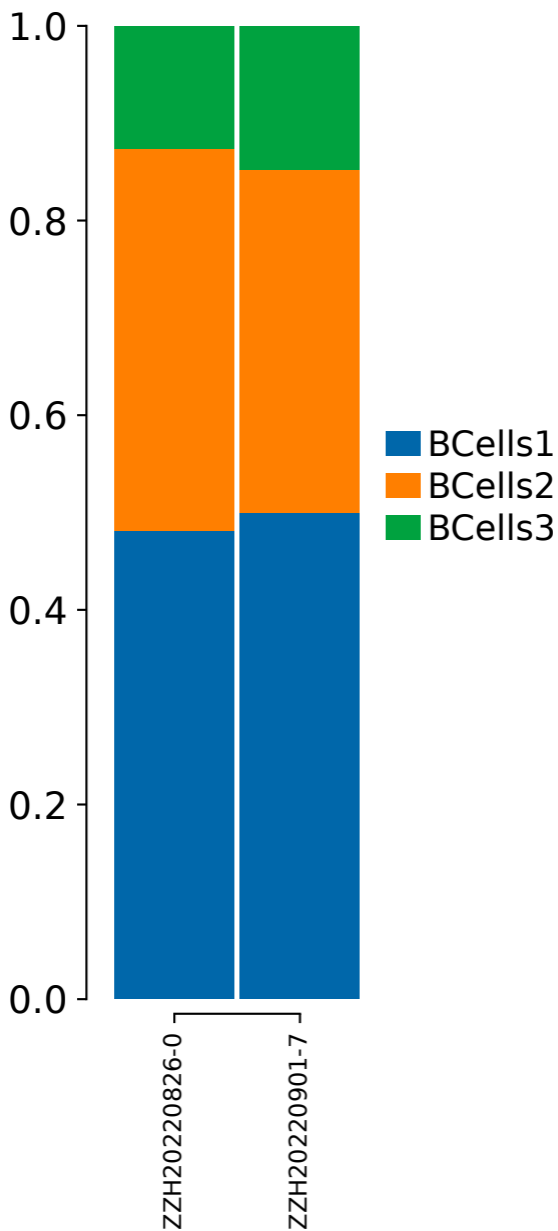

Supplement: Supplementary file 1 [file DataSheet_1.zip › Single-cell sequencing analysis/B cells/P22082602_group_PercentPerCell.pdf]

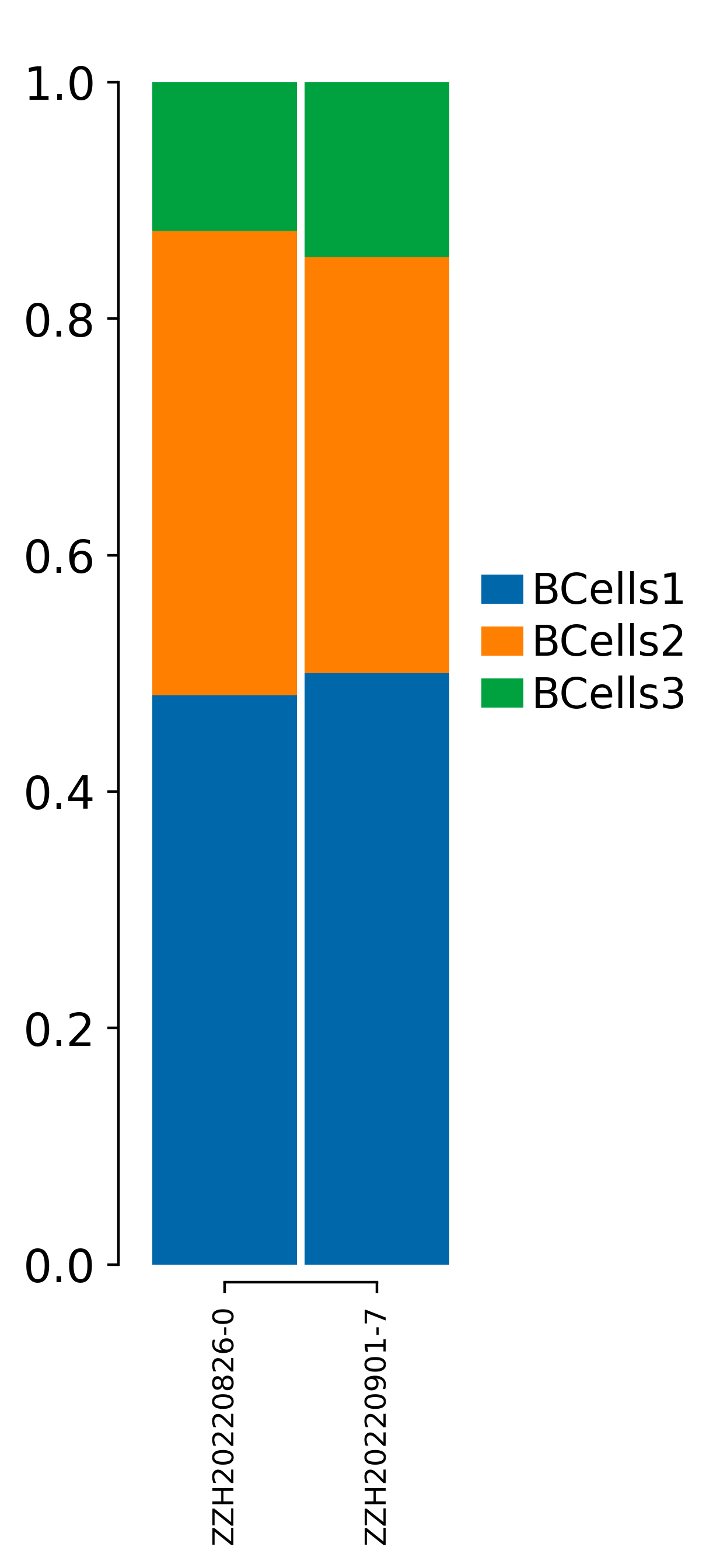

Supplement: Supplementary file 1 [file DataSheet_1.zip › Single-cell sequencing analysis/B cells/P22082602_group_PercentPerCell.png]

cluster

UMAP2

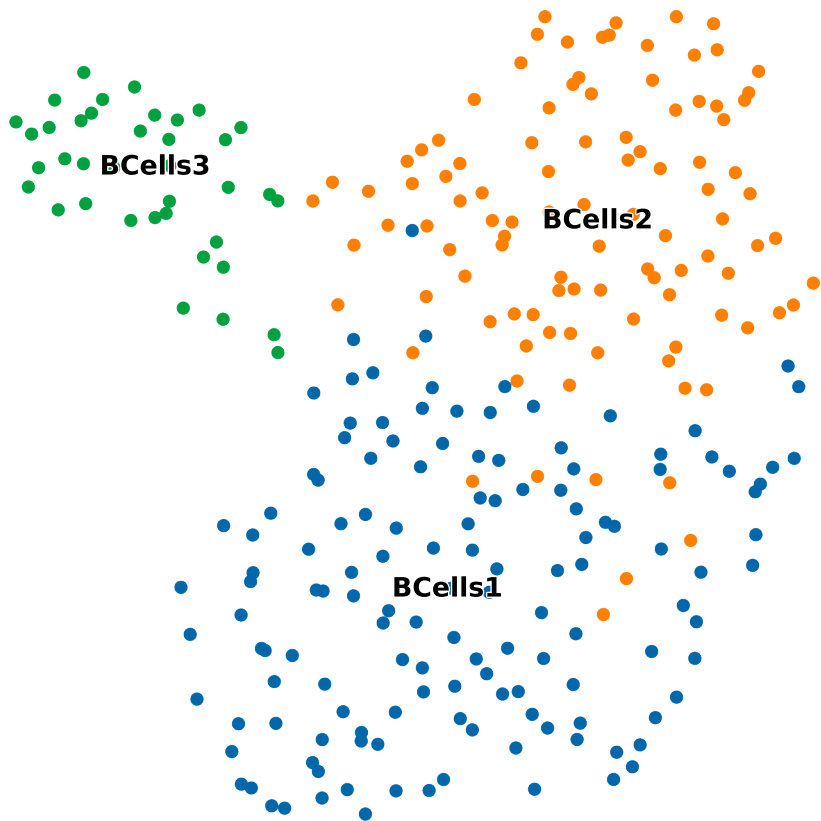

UMAP1

Supplement: Supplementary file 1 [file DataSheet_1.zip › Single-cell sequencing analysis/B cells/P22082602_labumap.pdf]

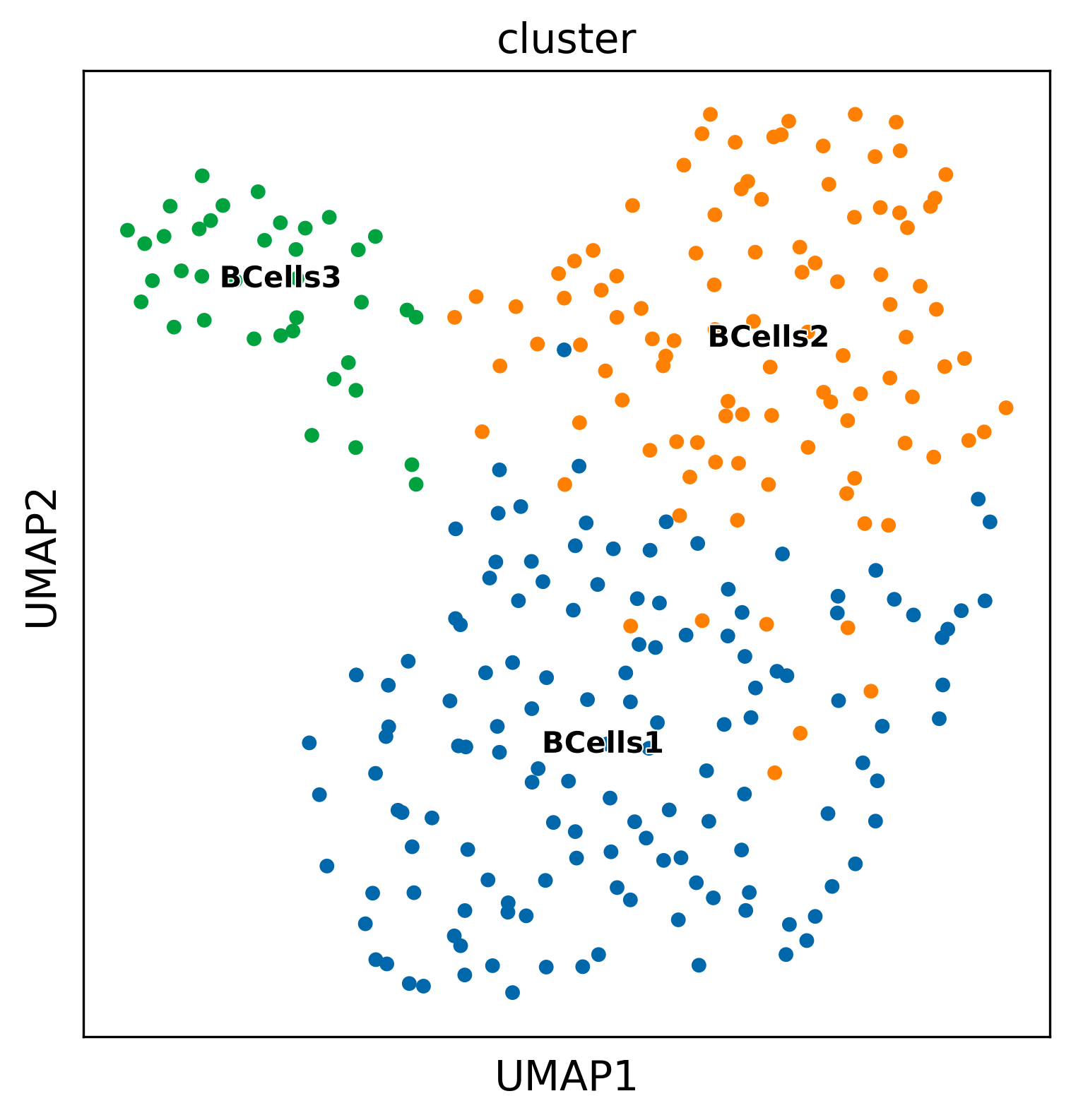

Supplement: Supplementary file 1 [file DataSheet_1.zip › Single-cell sequencing analysis/B cells/P22082602_labumap.png]

cluster

UMAP2

● BCells1  
● BCells2  
● BCells3

UMAP1

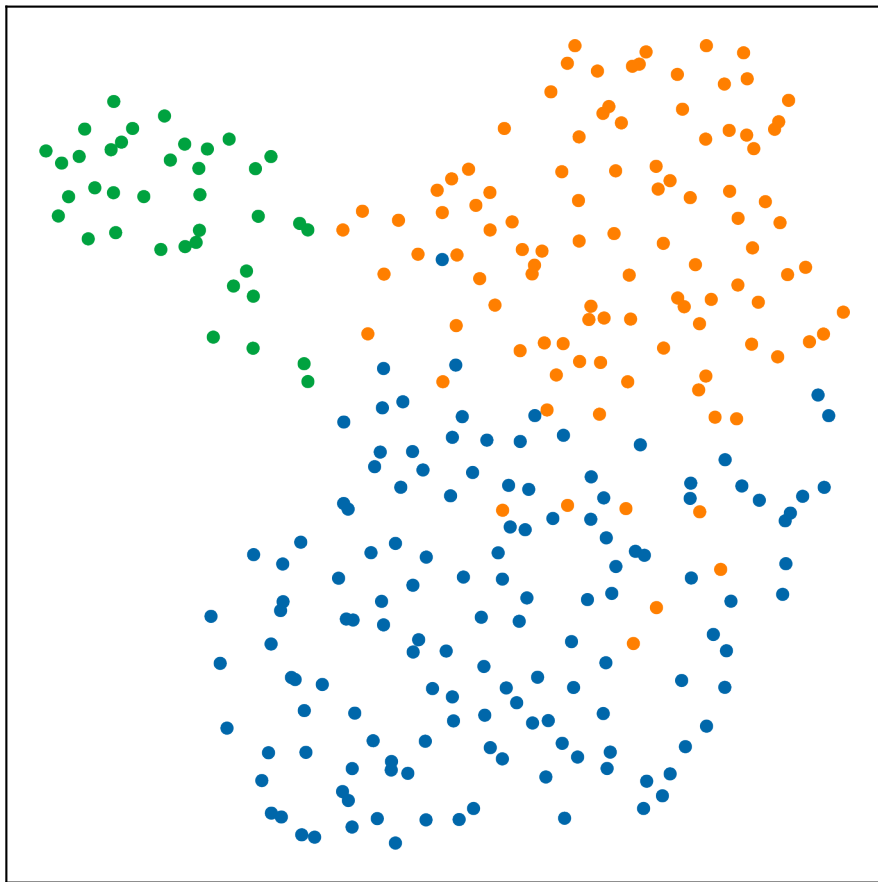

Supplement: Supplementary file 1 [file DataSheet_1.zip › Single-cell sequencing analysis/B cells/P22082602_rlabumap.pdf]

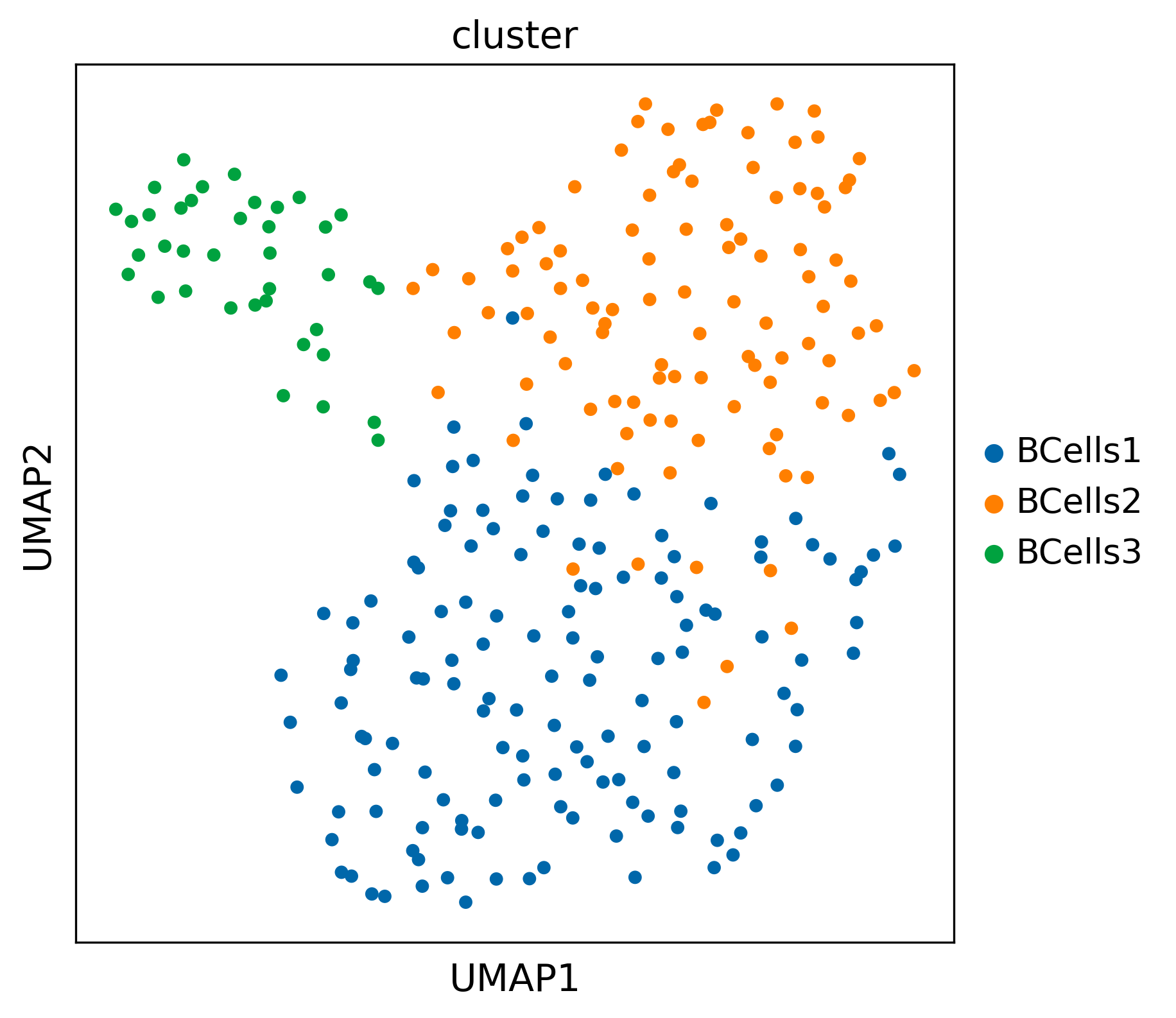

Supplement: Supplementary file 1 [file DataSheet_1.zip › Single-cell sequencing analysis/B cells/P22082602_rlabumap.png]

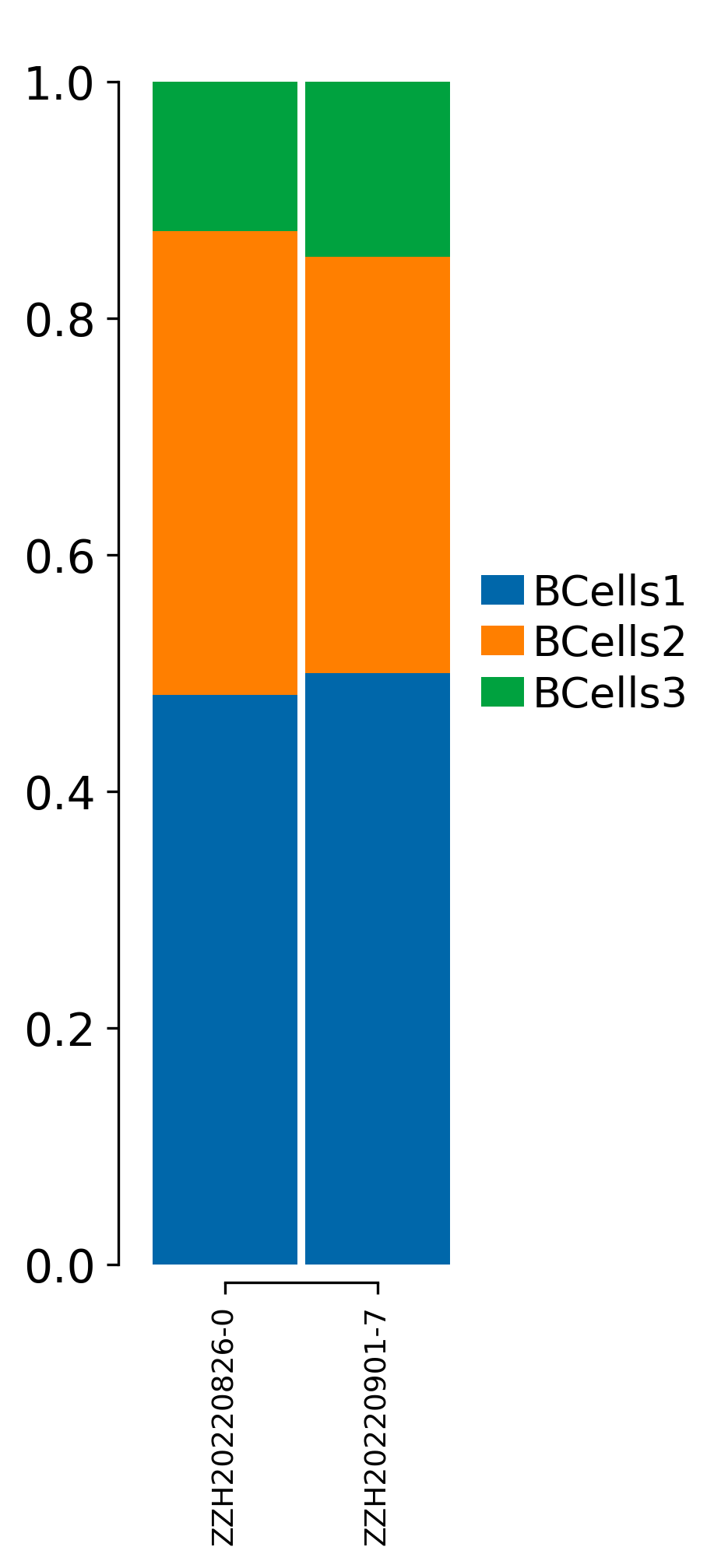

Supplement: Supplementary file 1 [file DataSheet_1.zip › Single-cell sequencing analysis/B cells/P22082602_sample_PercentPerCell.png]

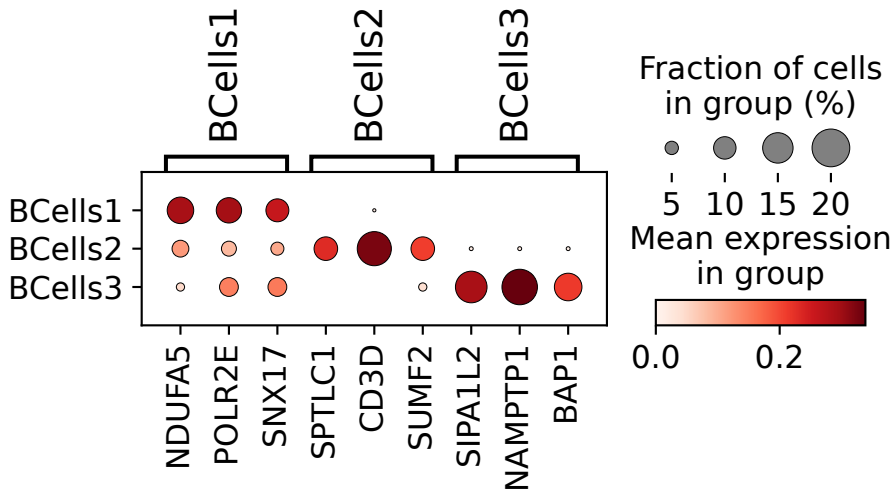

Supplement: Supplementary file 1 [file DataSheet_1.zip › Single-cell sequencing analysis/B cells/P22082602_TopMarkergenedotplot.pdf]

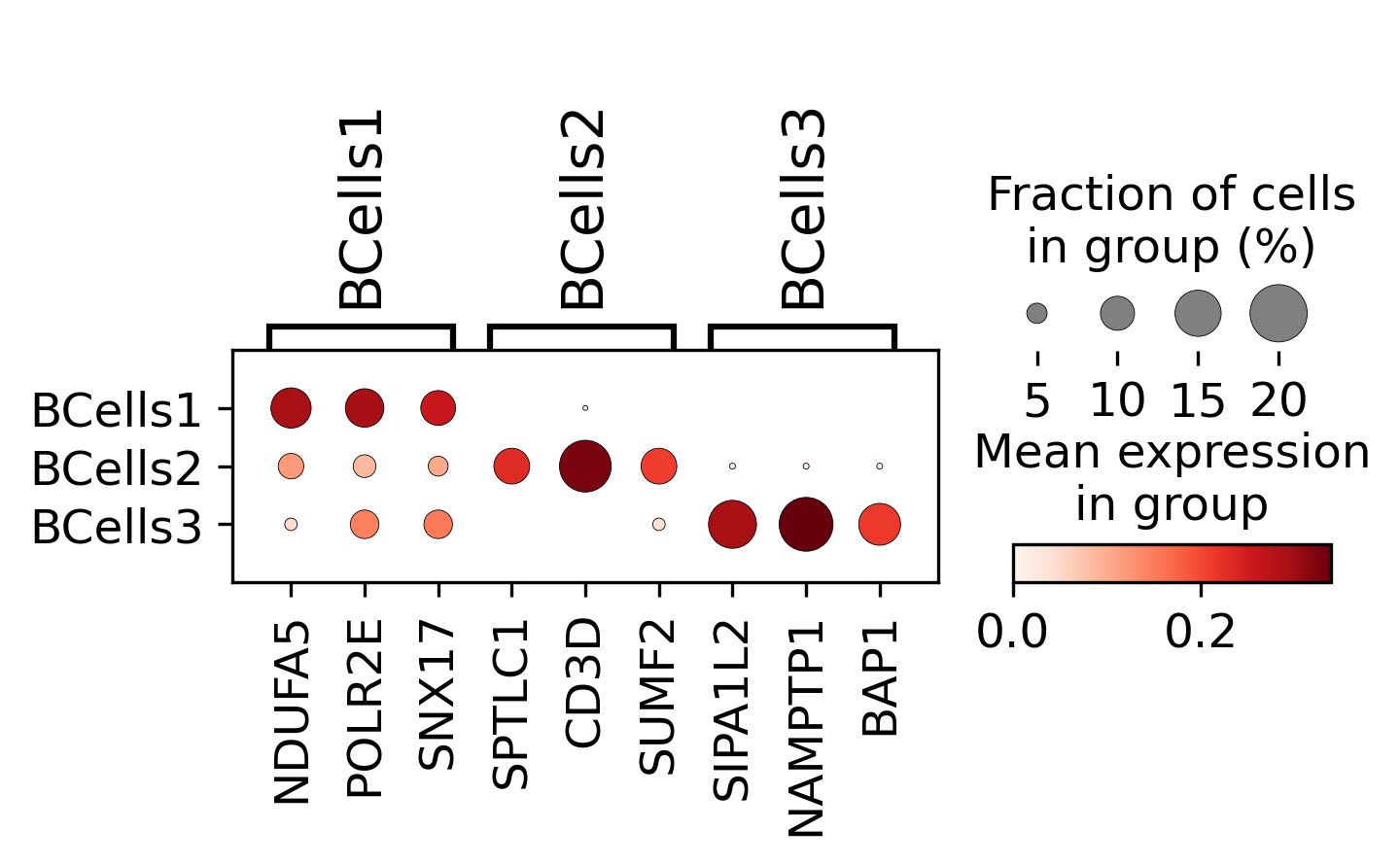

Supplement: Supplementary file 1 [file DataSheet_1.zip › Single-cell sequencing analysis/B cells/P22082602_TopMarkergenedotplot.png]

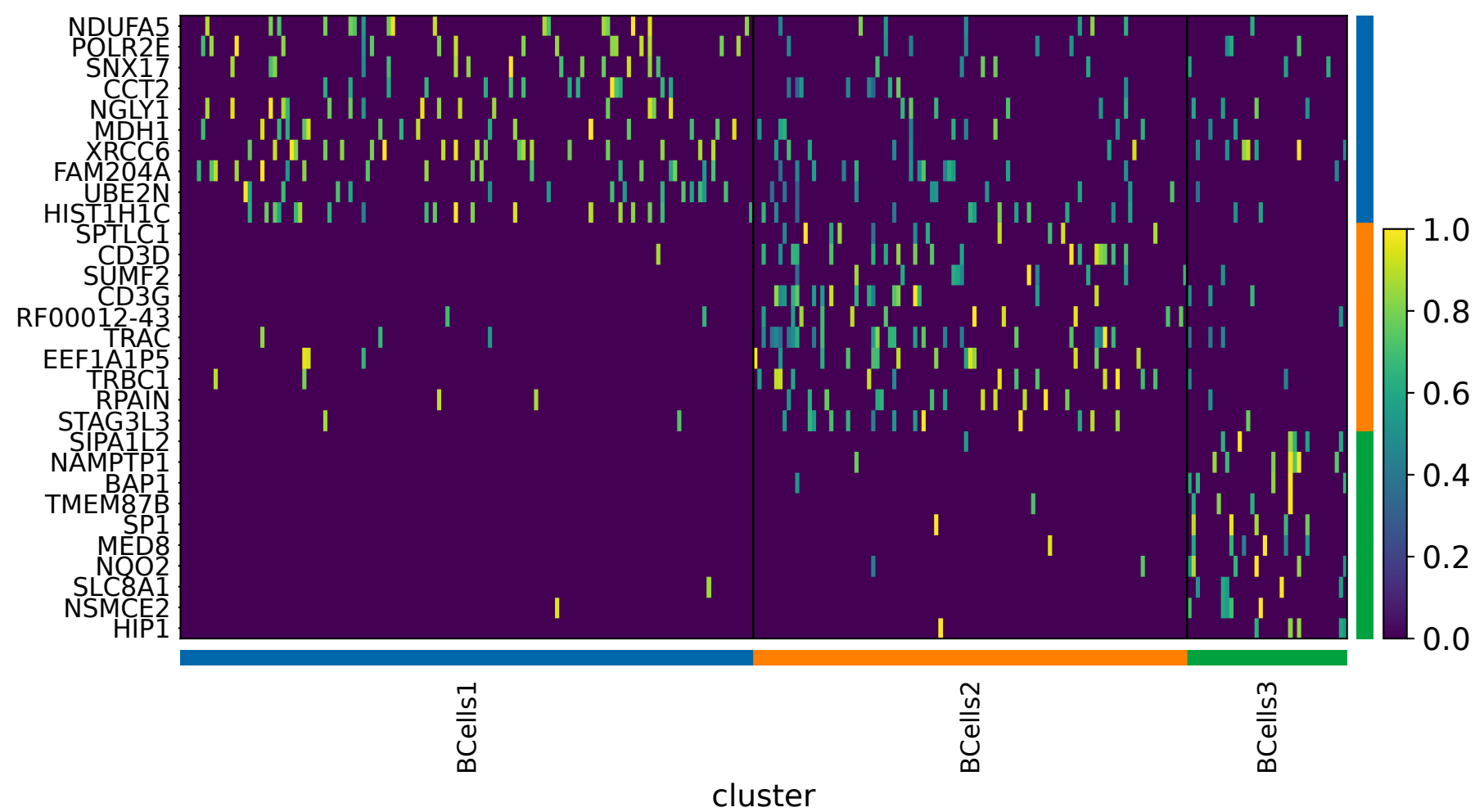

Supplement: Supplementary file 1 [file DataSheet_1.zip › Single-cell sequencing analysis/B cells/P22082602_TopMarkergeneHeatmap.pdf]

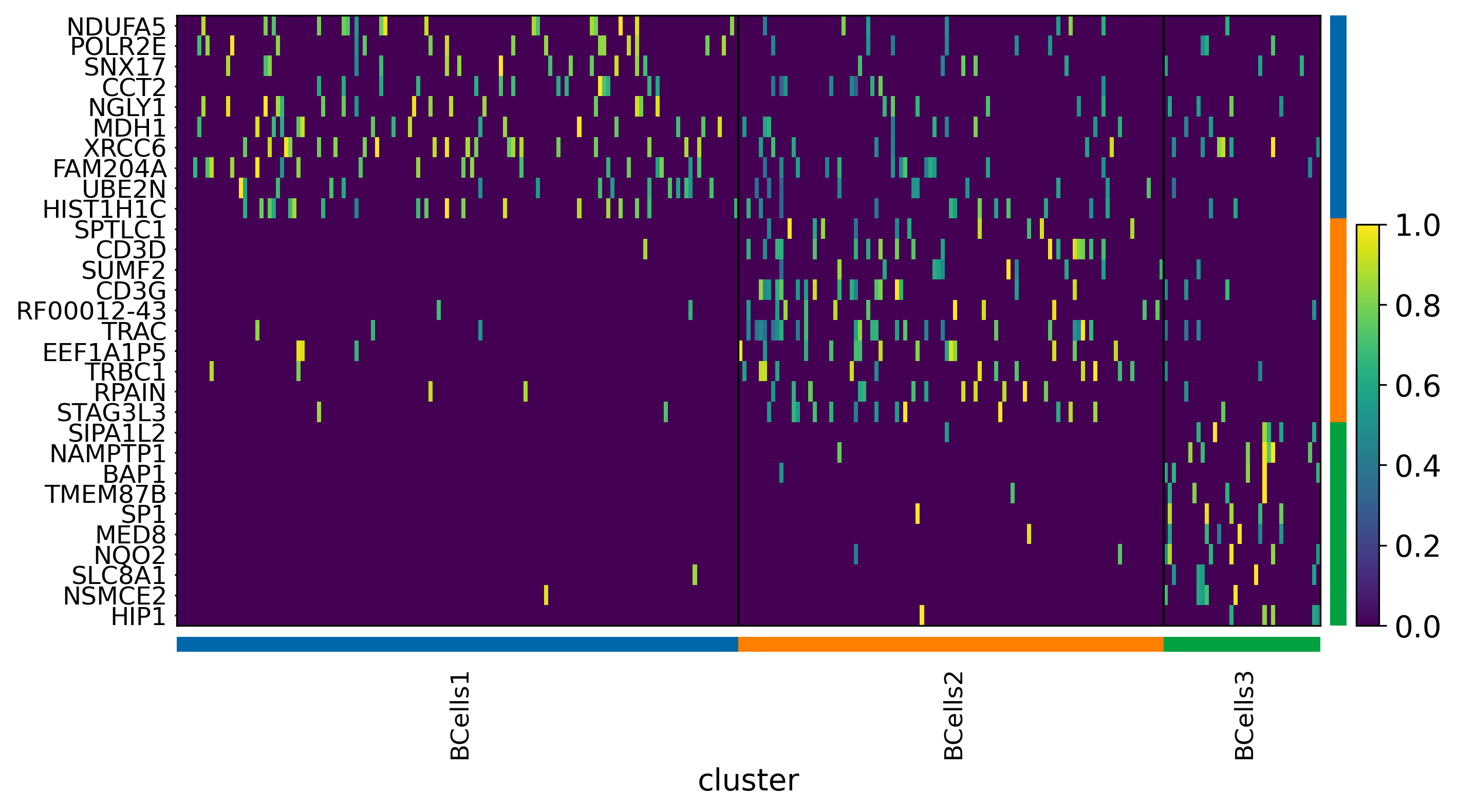

Supplement: Supplementary file 1 [file DataSheet_1.zip › Single-cell sequencing analysis/B cells/P22082602_TopMarkergeneHeatmap.png]

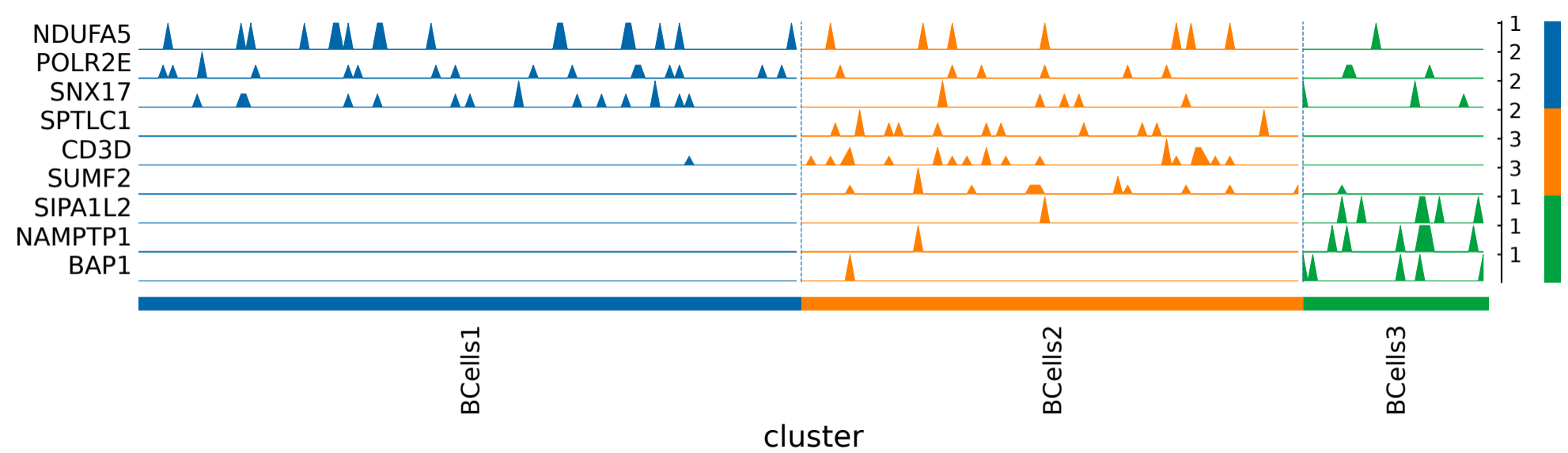

Supplement: Supplementary file 1 [file DataSheet_1.zip › Single-cell sequencing analysis/B cells/P22082602_TopMarkergeneTracksplot.pdf]

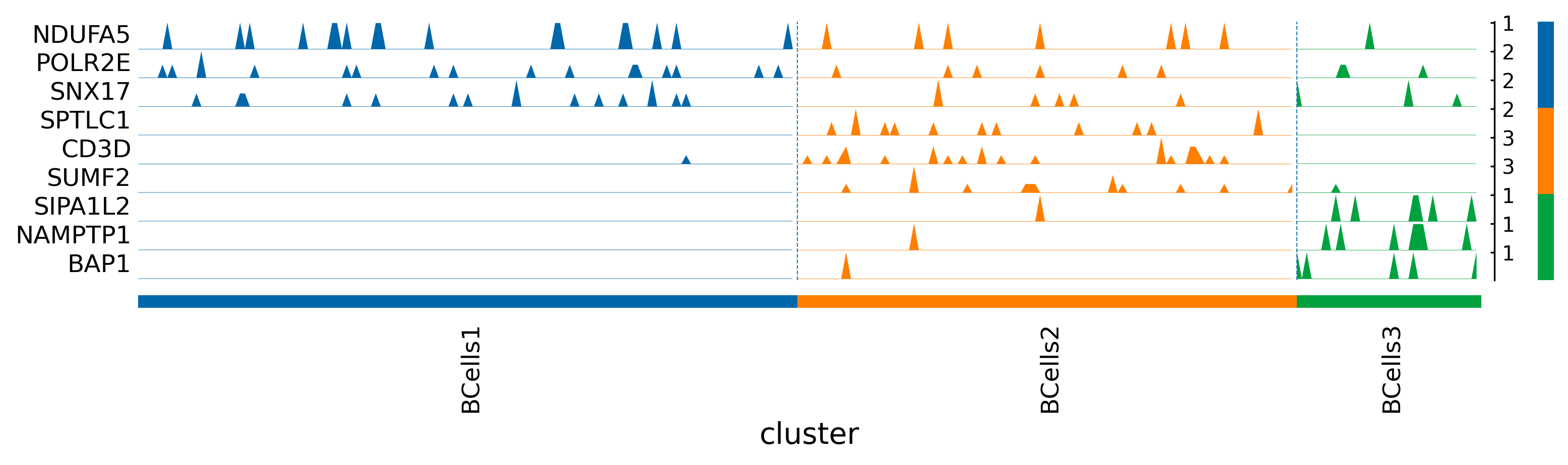

Supplement: Supplementary file 1 [file DataSheet_1.zip › Single-cell sequencing analysis/B cells/P22082602_TopMarkergeneTracksplot.png]

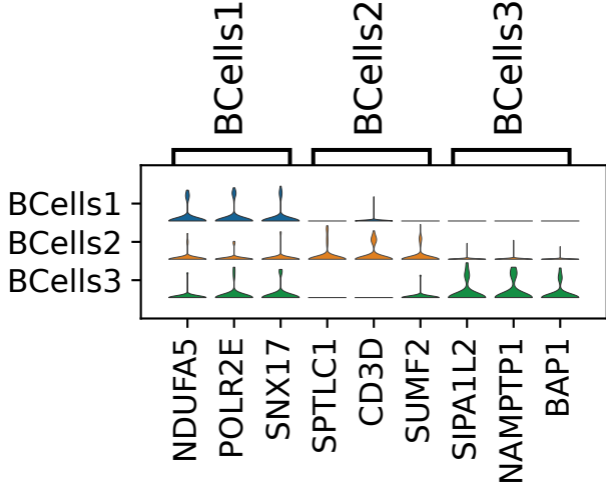

Supplement: Supplementary file 1 [file DataSheet_1.zip › Single-cell sequencing analysis/B cells/P22082602_TopStackedViolin.pdf]

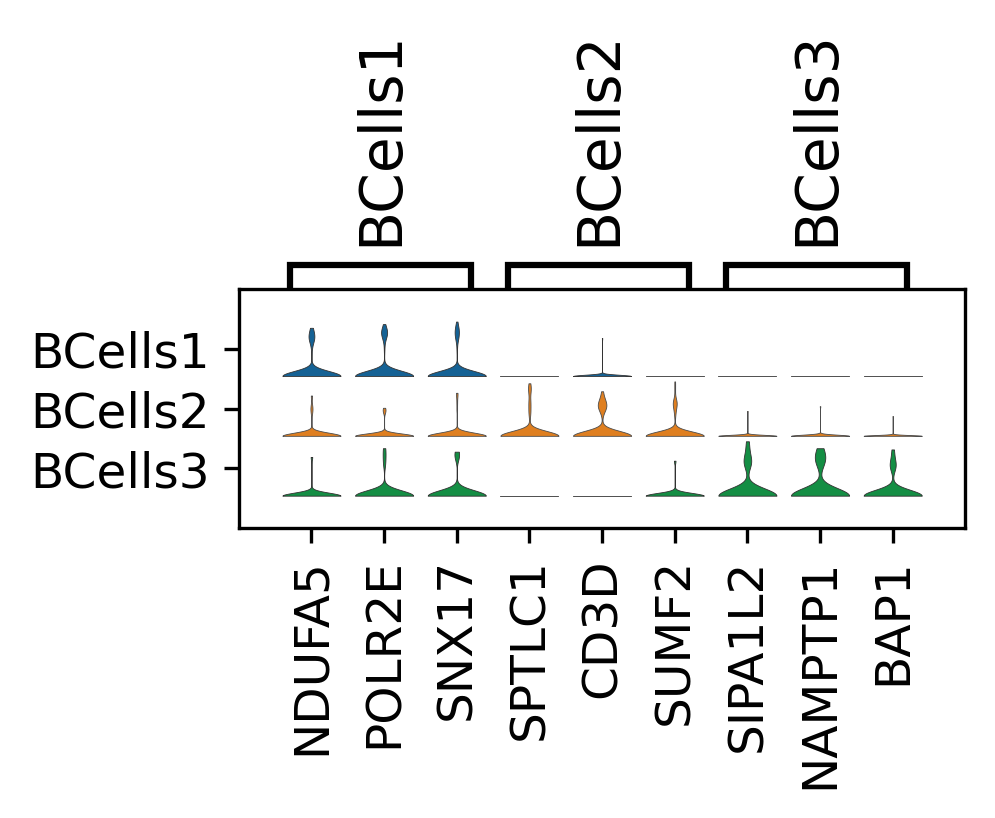

Supplement: Supplementary file 1 [file DataSheet_1.zip › Single-cell sequencing analysis/B cells/P22082602_TopStackedViolin.png]

gname

UMAP2

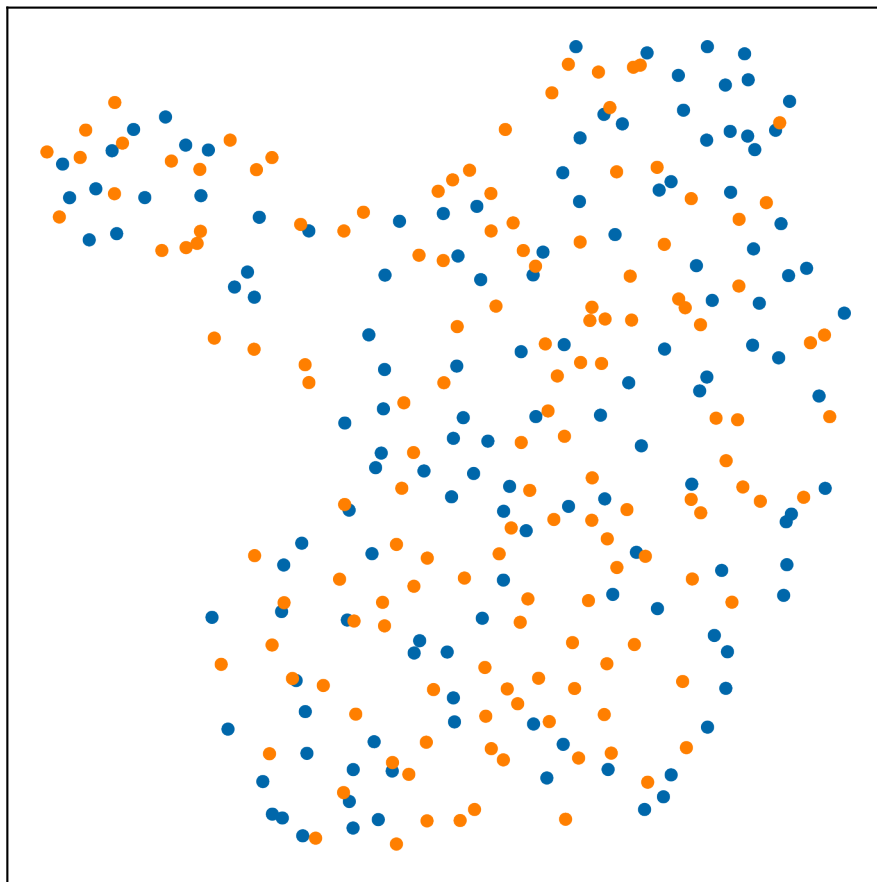

● ZZH20220826-0

● ZZH20220901-7

UMAP1

Supplement: Supplementary file 1 [file DataSheet_1.zip › Single-cell sequencing analysis/B cells/P22082602_umap_groups.pdf]

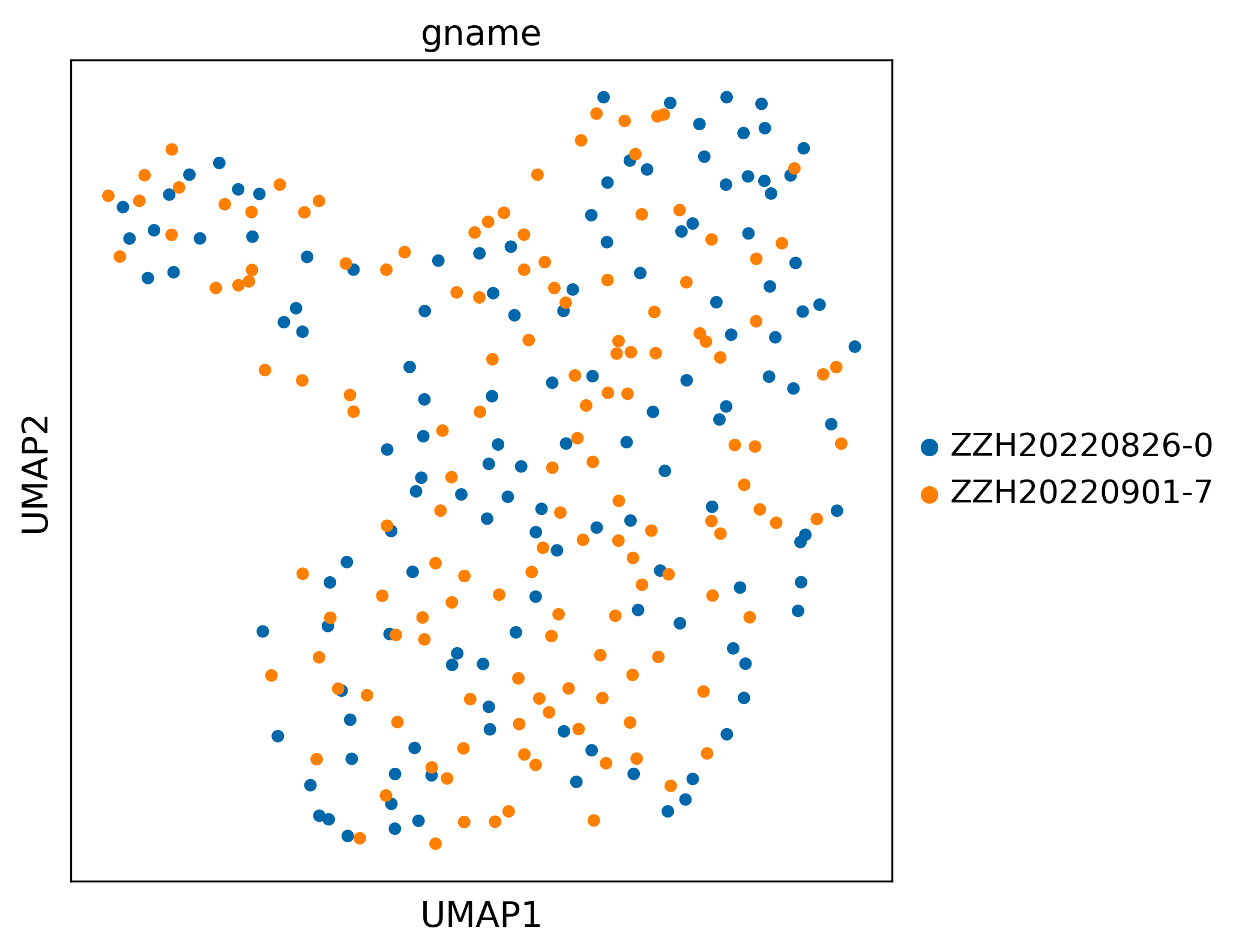

Supplement: Supplementary file 1 [file DataSheet_1.zip › Single-cell sequencing analysis/B cells/P22082602_umap_groups.png]

sample

UMAP2

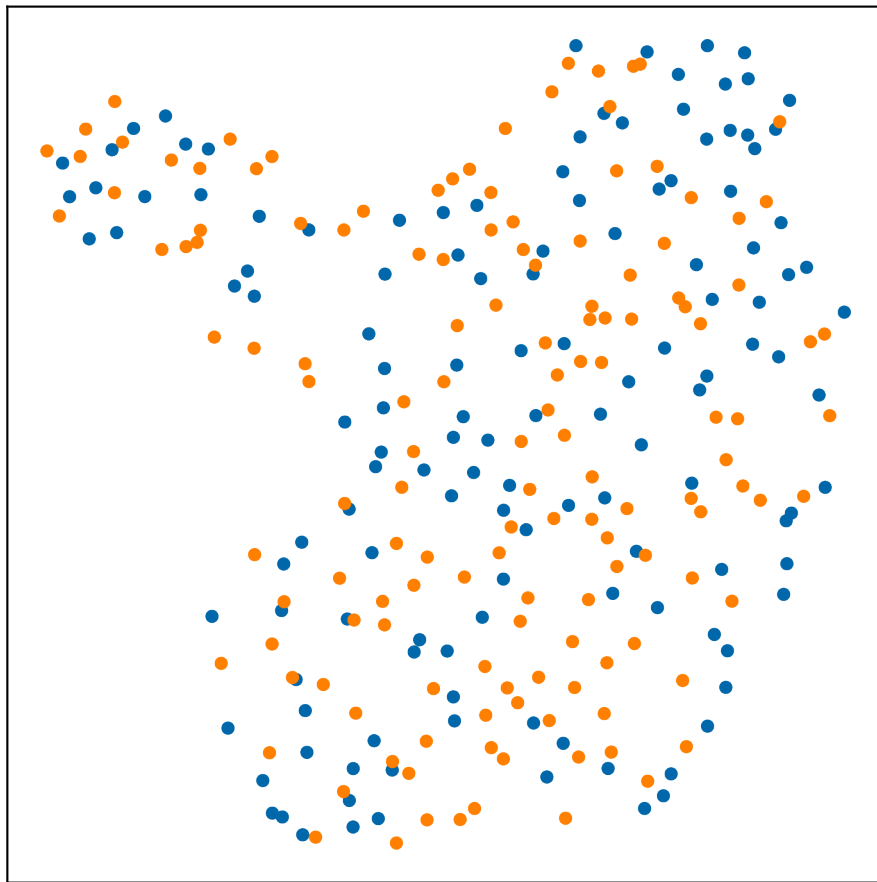

UMAP1

● ZZH20220826-0

● ZZH20220901-7

Supplement: Supplementary file 1 [file DataSheet_1.zip › Single-cell sequencing analysis/B cells/P22082602_umap_samples.pdf]

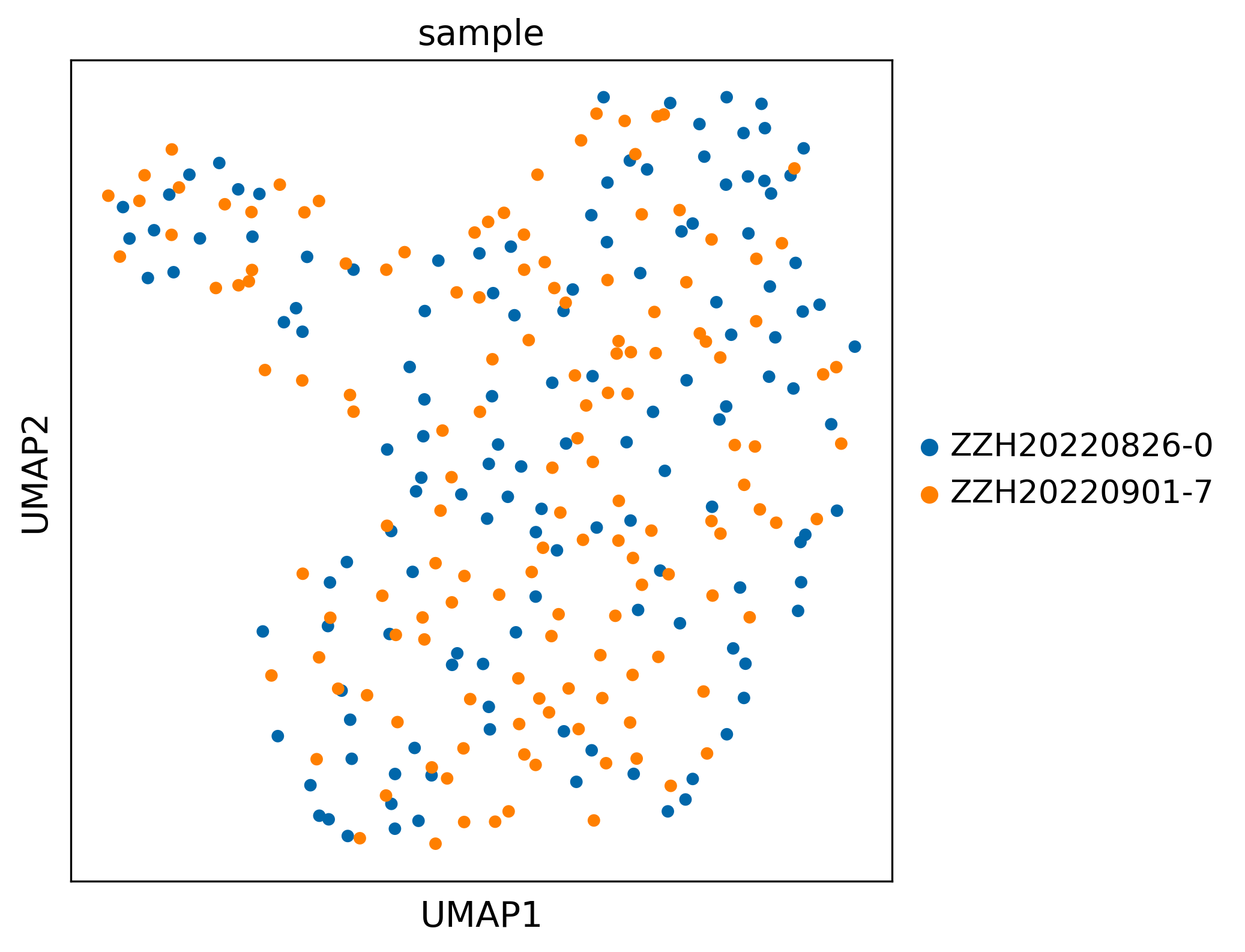

Supplement: Supplementary file 1 [file DataSheet_1.zip › Single-cell sequencing analysis/B cells/P22082602_umap_samples.png]

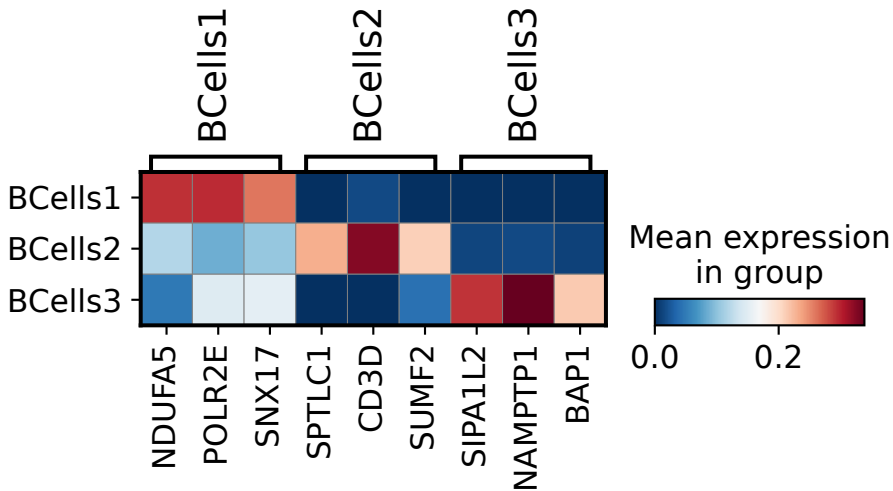

Supplement: Supplementary file 1 [file DataSheet_1.zip › Single-cell sequencing analysis/B cells/P22082602_Zscore_matrixplot.pdf]

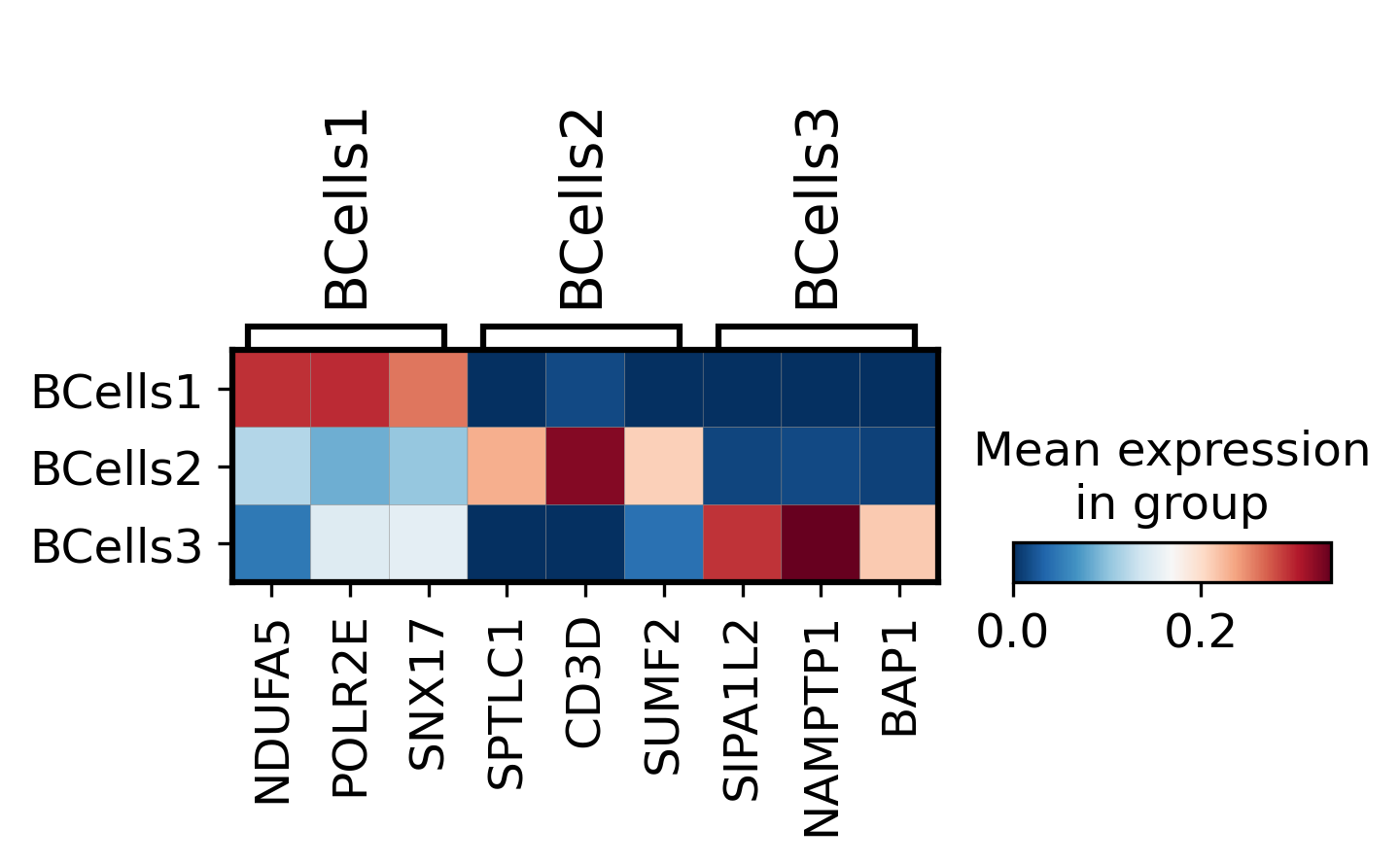

Supplement: Supplementary file 1 [file DataSheet_1.zip › Single-cell sequencing analysis/B cells/P22082602_Zscore_matrixplot.png]

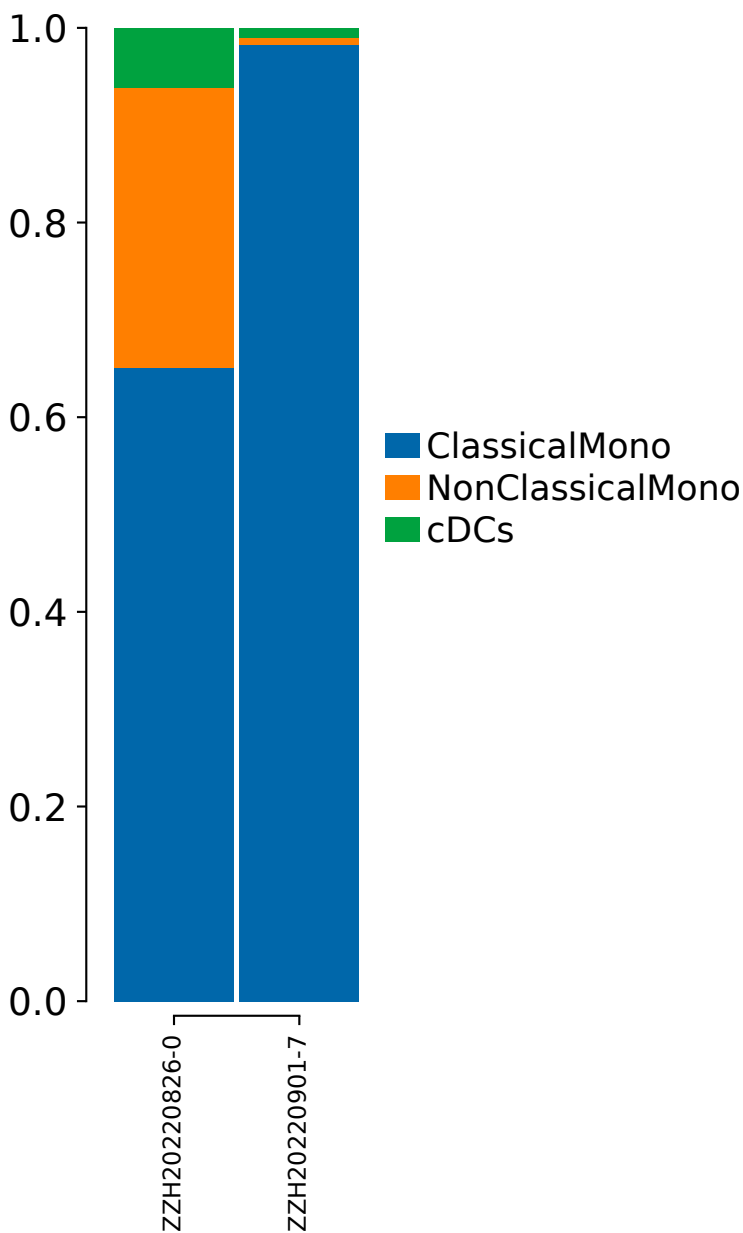

Supplement: Supplementary file 1 [file DataSheet_1.zip › Single-cell sequencing analysis/MPs/P22082602_group_PercentPerCell.pdf]

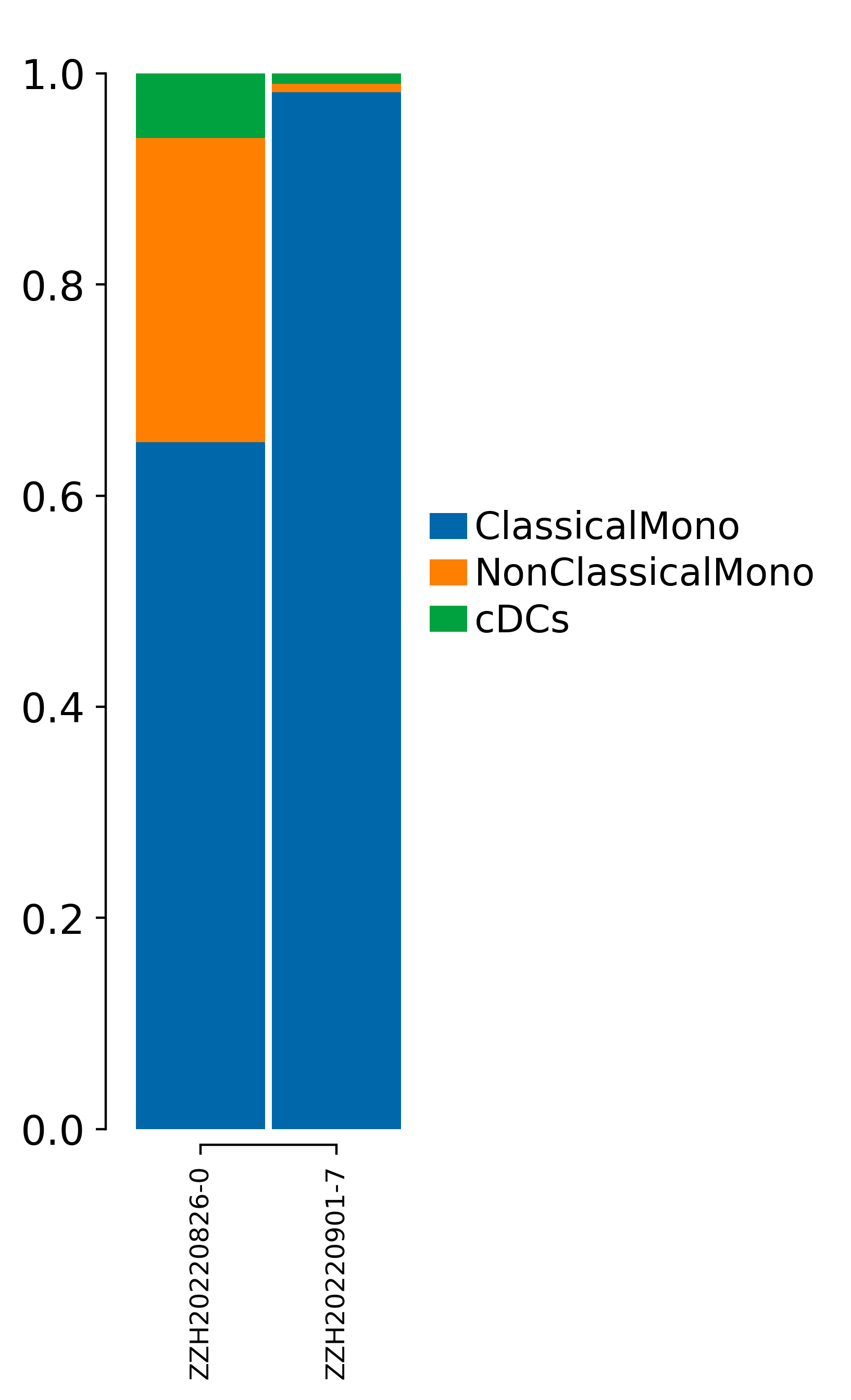

Supplement: Supplementary file 1 [file DataSheet_1.zip › Single-cell sequencing analysis/MPs/P22082602_group_PercentPerCell.png]

cluster

UMAP2

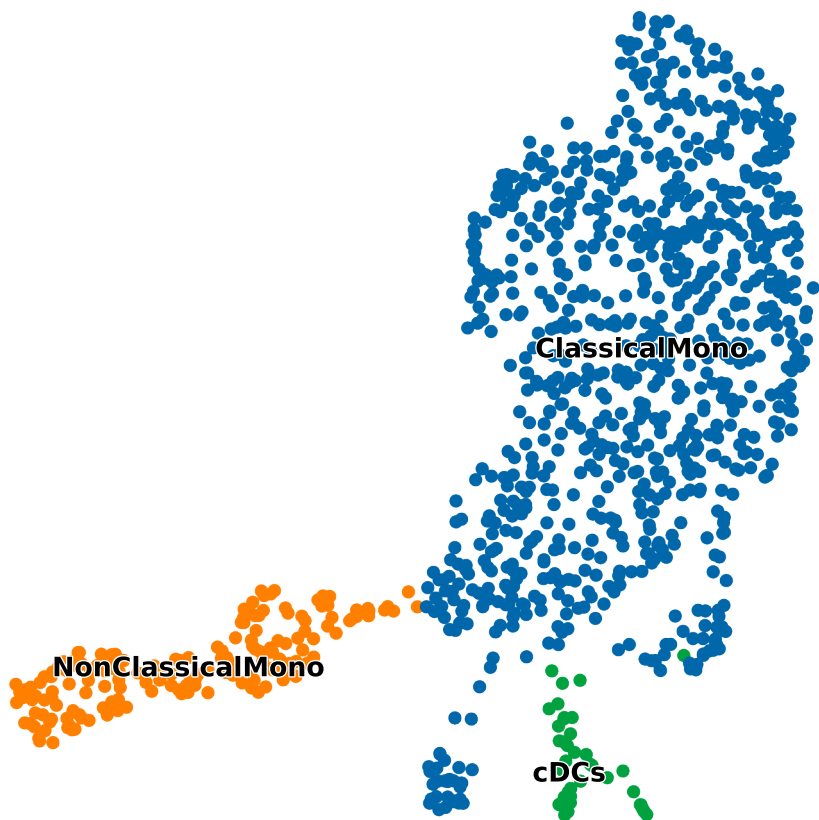

UMAP1

Supplement: Supplementary file 1 [file DataSheet_1.zip › Single-cell sequencing analysis/MPs/P22082602_labumap.pdf]

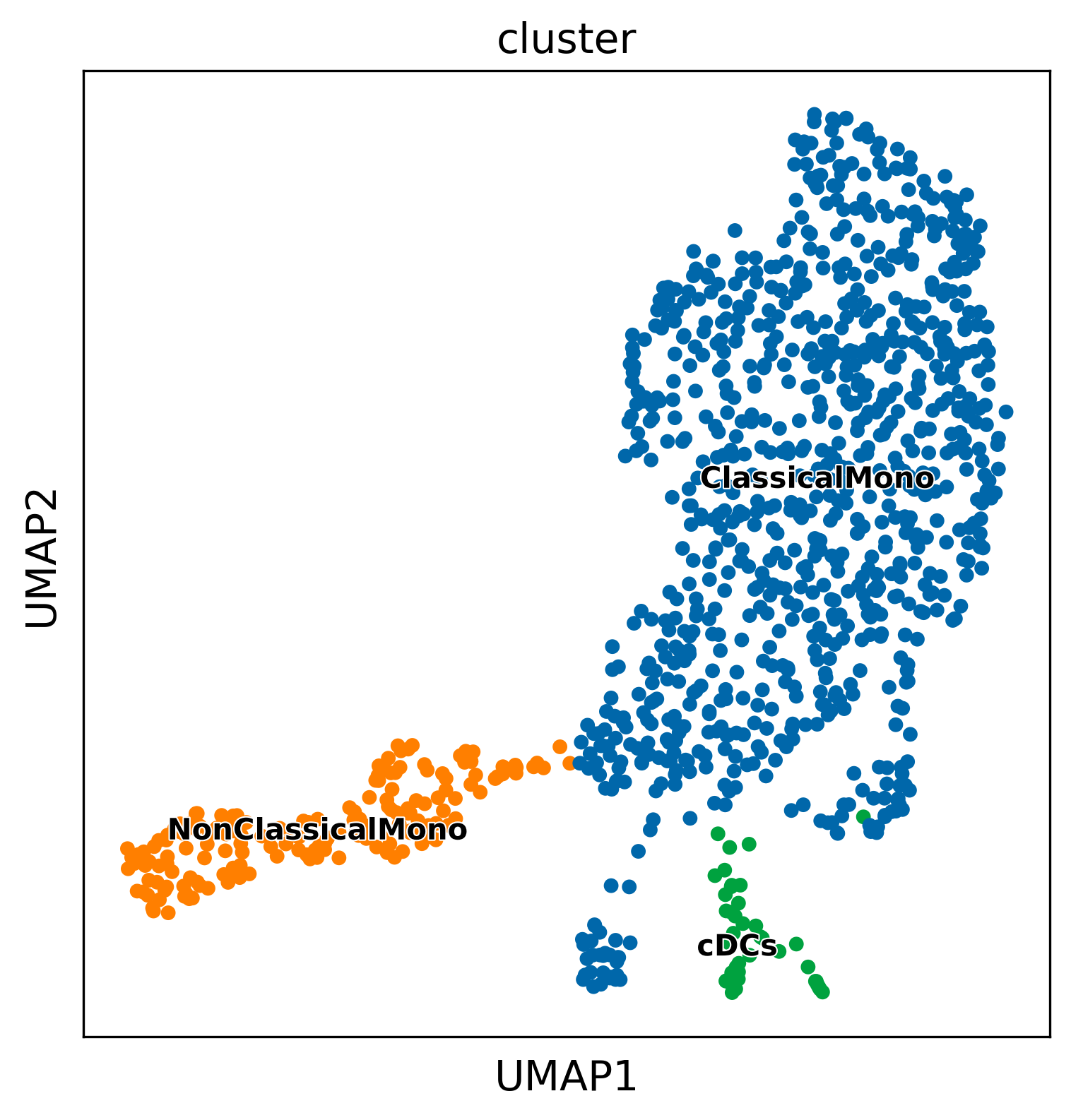

Supplement: Supplementary file 1 [file DataSheet_1.zip › Single-cell sequencing analysis/MPs/P22082602_labumap.png]

cluster

UMAP2

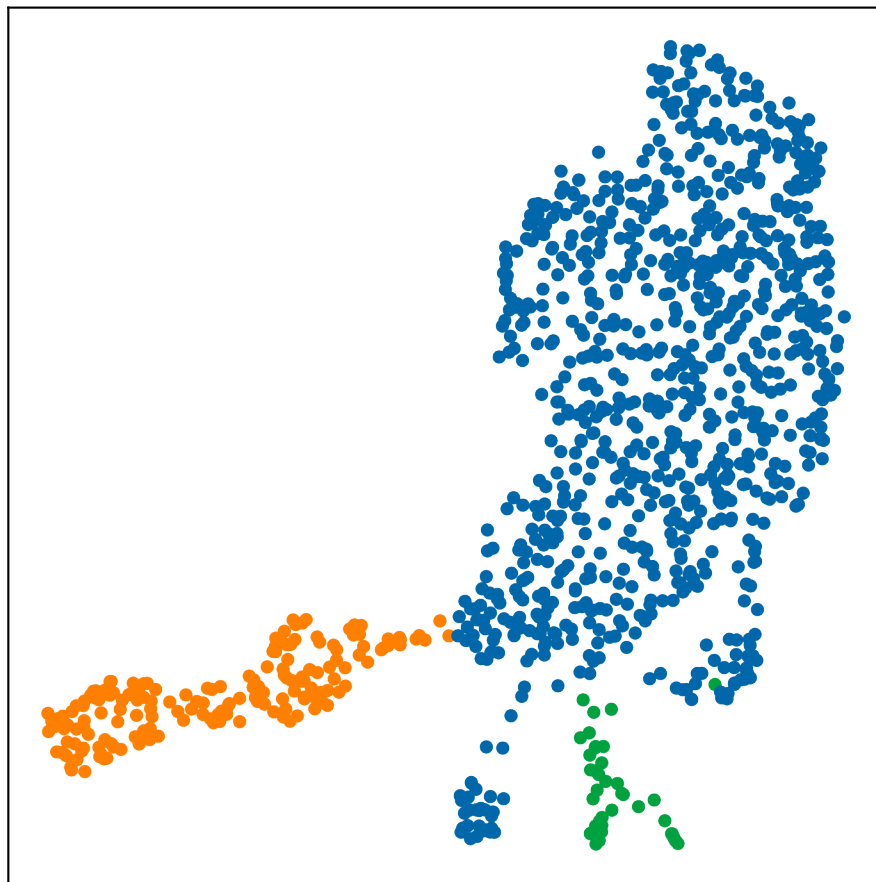

UMAP1

- ClassicalMono
- NonClassicalMono
- cDCs

Supplement: Supplementary file 1 [file DataSheet_1.zip › Single-cell sequencing analysis/MPs/P22082602_rlabumap.pdf]

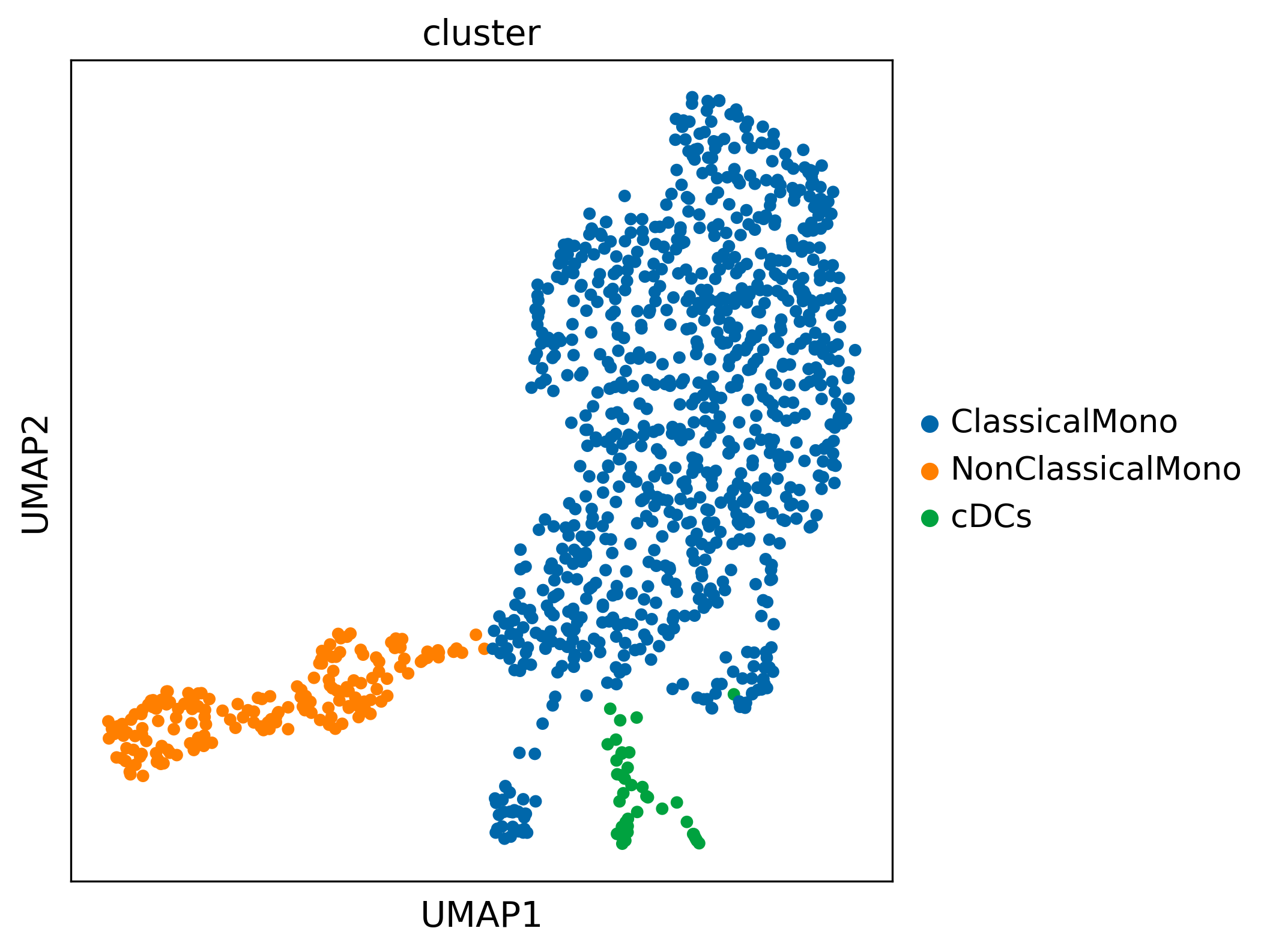

Supplement: Supplementary file 1 [file DataSheet_1.zip › Single-cell sequencing analysis/MPs/P22082602_rlabumap.png]

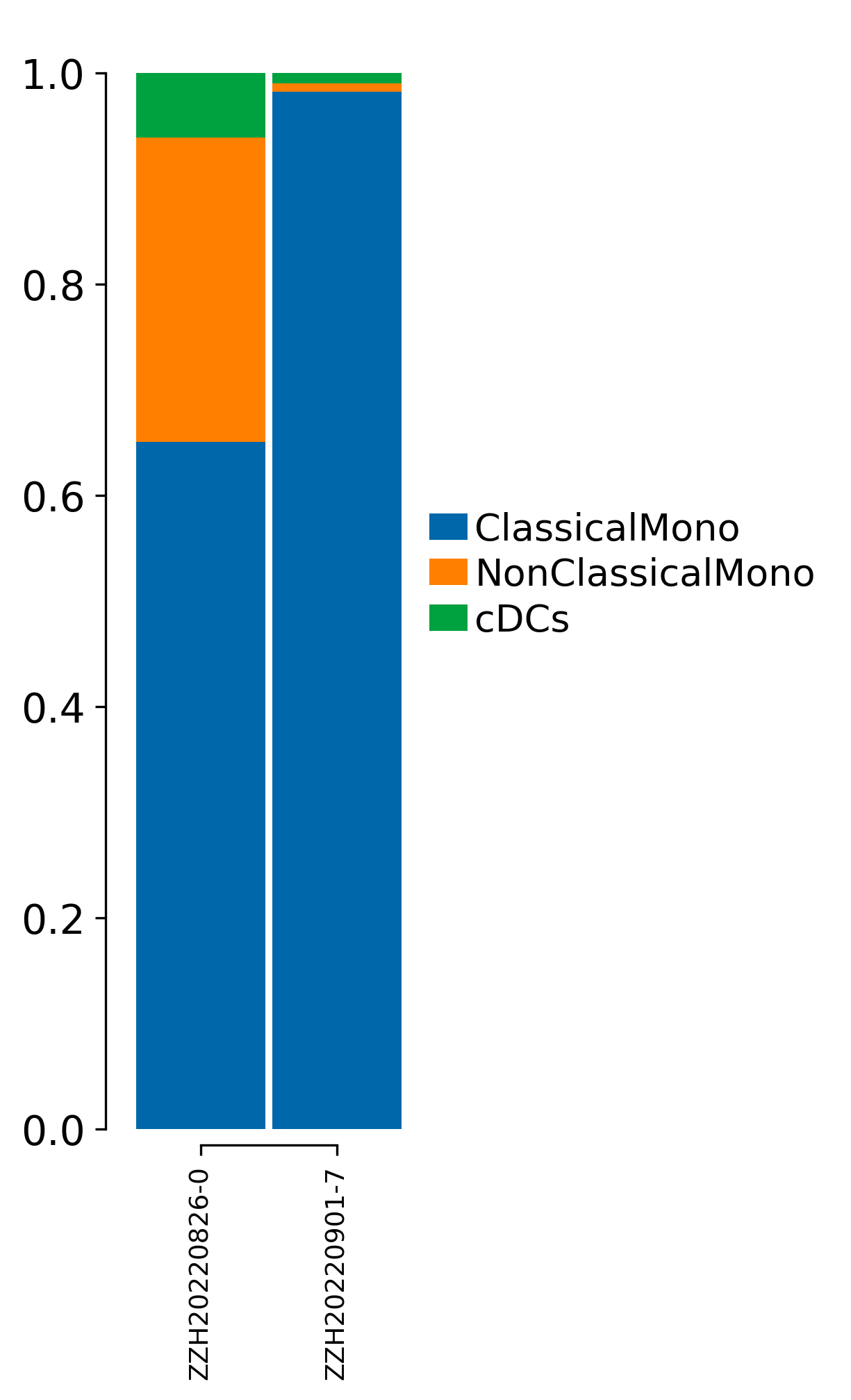

Supplement: Supplementary file 1 [file DataSheet_1.zip › Single-cell sequencing analysis/MPs/P22082602_sample_PercentPerCell.png]

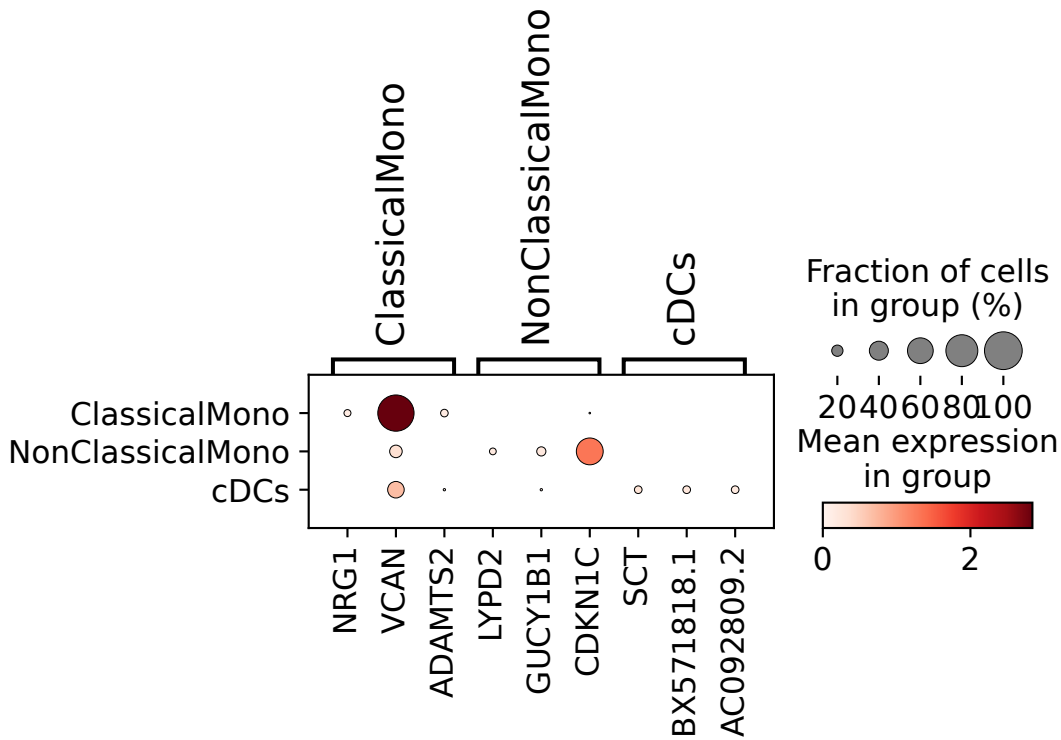

Supplement: Supplementary file 1 [file DataSheet_1.zip › Single-cell sequencing analysis/MPs/P22082602_TopMarkergenedotplot.pdf]

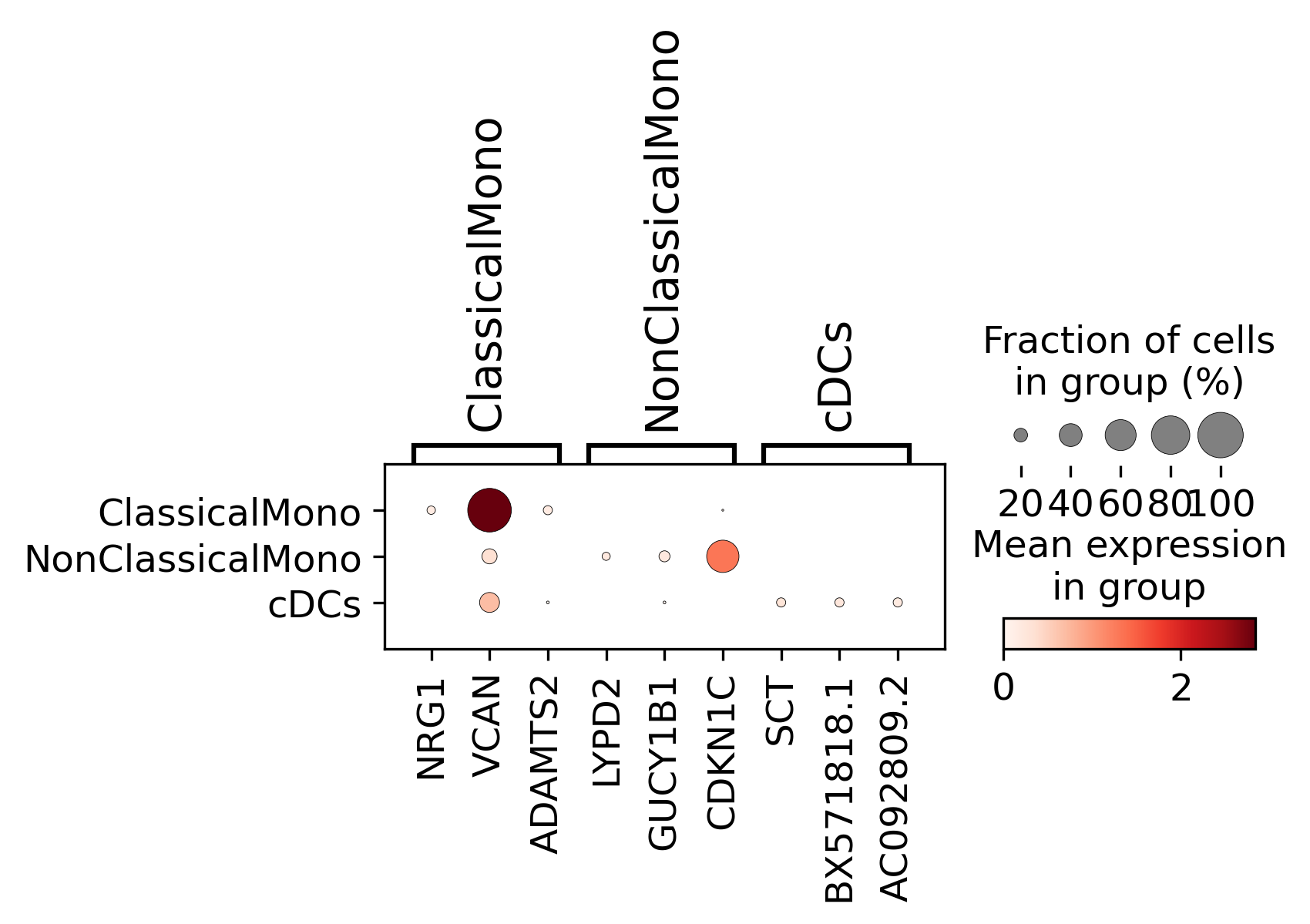

Supplement: Supplementary file 1 [file DataSheet_1.zip › Single-cell sequencing analysis/MPs/P22082602_TopMarkergenedotplot.png]

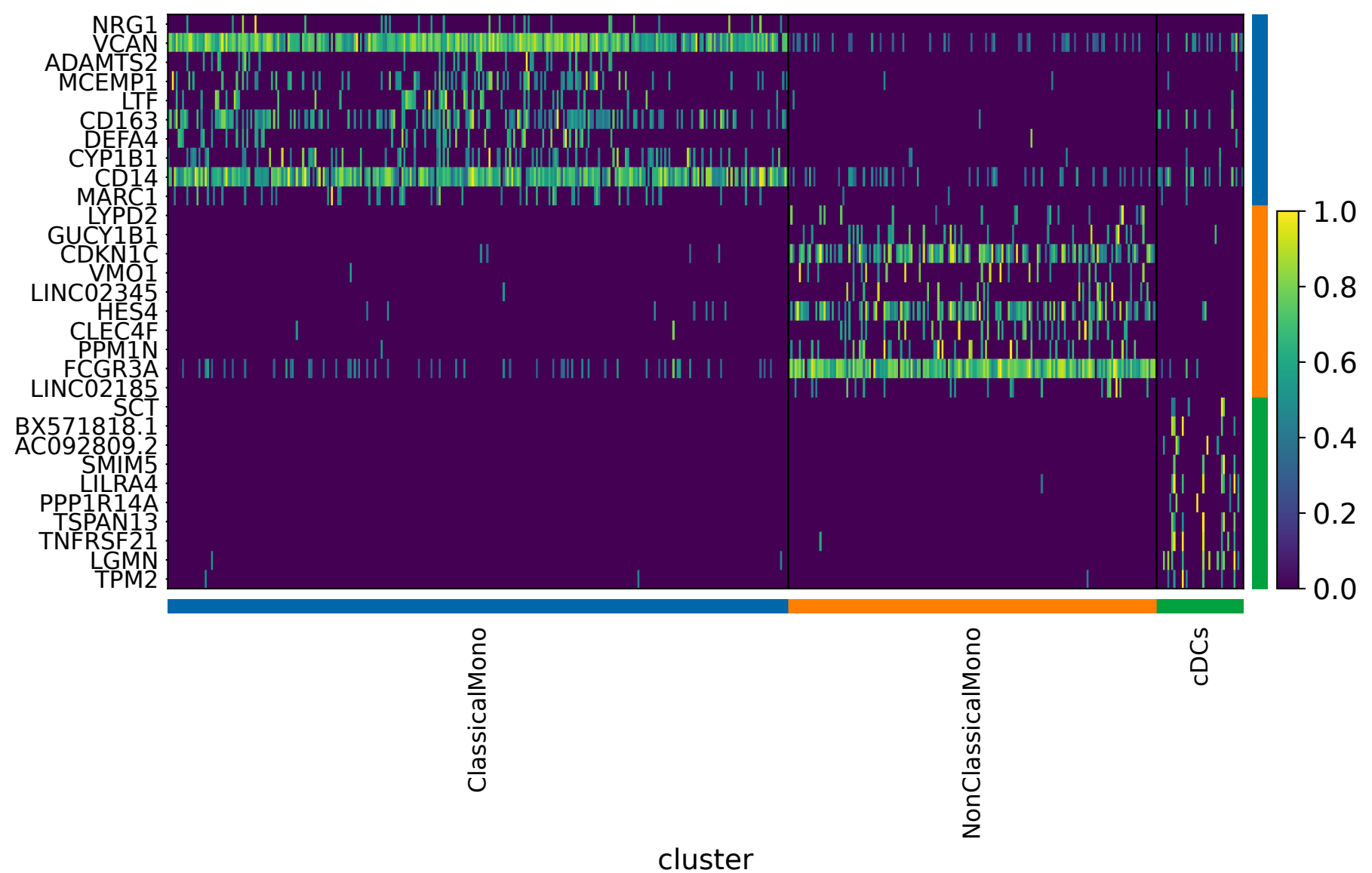

Supplement: Supplementary file 1 [file DataSheet_1.zip › Single-cell sequencing analysis/MPs/P22082602_TopMarkergeneHeatmap.pdf]

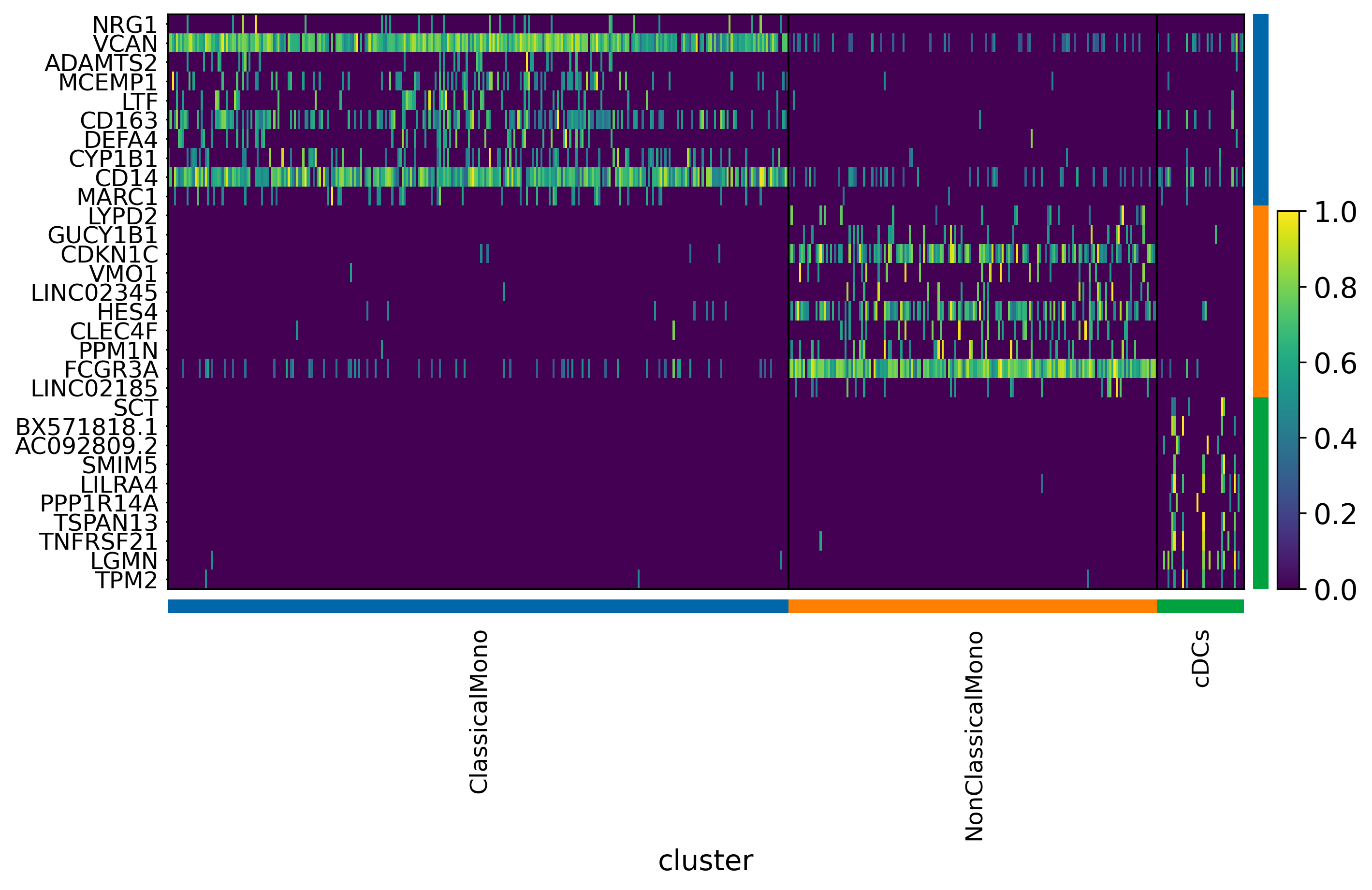

Supplement: Supplementary file 1 [file DataSheet_1.zip › Single-cell sequencing analysis/MPs/P22082602_TopMarkergeneHeatmap.png]

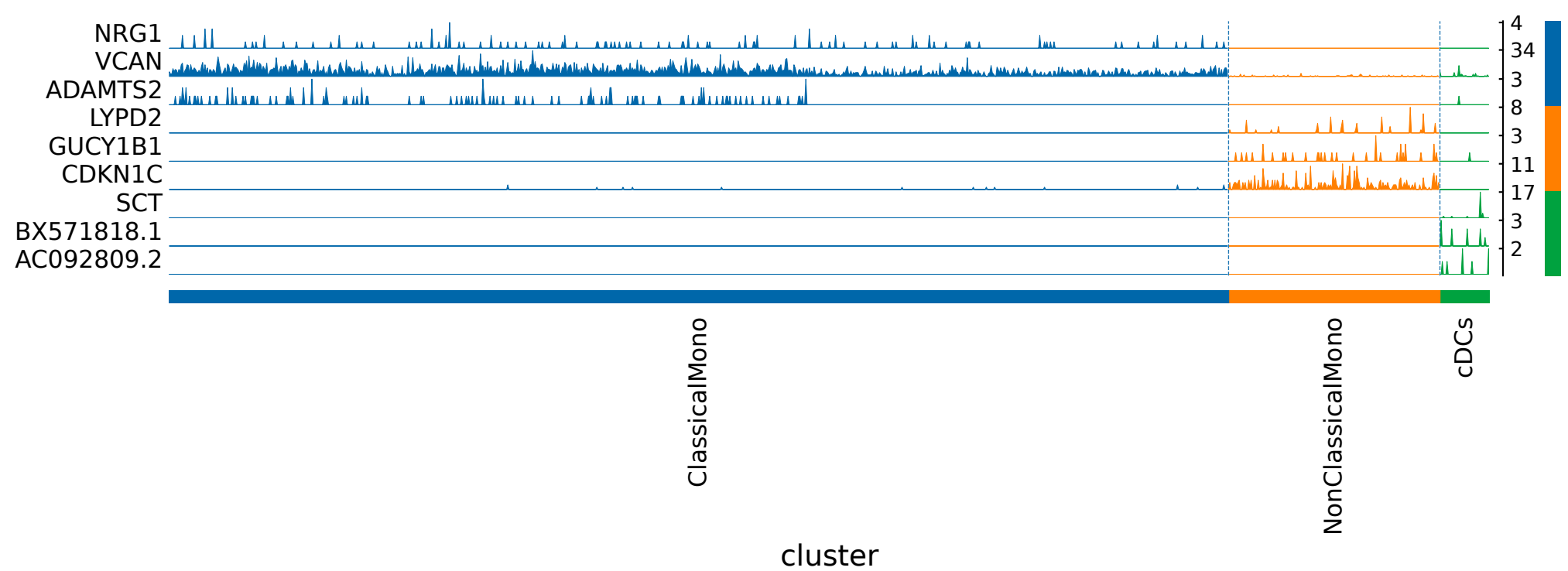

Supplement: Supplementary file 1 [file DataSheet_1.zip › Single-cell sequencing analysis/MPs/P22082602_TopMarkergeneTracksplot.pdf]

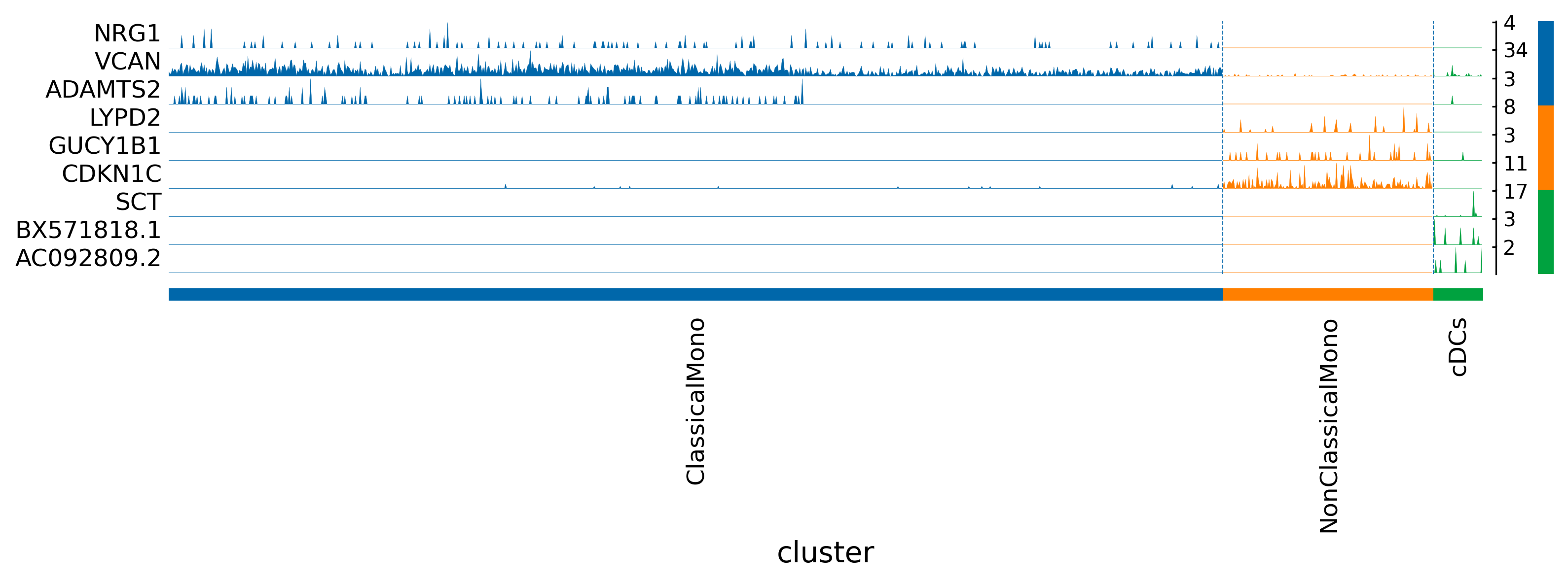

Supplement: Supplementary file 1 [file DataSheet_1.zip › Single-cell sequencing analysis/MPs/P22082602_TopMarkergeneTracksplot.png]

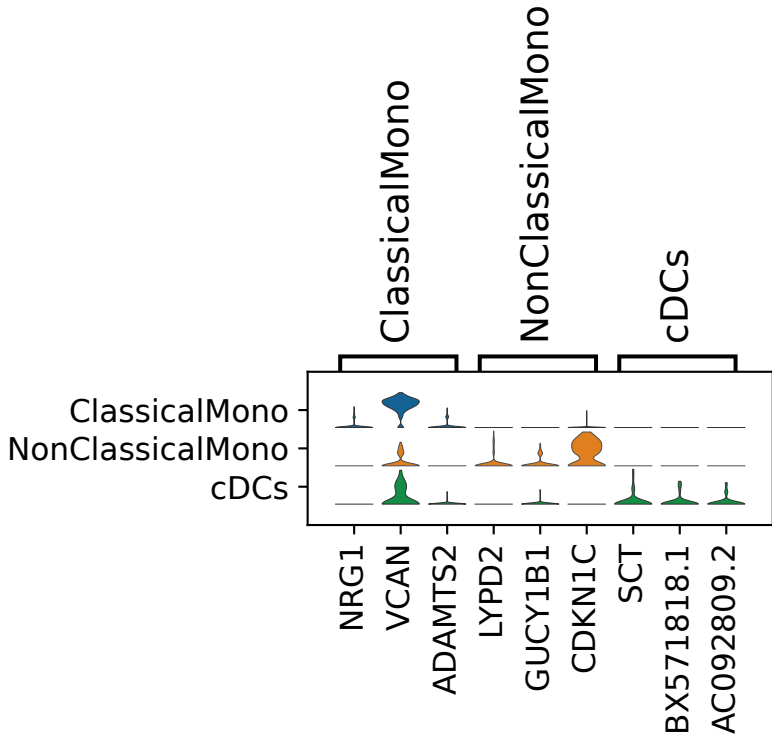

Supplement: Supplementary file 1 [file DataSheet_1.zip › Single-cell sequencing analysis/MPs/P22082602_TopStackedViolin.pdf]

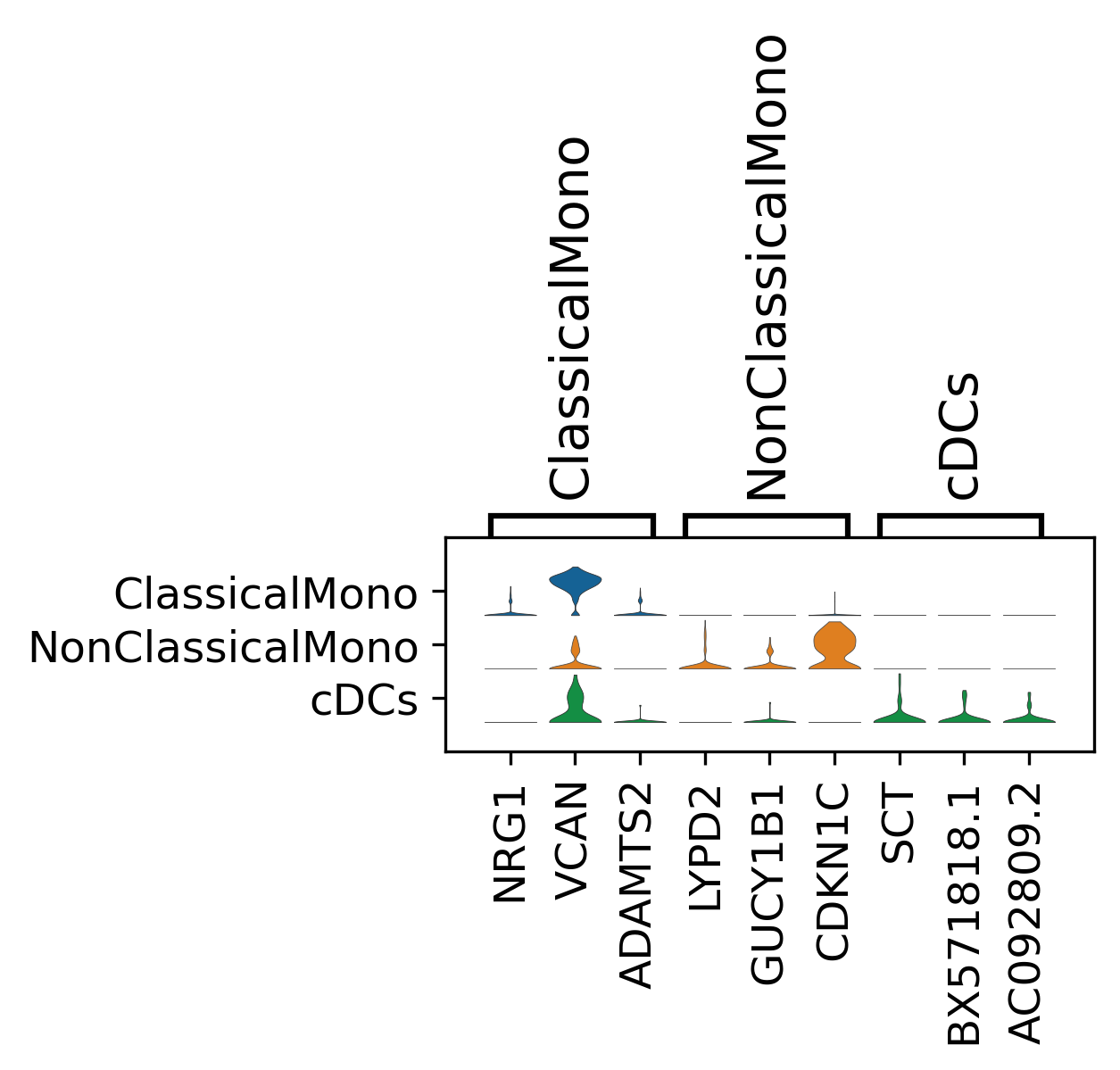

Supplement: Supplementary file 1 [file DataSheet_1.zip › Single-cell sequencing analysis/MPs/P22082602_TopStackedViolin.png]

gname

UMAP2

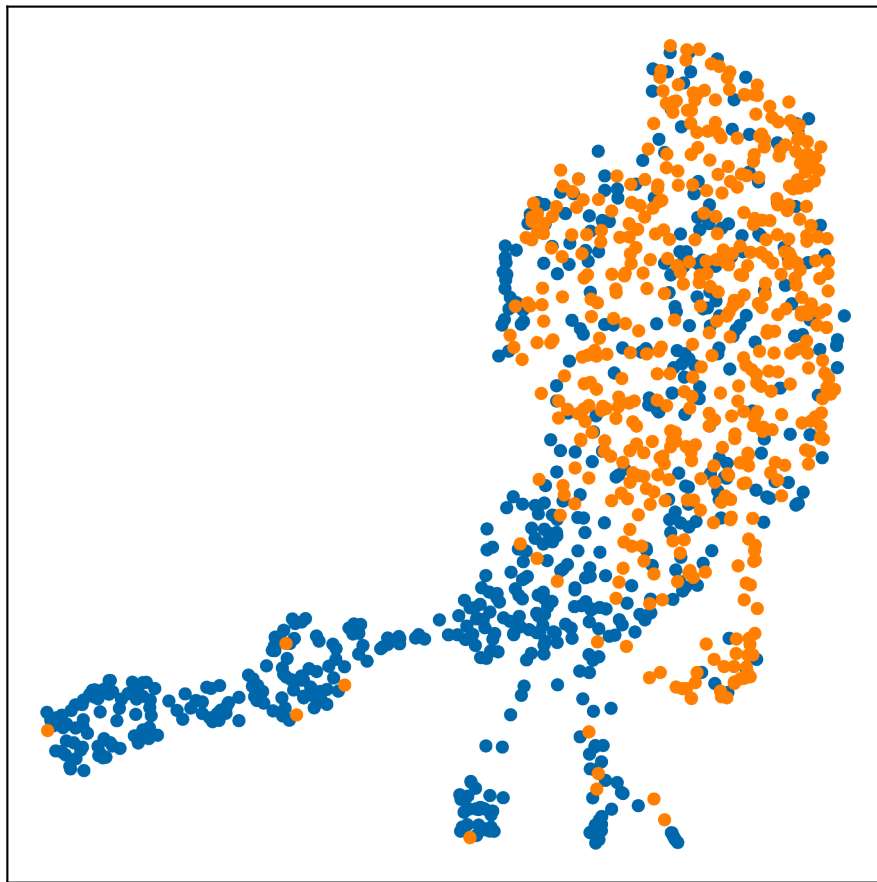

● ZZH20220826-0

● ZZH20220901-7

UMAP1

Supplement: Supplementary file 1 [file DataSheet_1.zip › Single-cell sequencing analysis/MPs/P22082602_umap_groups.pdf]

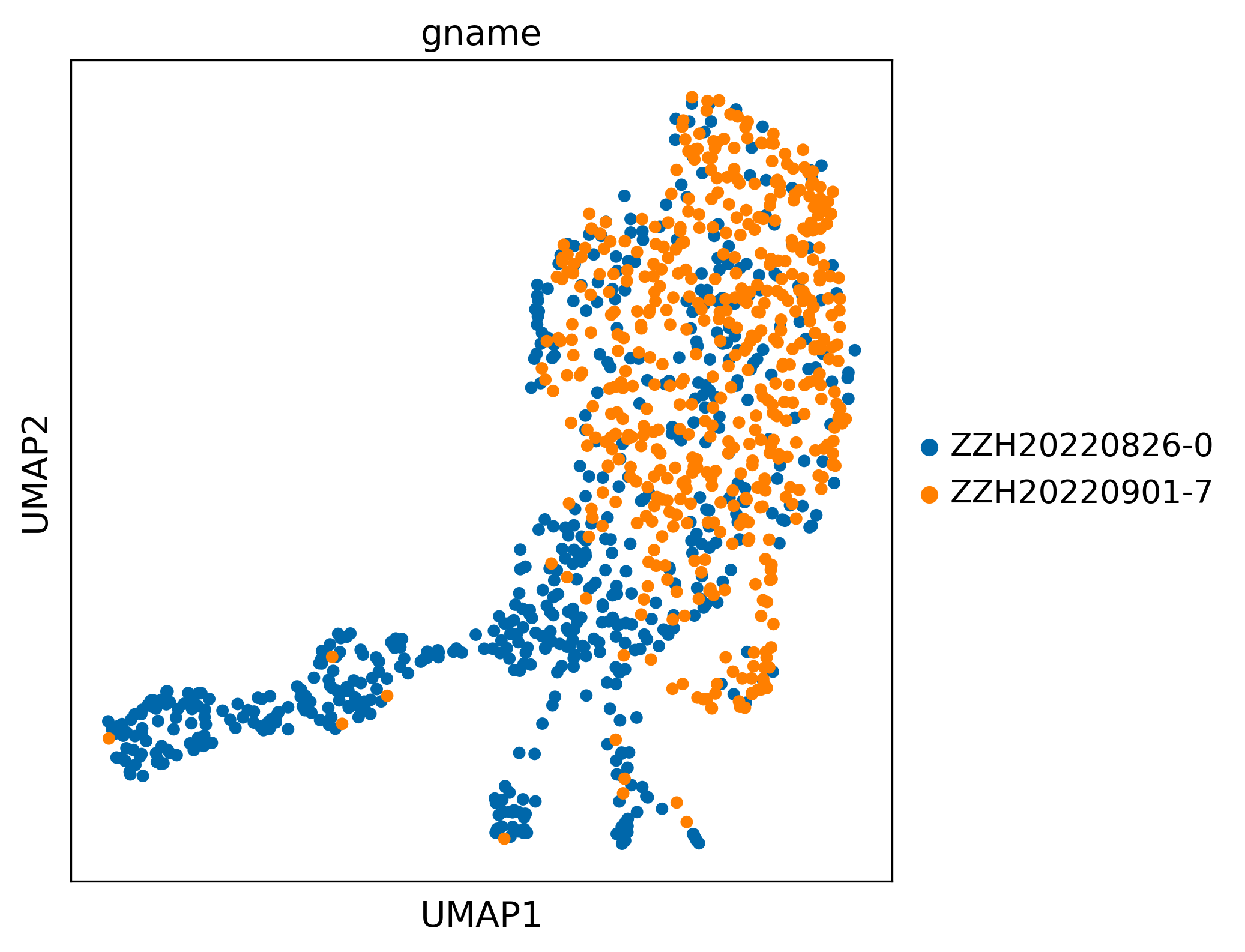

Supplement: Supplementary file 1 [file DataSheet_1.zip › Single-cell sequencing analysis/MPs/P22082602_umap_groups.png]

sample

UMAP2

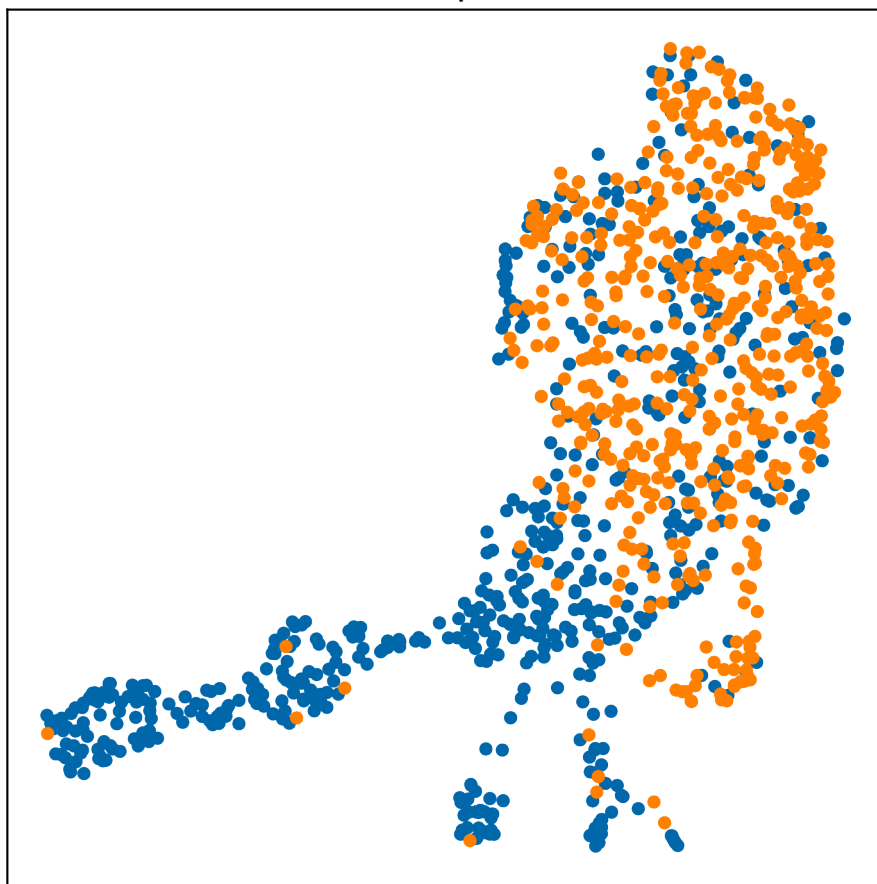

● ZZH20220826-0

● ZZH20220901-7

UMAP1

Supplement: Supplementary file 1 [file DataSheet_1.zip › Single-cell sequencing analysis/MPs/P22082602_umap_samples.pdf]

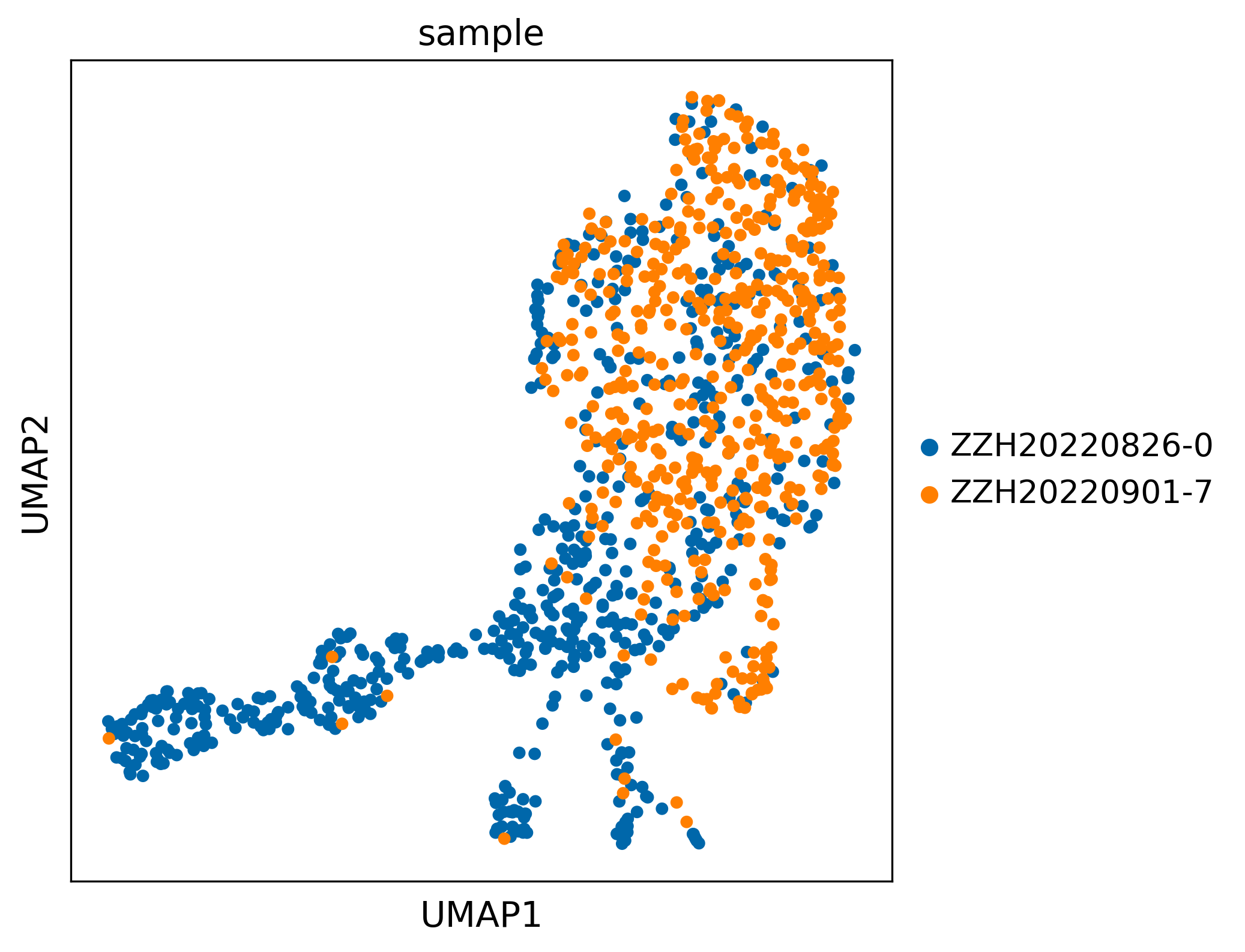

Supplement: Supplementary file 1 [file DataSheet_1.zip › Single-cell sequencing analysis/MPs/P22082602_umap_samples.png]

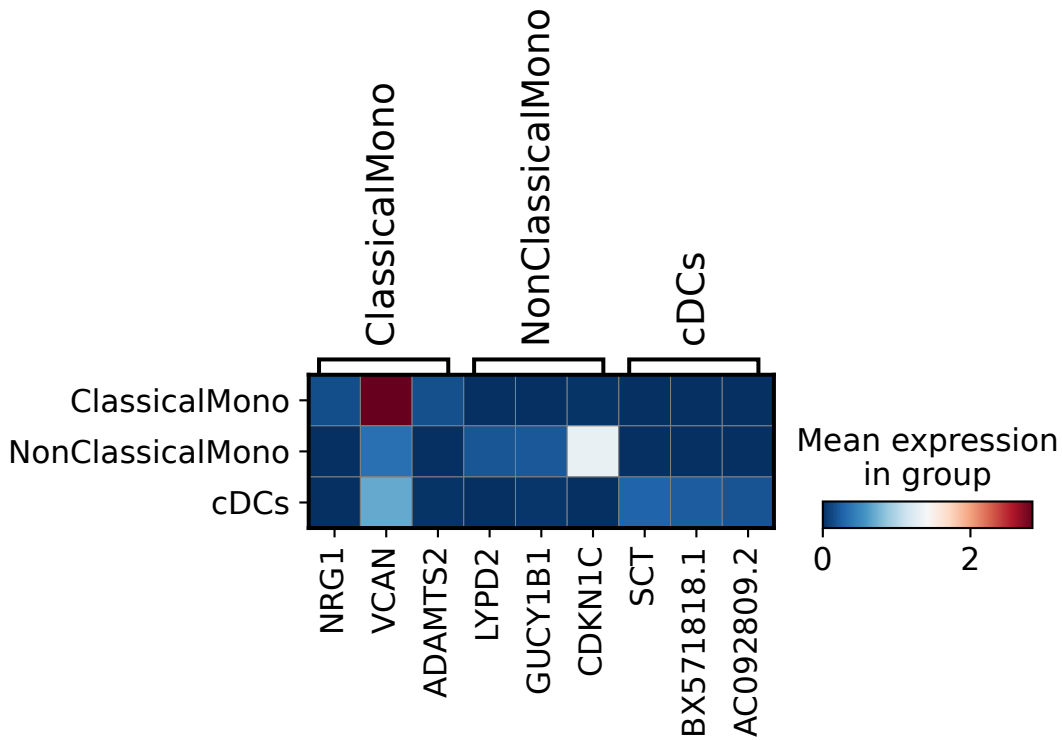

Supplement: Supplementary file 1 [file DataSheet_1.zip › Single-cell sequencing analysis/MPs/P22082602_Zscore_matrixplot.pdf]

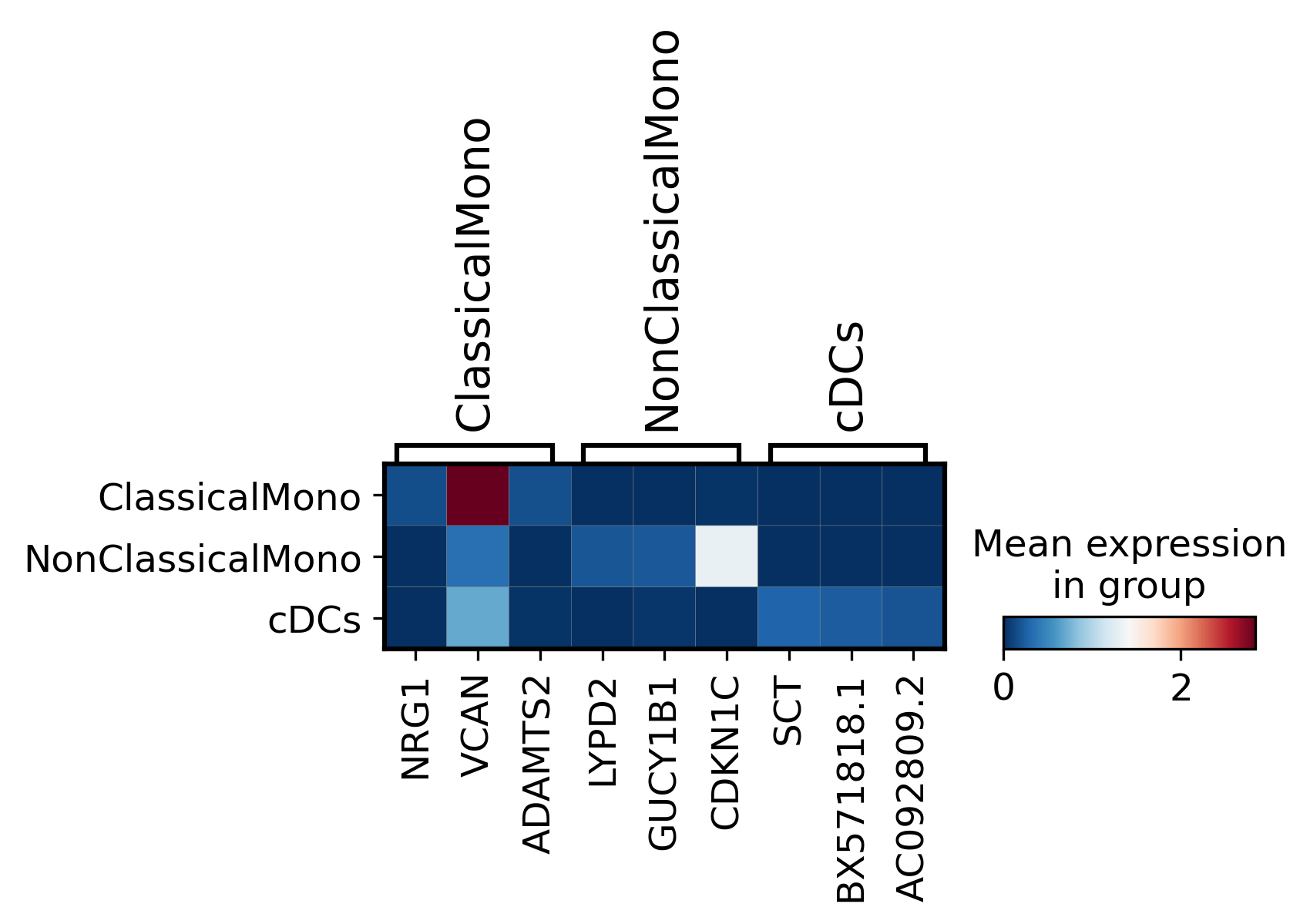

Supplement: Supplementary file 1 [file DataSheet_1.zip › Single-cell sequencing analysis/MPs/P22082602_Zscore_matrixplot.png]

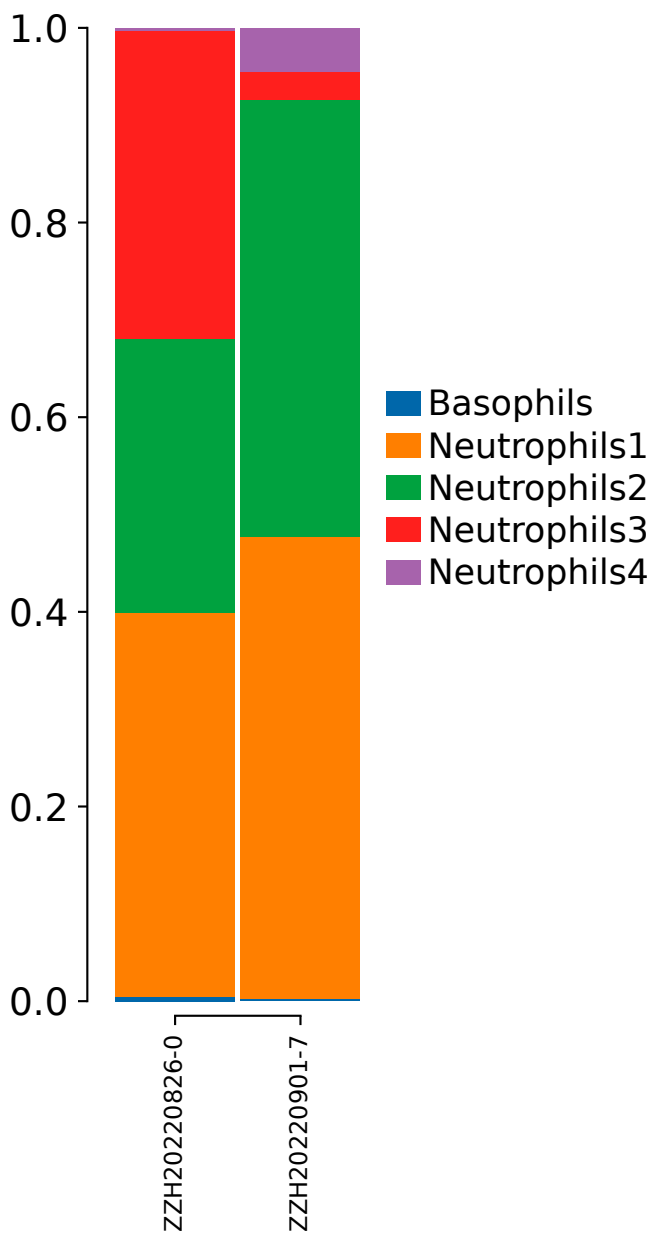

Supplement: Supplementary file 1 [file DataSheet_1.zip › Single-cell sequencing analysis/Neutrophils/P22082602_group_PercentPerCell.pdf]

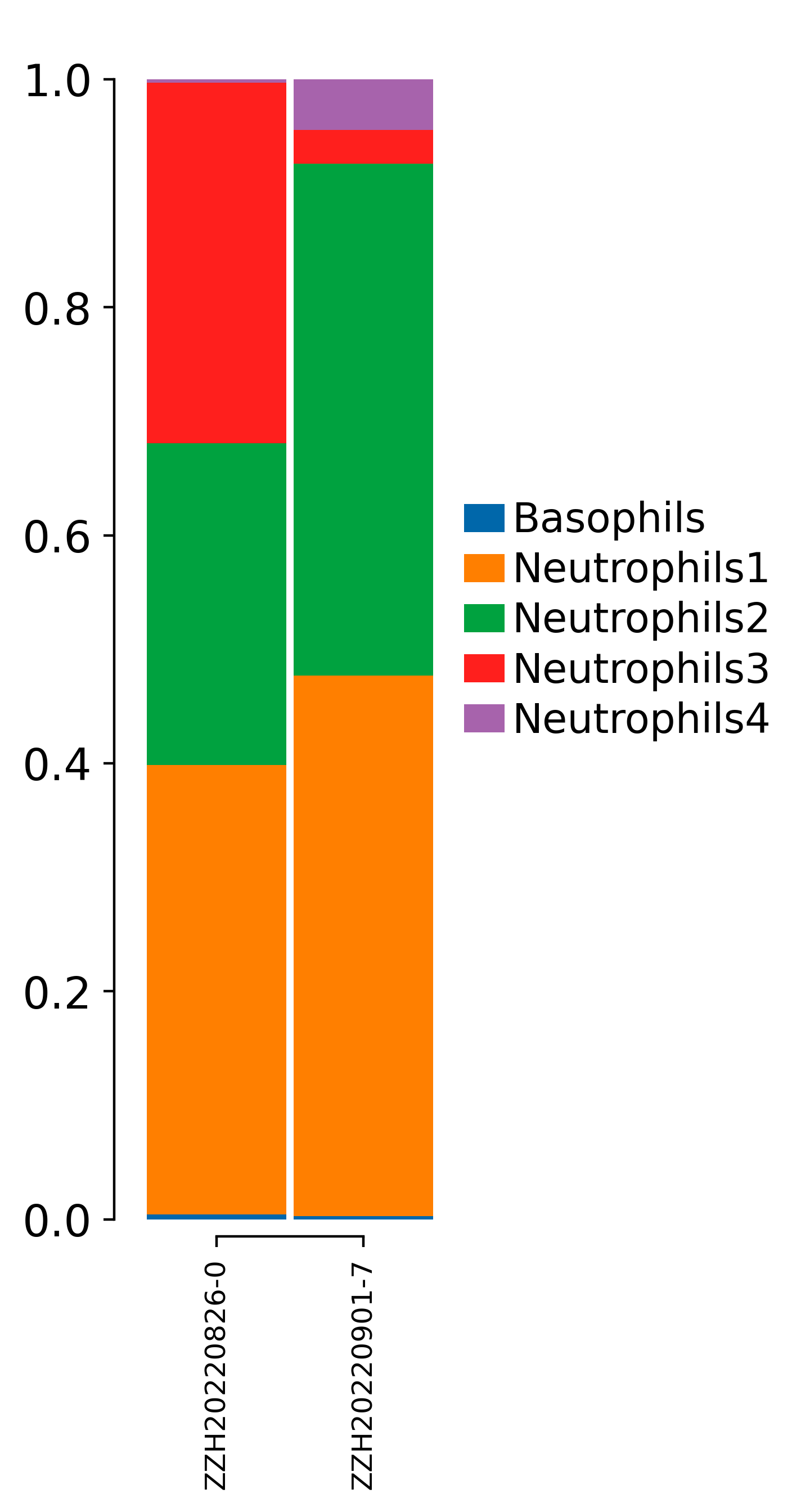

Supplement: Supplementary file 1 [file DataSheet_1.zip › Single-cell sequencing analysis/Neutrophils/P22082602_group_PercentPerCell.png]

cluster

UMAP2

**Neutrophils4**

**Neutrophils1**

**Neutrophils3**

**Neutrophils2**

**Basophils**

UMAP1

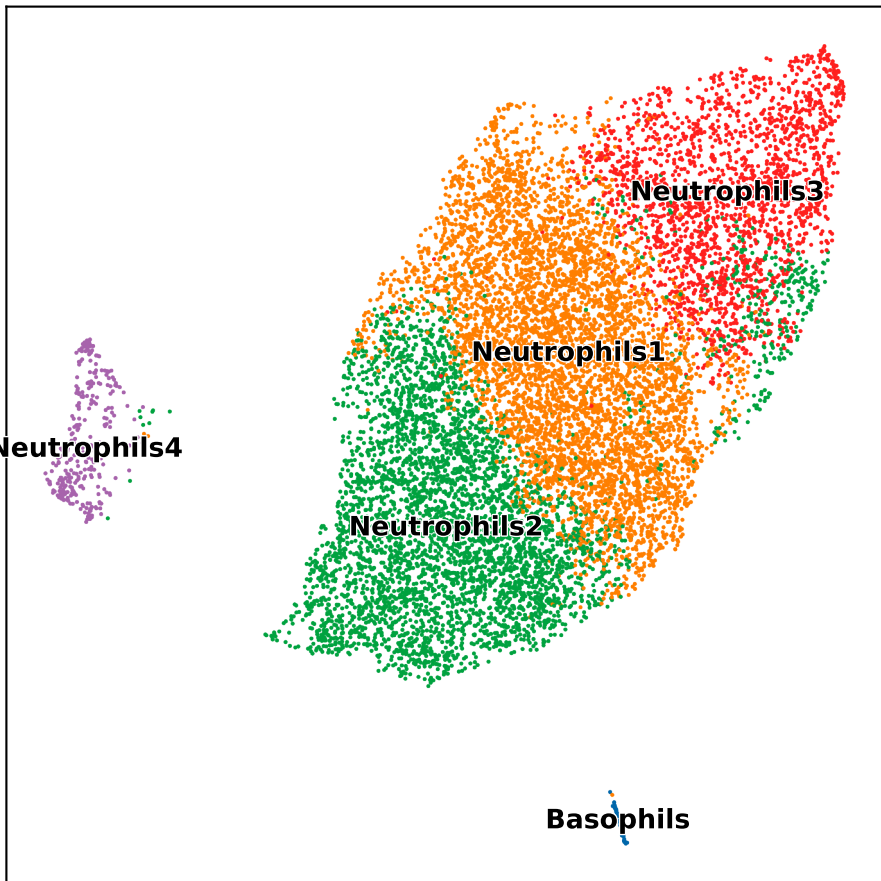

Supplement: Supplementary file 1 [file DataSheet_1.zip › Single-cell sequencing analysis/Neutrophils/P22082602_labumap.pdf]

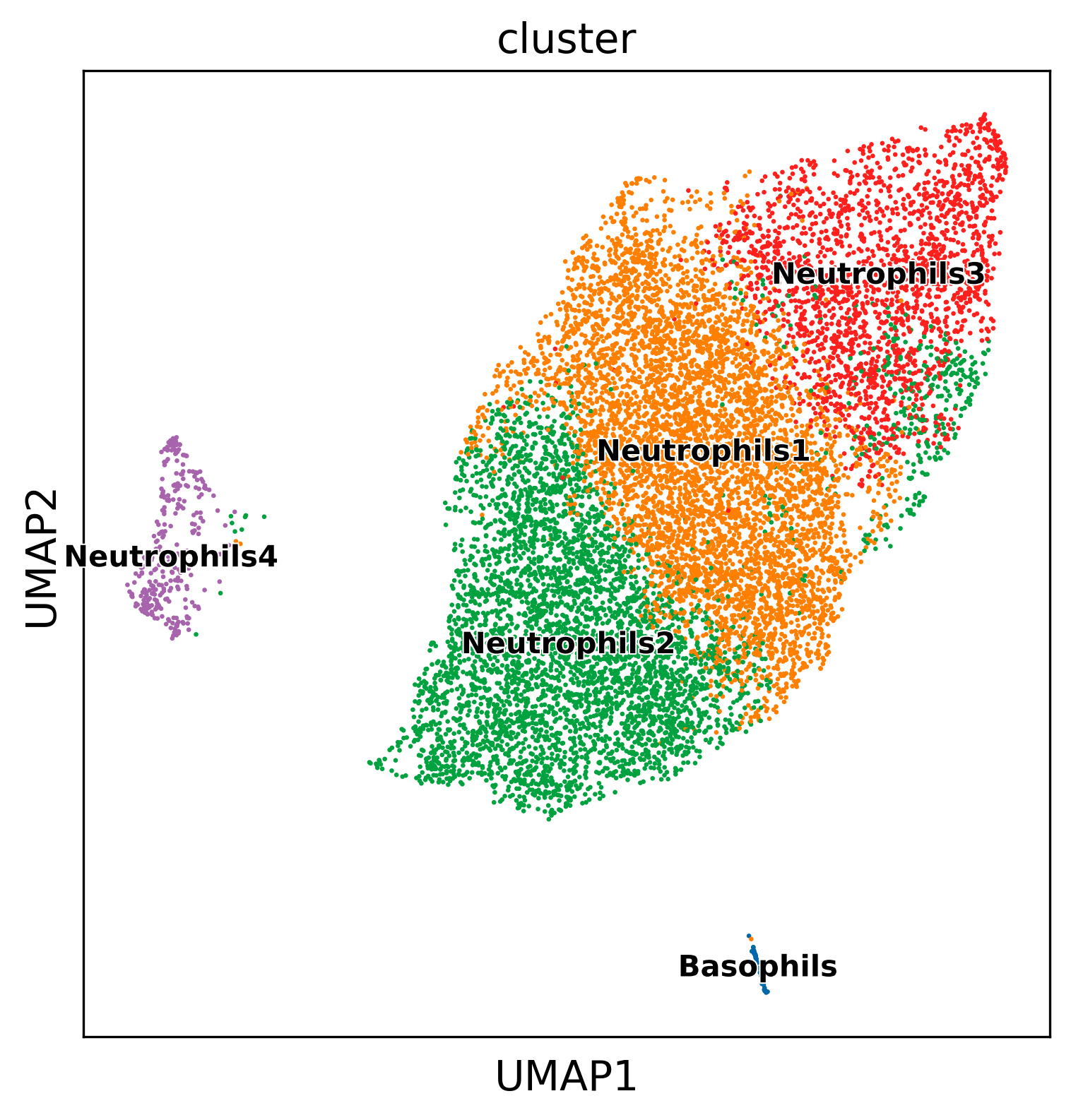

Supplement: Supplementary file 1 [file DataSheet_1.zip › Single-cell sequencing analysis/Neutrophils/P22082602_labumap.png]

cluster

UMAP2

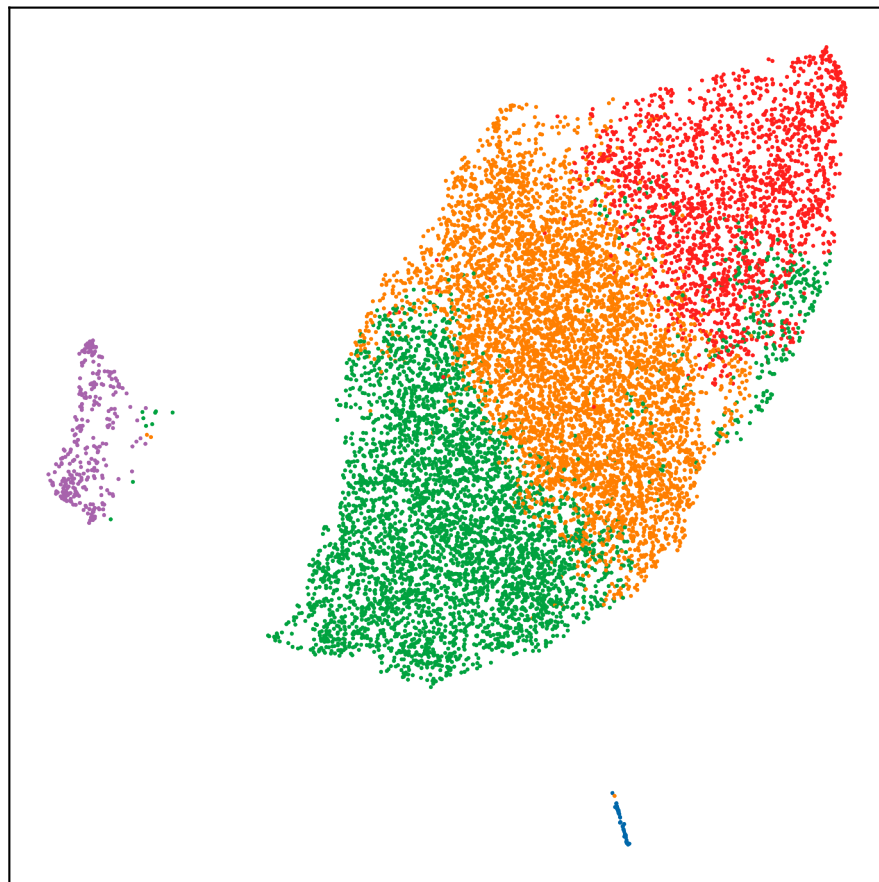

UMAP1

- Basophils
- Neutrophils1
- Neutrophils2
- Neutrophils3
- Neutrophils4

Supplement: Supplementary file 1 [file DataSheet_1.zip › Single-cell sequencing analysis/Neutrophils/P22082602_rlabumap.pdf]

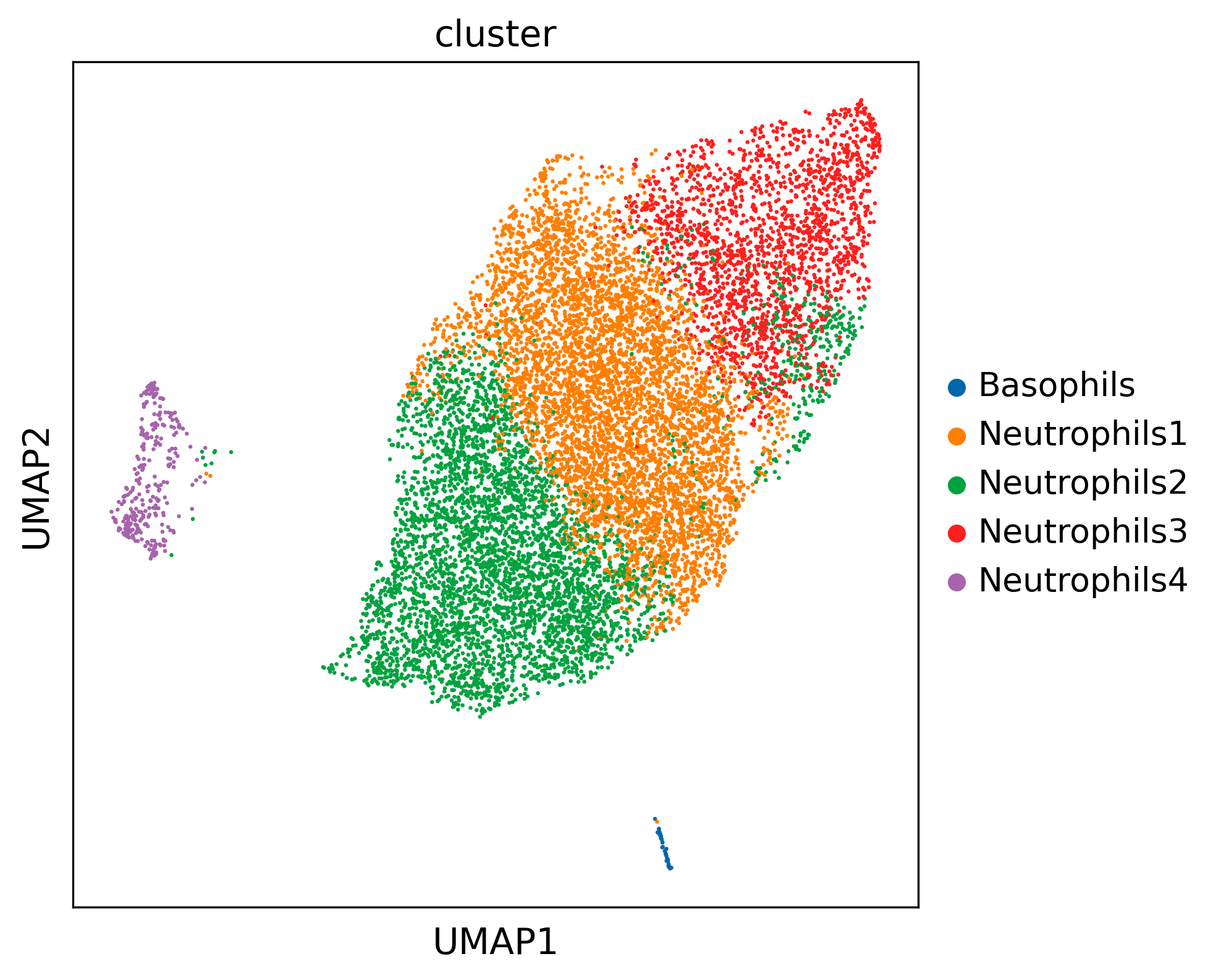

Supplement: Supplementary file 1 [file DataSheet_1.zip › Single-cell sequencing analysis/Neutrophils/P22082602_rlabumap.png]

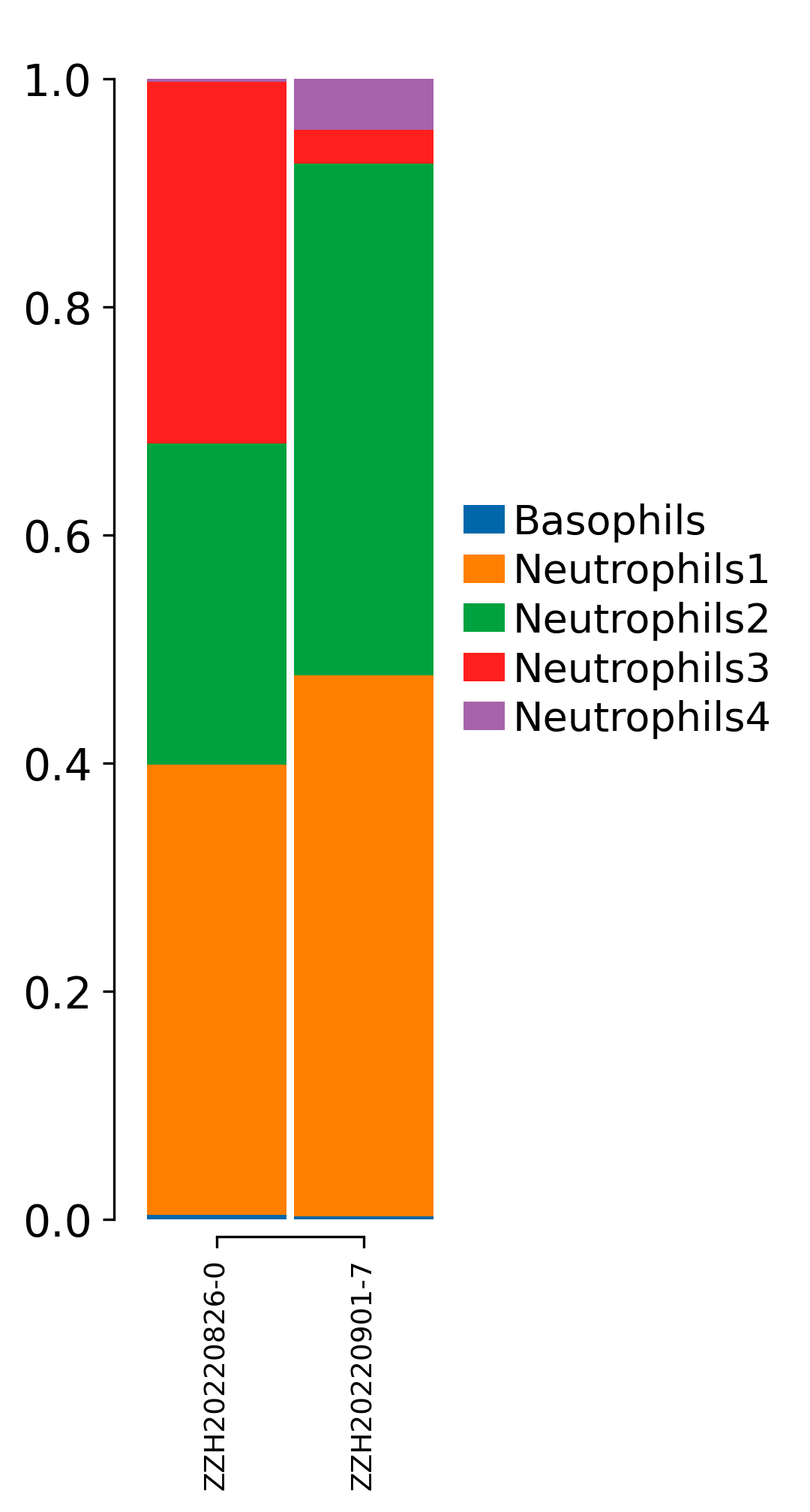

Supplement: Supplementary file 1 [file DataSheet_1.zip › Single-cell sequencing analysis/Neutrophils/P22082602_sample_PercentPerCell.png]

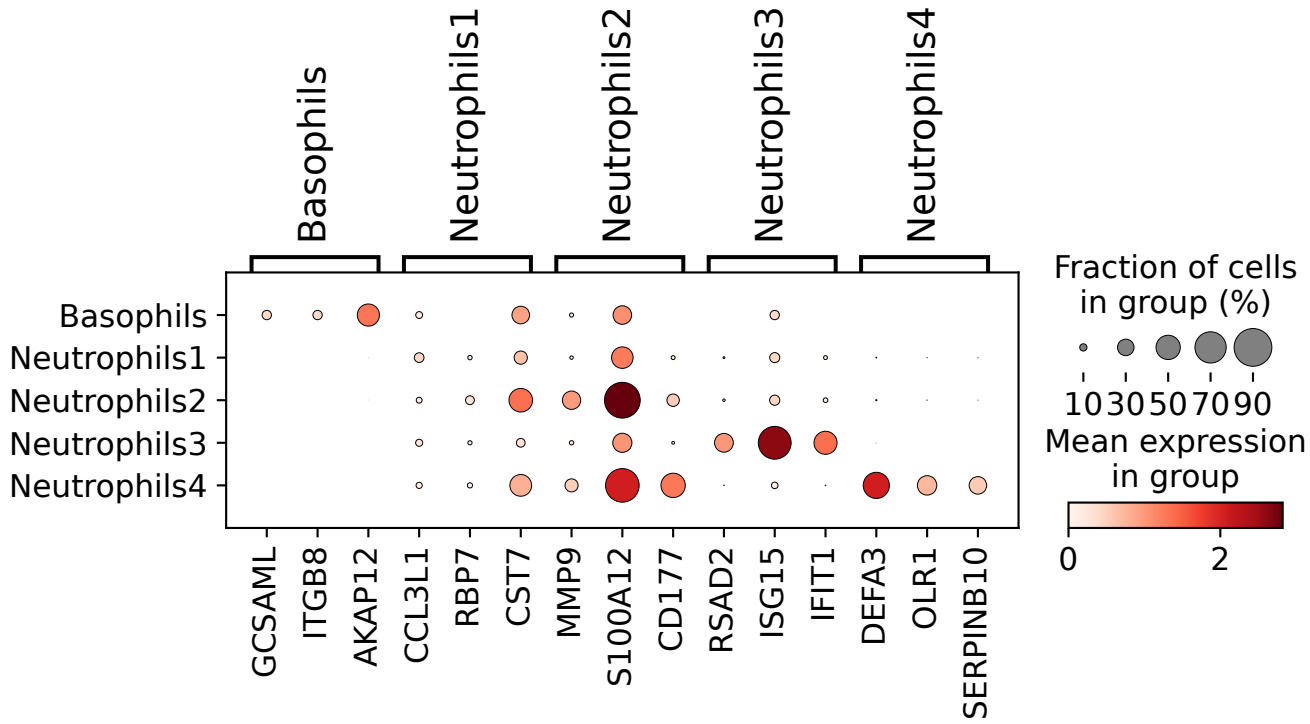

Supplement: Supplementary file 1 [file DataSheet_1.zip › Single-cell sequencing analysis/Neutrophils/P22082602_TopMarkergenedotplot.pdf]

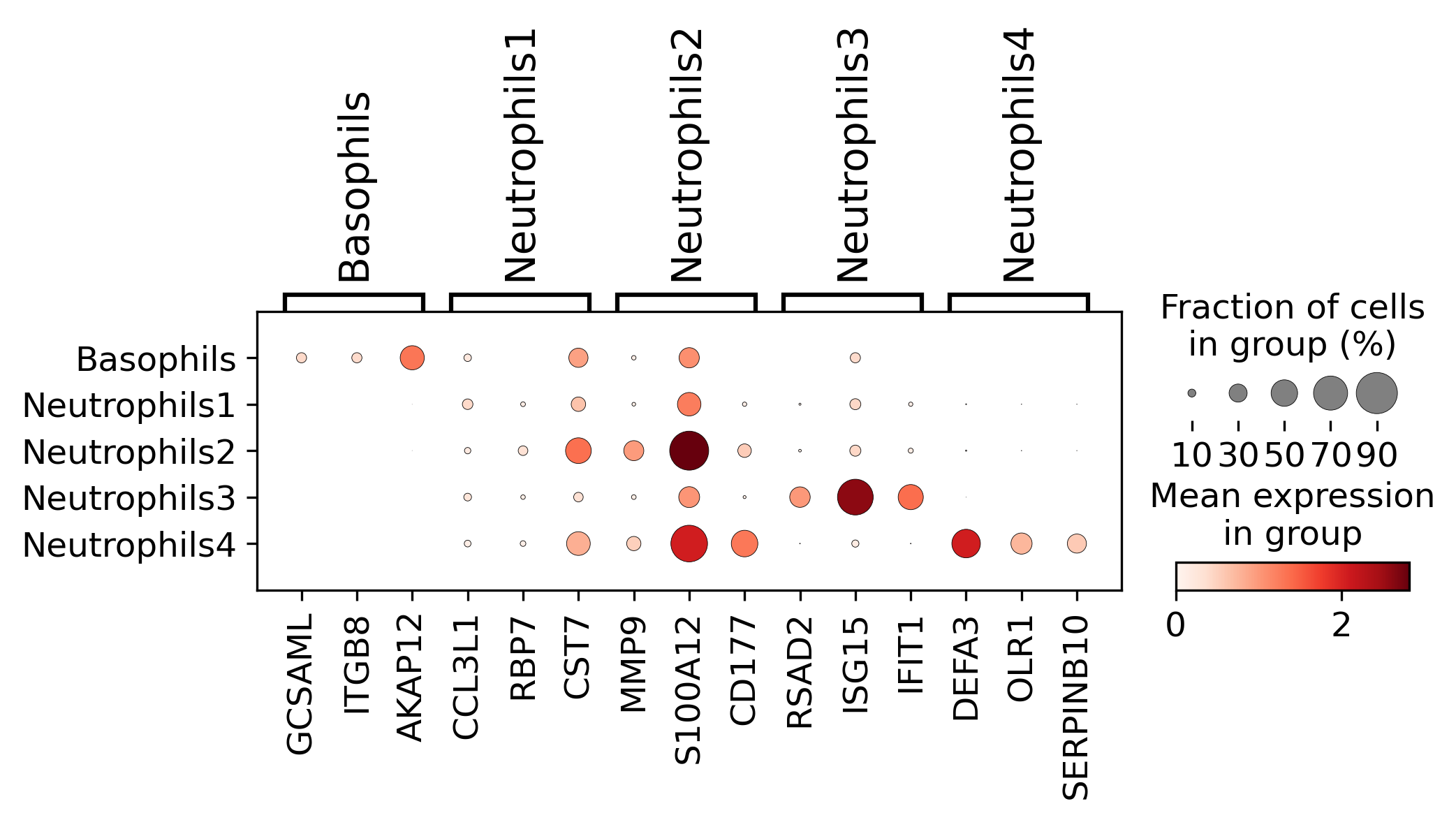

Supplement: Supplementary file 1 [file DataSheet_1.zip › Single-cell sequencing analysis/Neutrophils/P22082602_TopMarkergenedotplot.png]

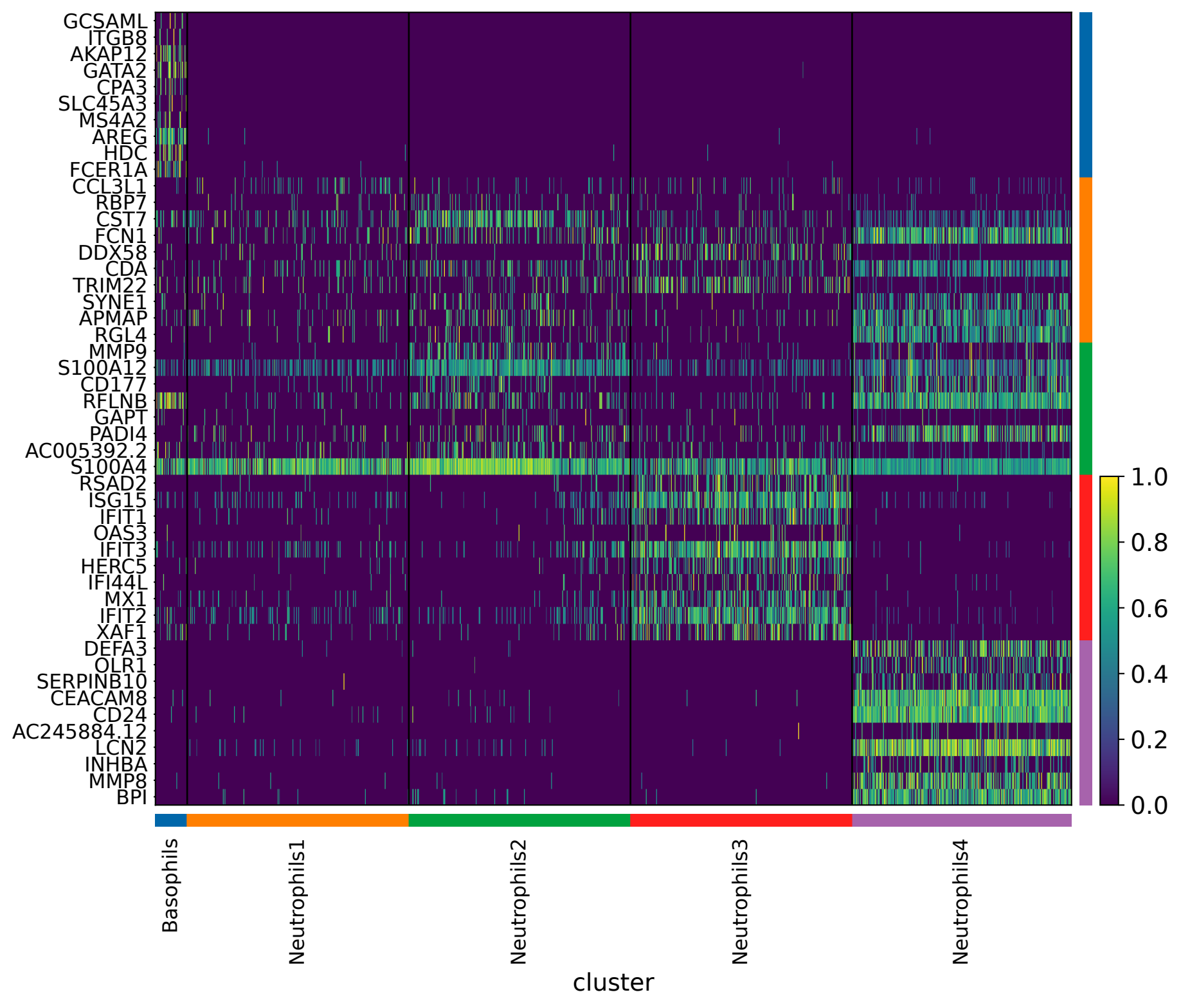

Supplement: Supplementary file 1 [file DataSheet_1.zip › Single-cell sequencing analysis/Neutrophils/P22082602_TopMarkergeneHeatmap.pdf]

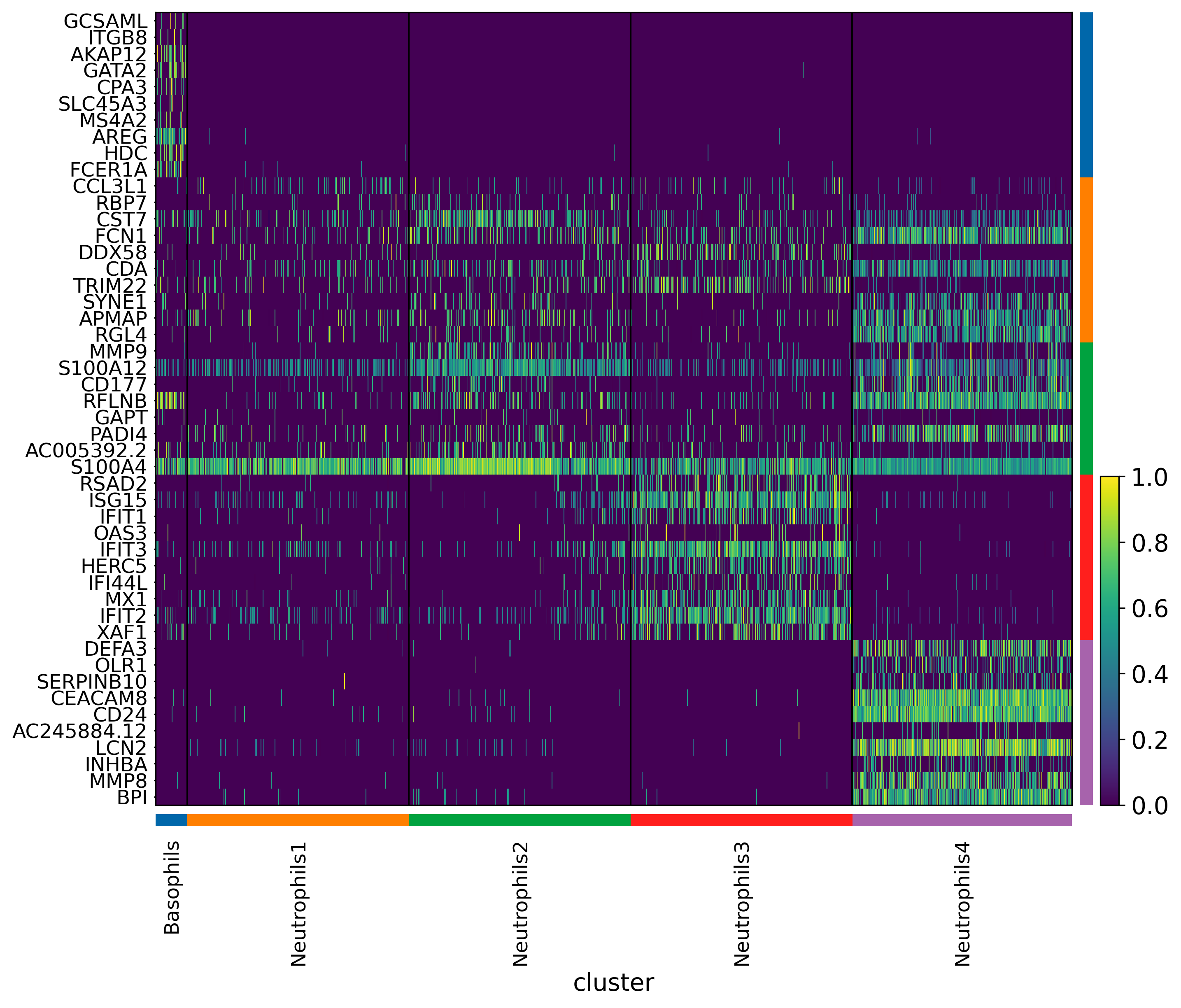

Supplement: Supplementary file 1 [file DataSheet_1.zip › Single-cell sequencing analysis/Neutrophils/P22082602_TopMarkergeneHeatmap.png]

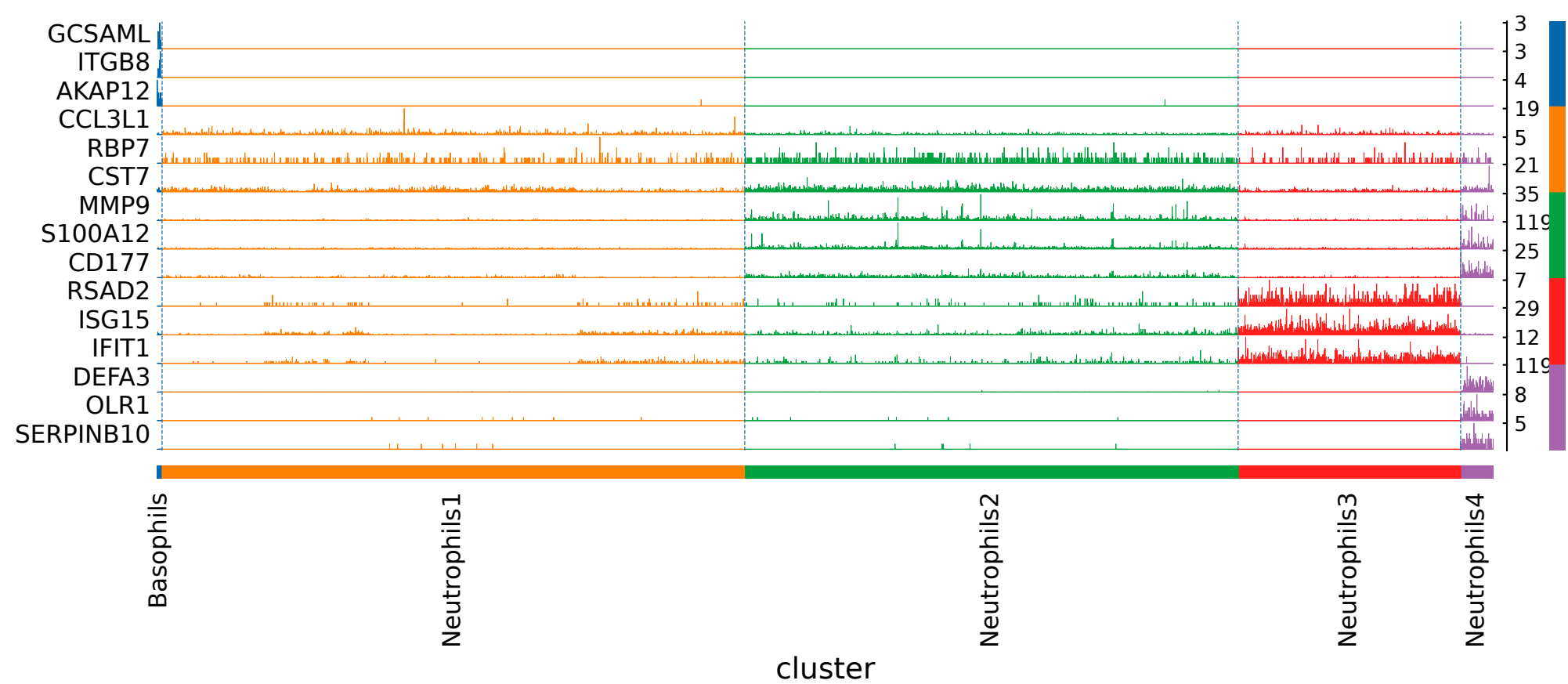

Supplement: Supplementary file 1 [file DataSheet_1.zip › Single-cell sequencing analysis/Neutrophils/P22082602_TopMarkergeneTracksplot.pdf]

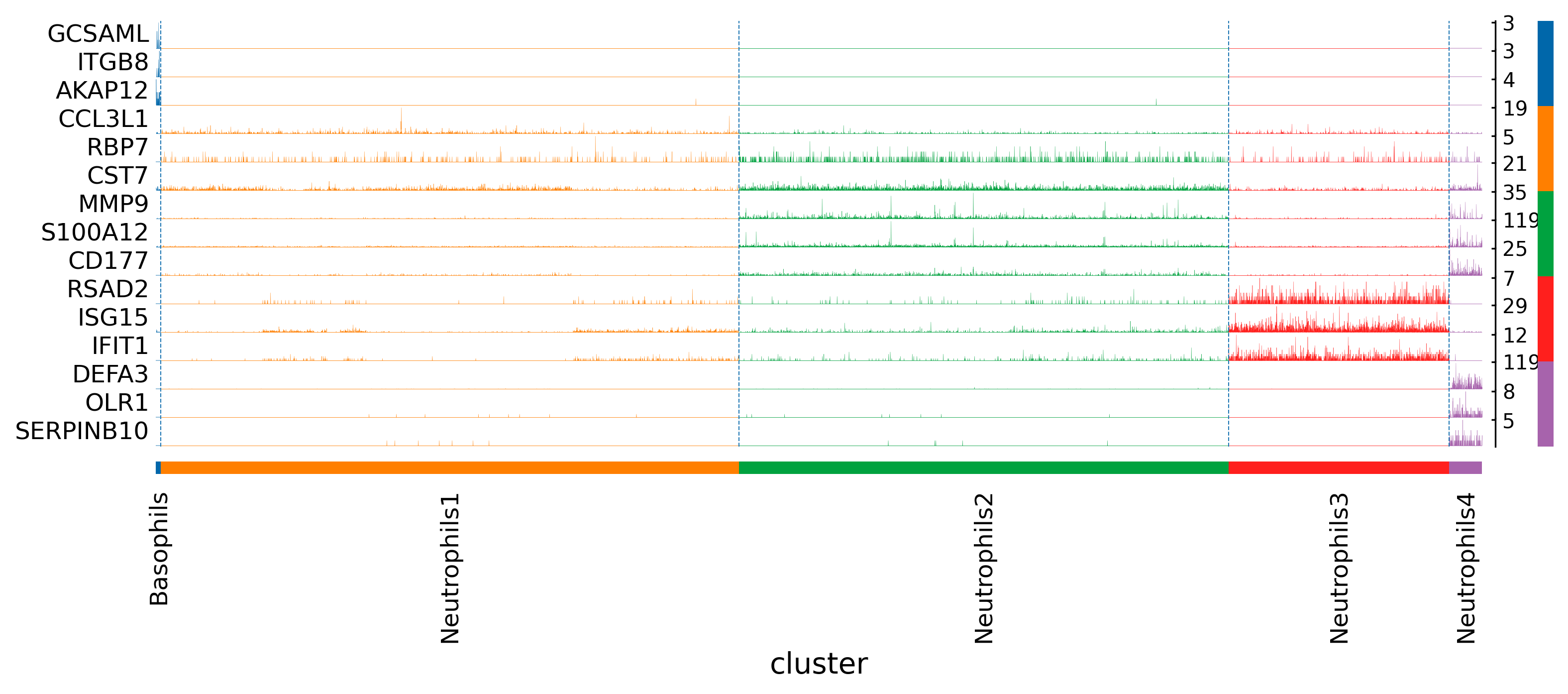

Supplement: Supplementary file 1 [file DataSheet_1.zip › Single-cell sequencing analysis/Neutrophils/P22082602_TopMarkergeneTracksplot.png]

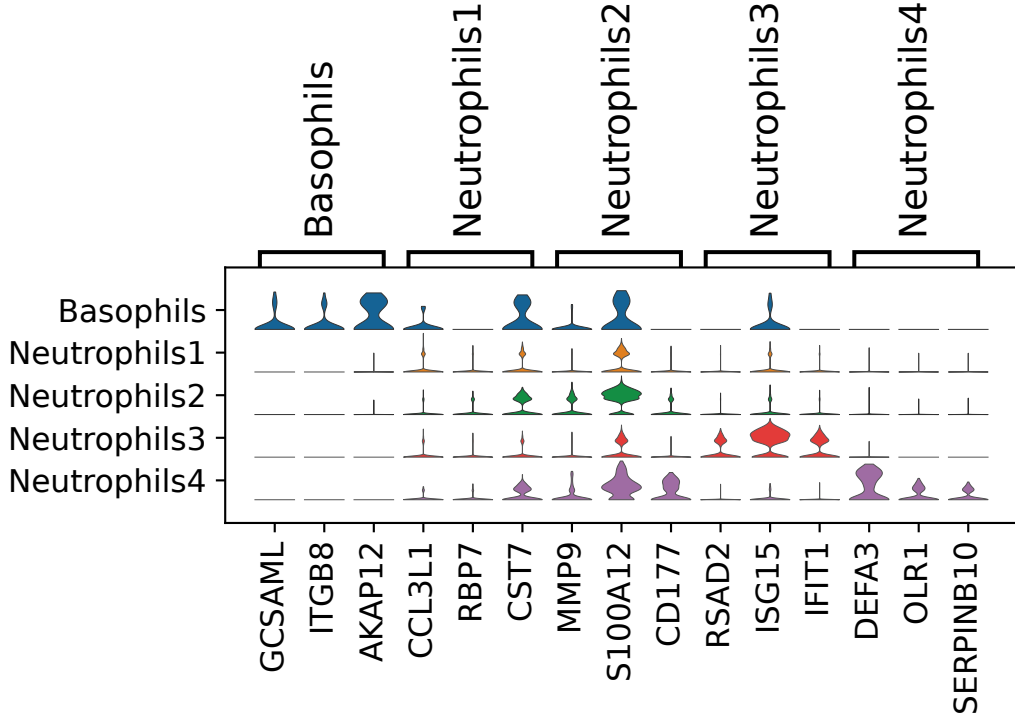

Supplement: Supplementary file 1 [file DataSheet_1.zip › Single-cell sequencing analysis/Neutrophils/P22082602_TopStackedViolin.pdf]

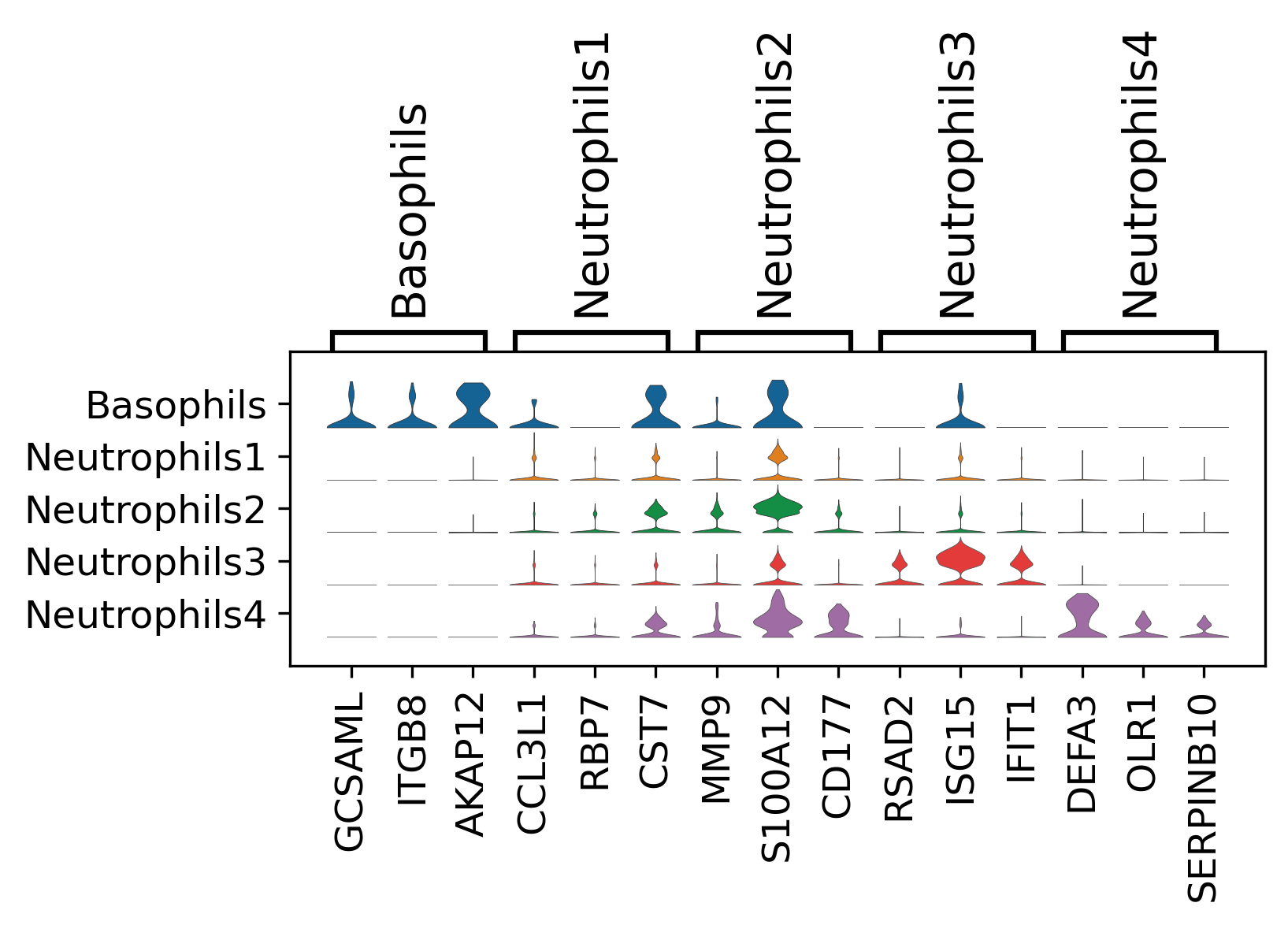

Supplement: Supplementary file 1 [file DataSheet_1.zip › Single-cell sequencing analysis/Neutrophils/P22082602_TopStackedViolin.png]

gname

UMAP2

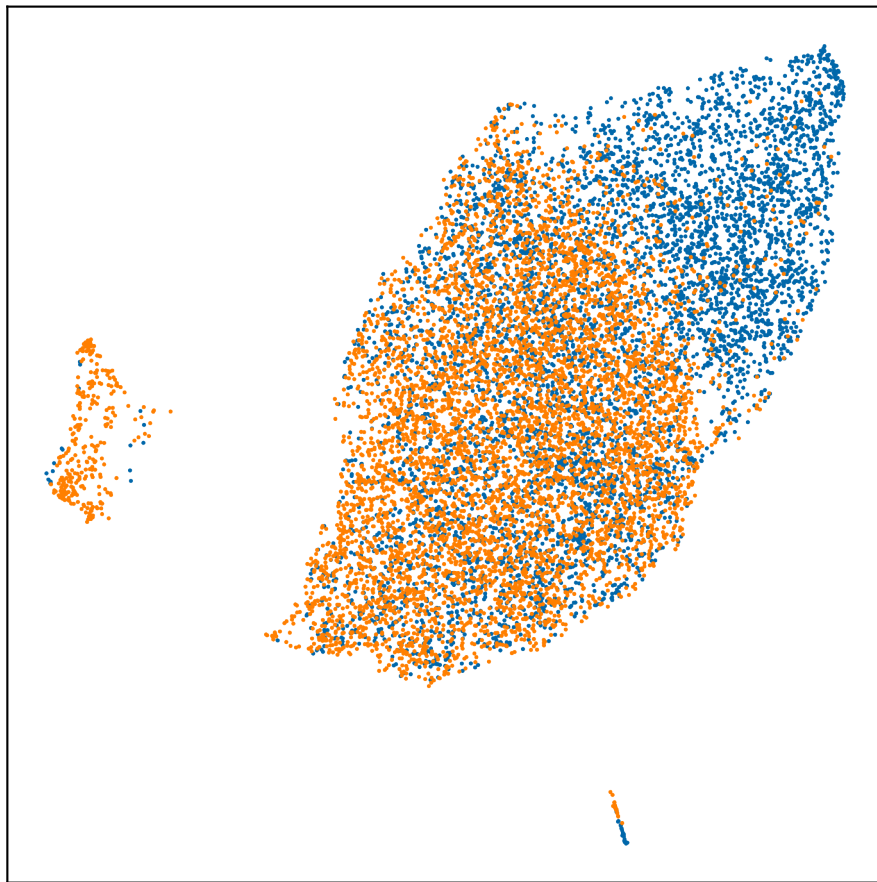

● ZZH20220826-0

● ZZH20220901-7

UMAP1

Supplement: Supplementary file 1 [file DataSheet_1.zip › Single-cell sequencing analysis/Neutrophils/P22082602_umap_groups.pdf]

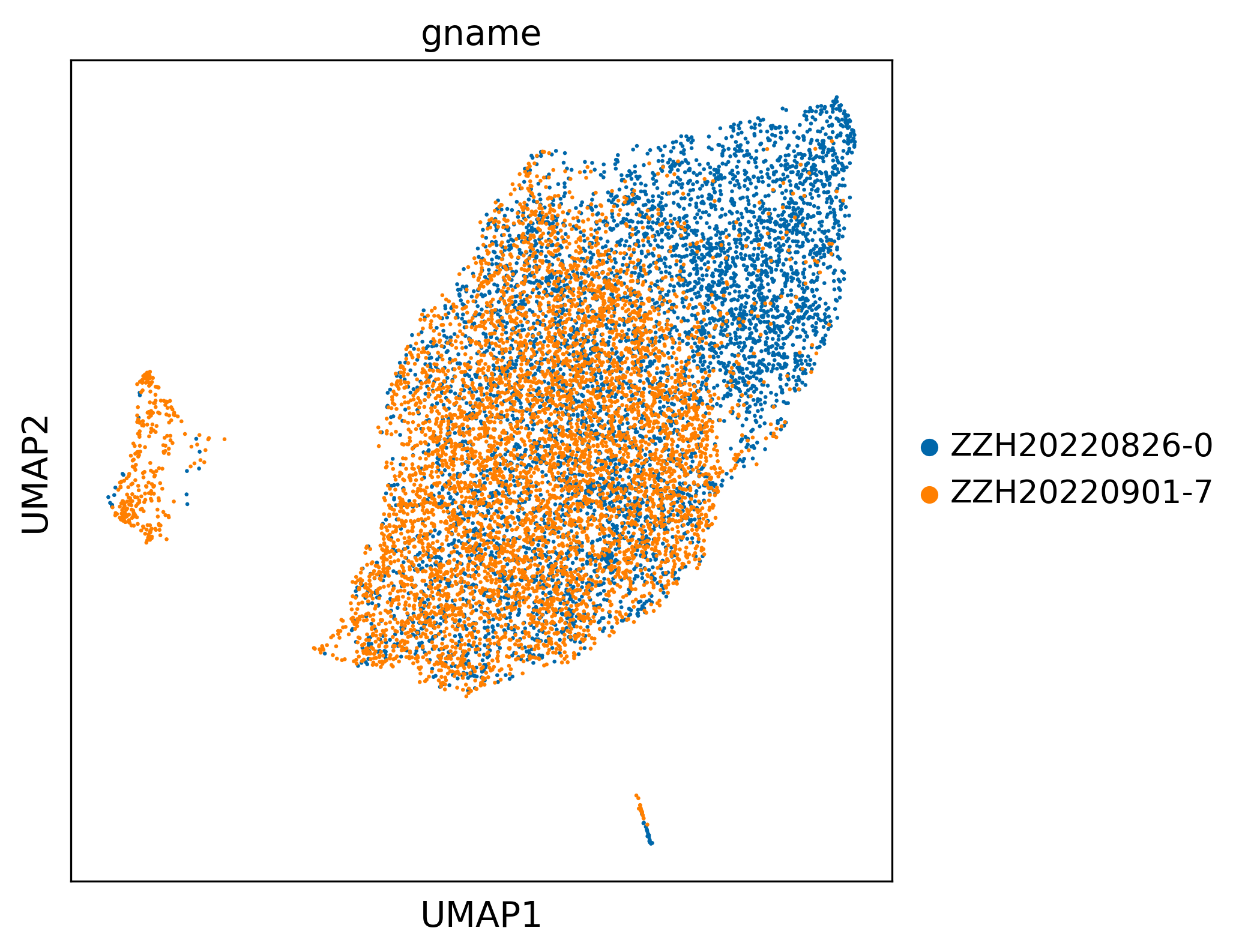

Supplement: Supplementary file 1 [file DataSheet_1.zip › Single-cell sequencing analysis/Neutrophils/P22082602_umap_groups.png]

sample

UMAP2

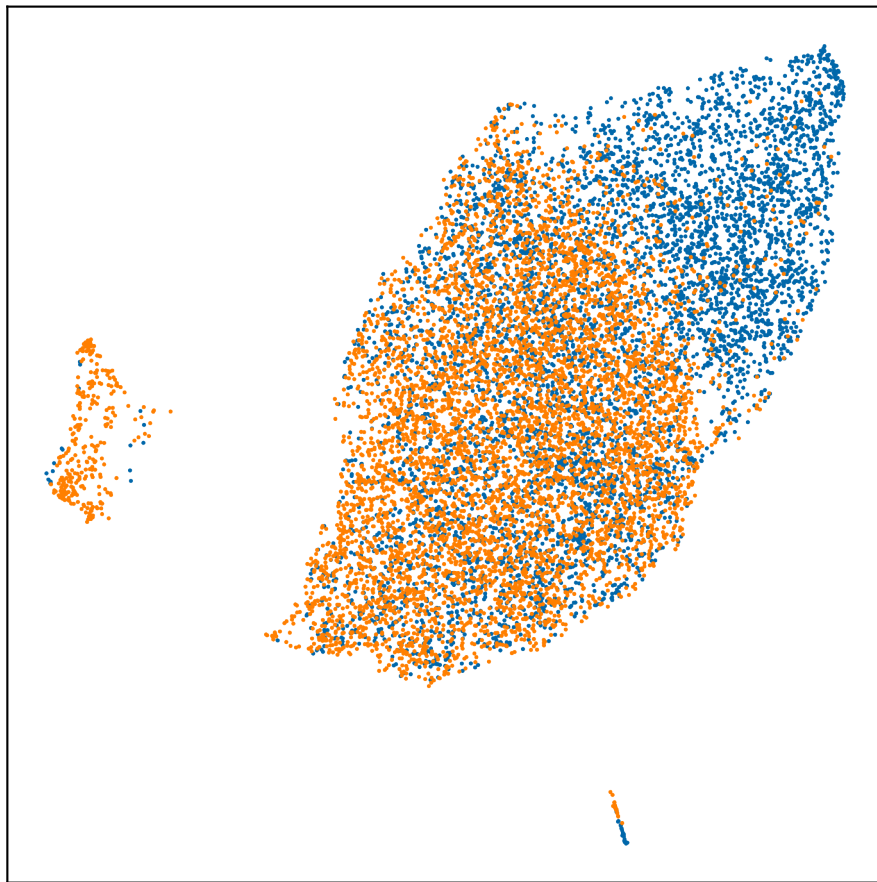

● ZZH20220826-0

● ZZH20220901-7

UMAP1

Supplement: Supplementary file 1 [file DataSheet_1.zip › Single-cell sequencing analysis/Neutrophils/P22082602_umap_samples.pdf]

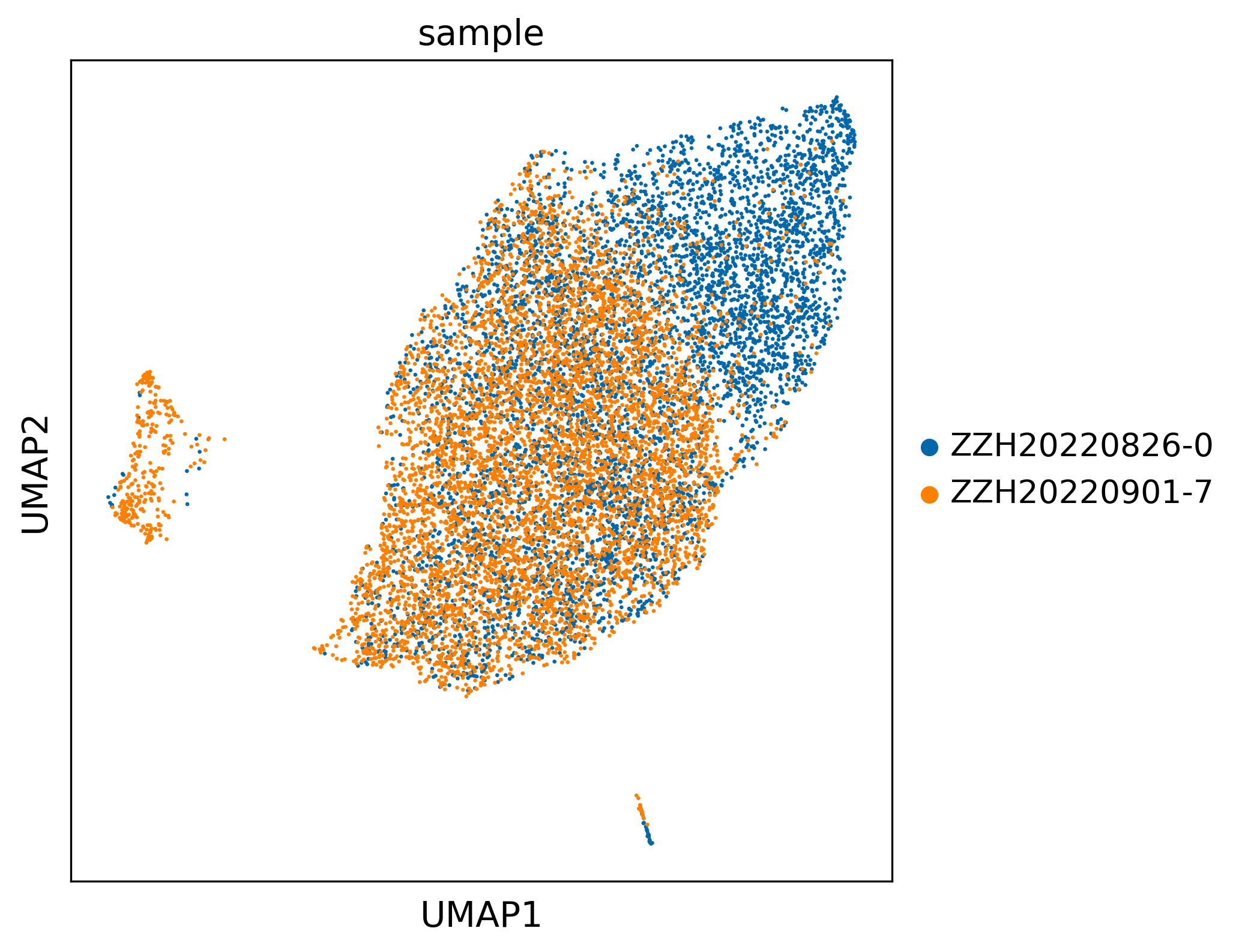

Supplement: Supplementary file 1 [file DataSheet_1.zip › Single-cell sequencing analysis/Neutrophils/P22082602_umap_samples.png]

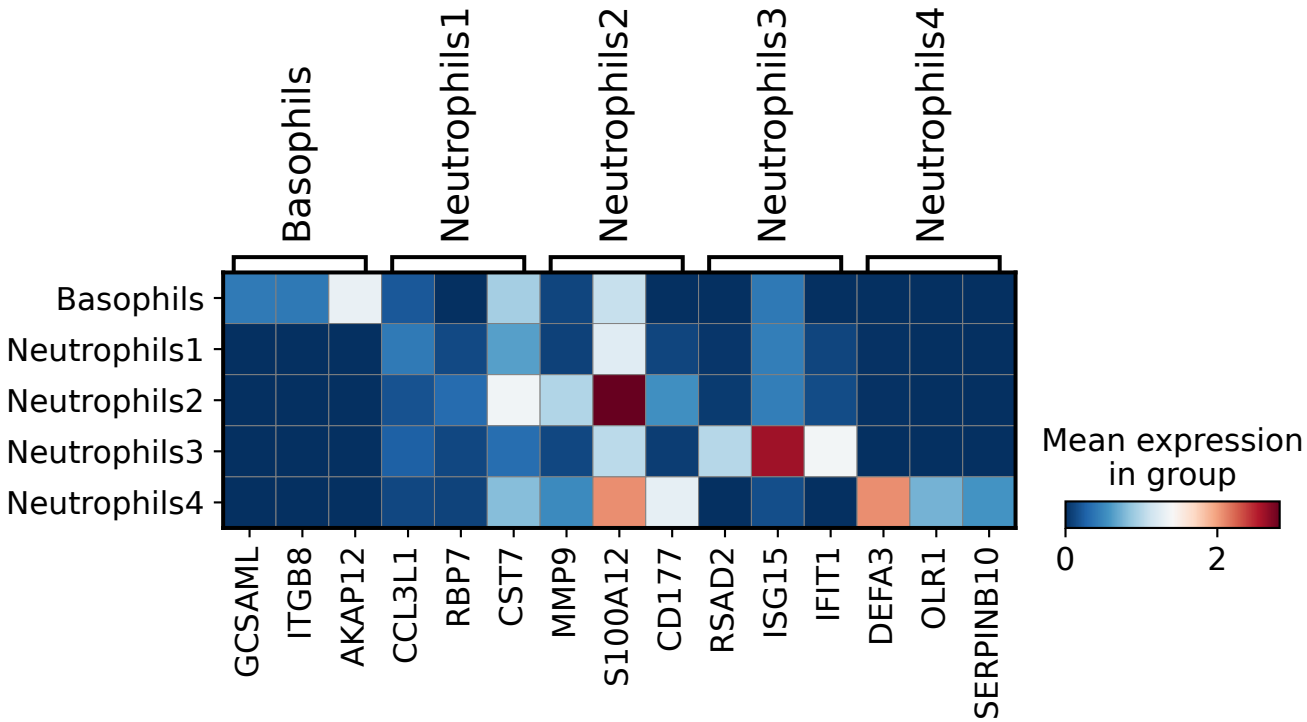

Supplement: Supplementary file 1 [file DataSheet_1.zip › Single-cell sequencing analysis/Neutrophils/P22082602_Zscore_matrixplot.pdf]

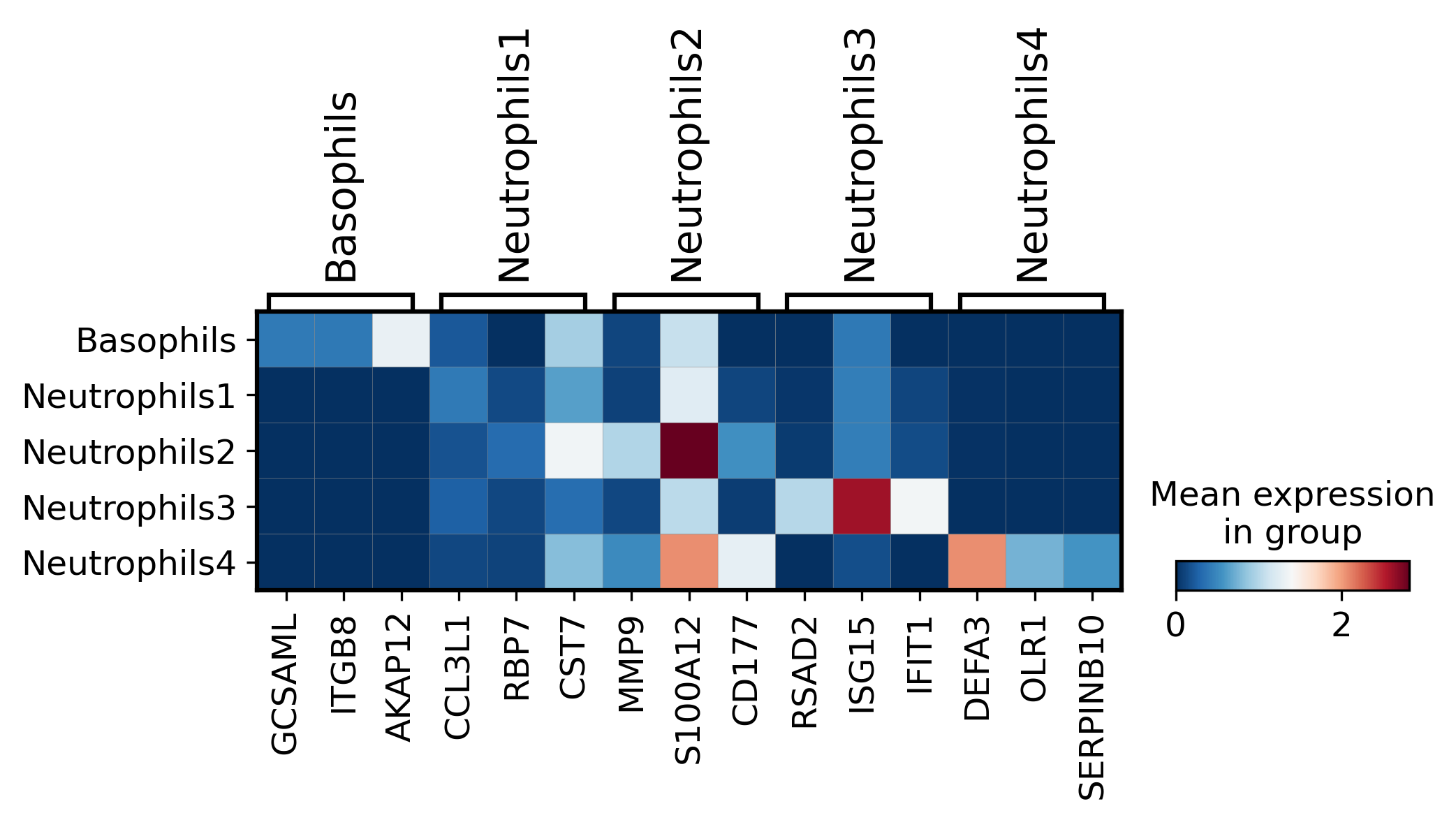

Supplement: Supplementary file 1 [file DataSheet_1.zip › Single-cell sequencing analysis/Neutrophils/P22082602_Zscore_matrixplot.png]

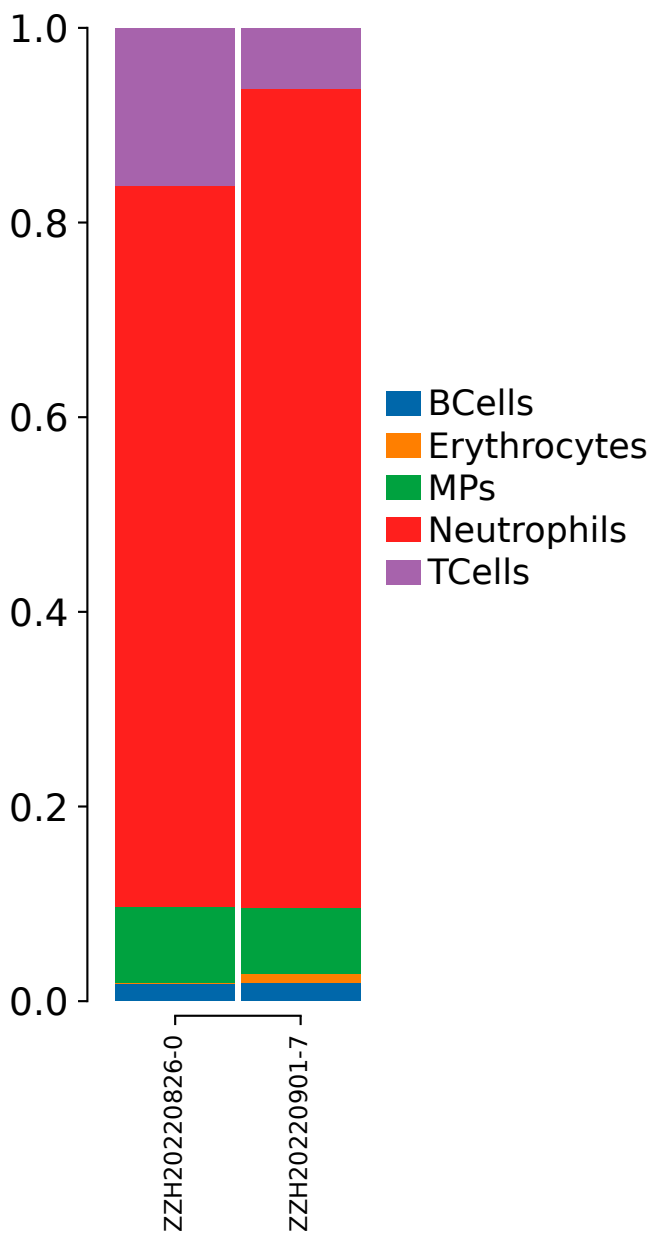

Supplement: Supplementary file 1 [file DataSheet_1.zip › Single-cell sequencing analysis/PBMCs/P22082602_group_PercentPerCell.pdf]

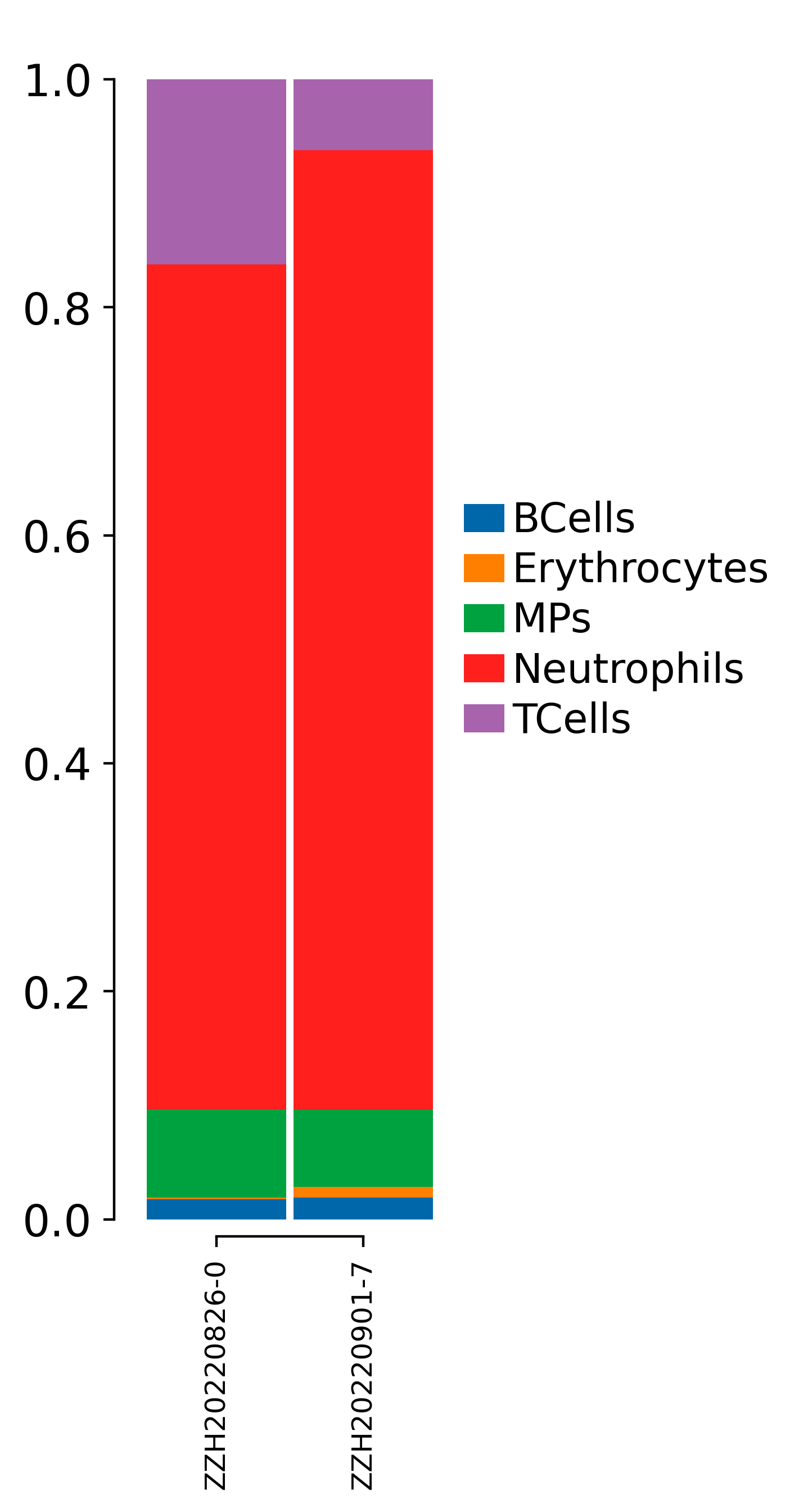

Supplement: Supplementary file 1 [file DataSheet_1.zip › Single-cell sequencing analysis/PBMCs/P22082602_group_PercentPerCell.png]

cluster

UMAP2

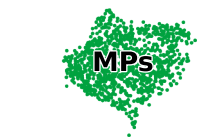

**MPs**

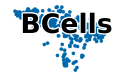

**BCells**

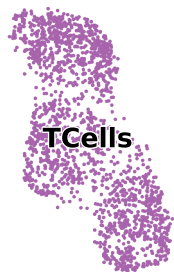

**TCells**

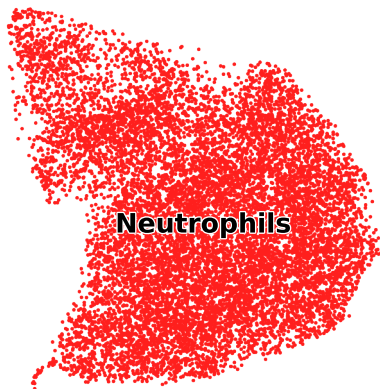

**Neutrophils**

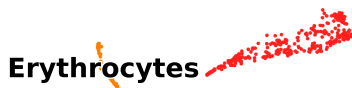

**Erythrocytes**

UMAP1

Supplement: Supplementary file 1 [file DataSheet_1.zip › Single-cell sequencing analysis/PBMCs/P22082602_labumap.pdf]

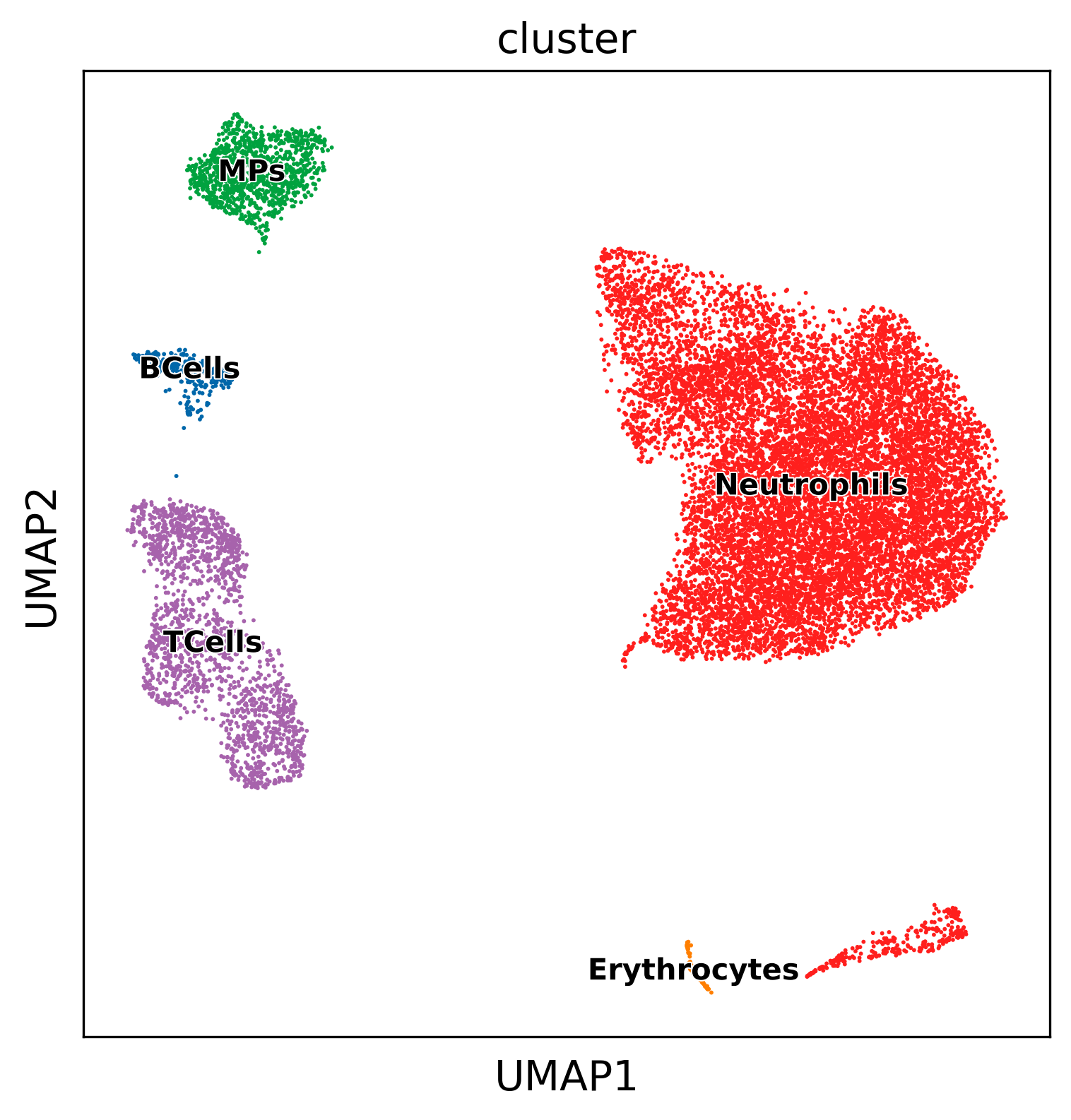

Supplement: Supplementary file 1 [file DataSheet_1.zip › Single-cell sequencing analysis/PBMCs/P22082602_labumap.png]

cluster

UMAP2

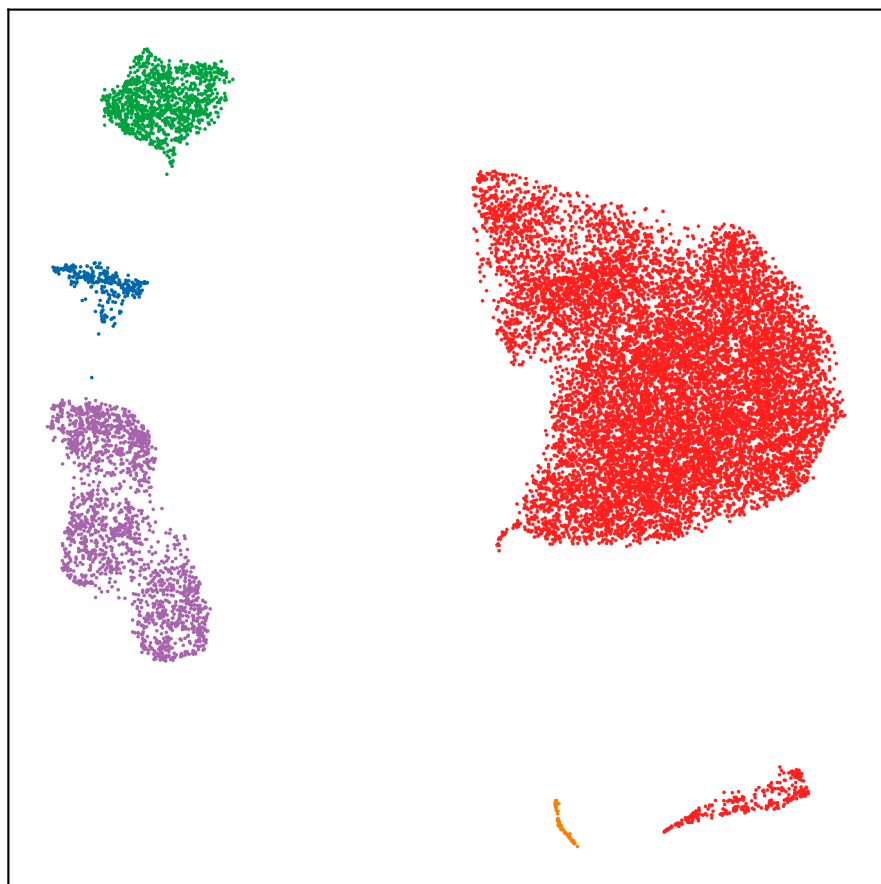

UMAP1

- BCells
- Erythrocytes
- MPs
- Neutrophils
- TCells

Supplement: Supplementary file 1 [file DataSheet_1.zip › Single-cell sequencing analysis/PBMCs/P22082602_rlabumap.pdf]

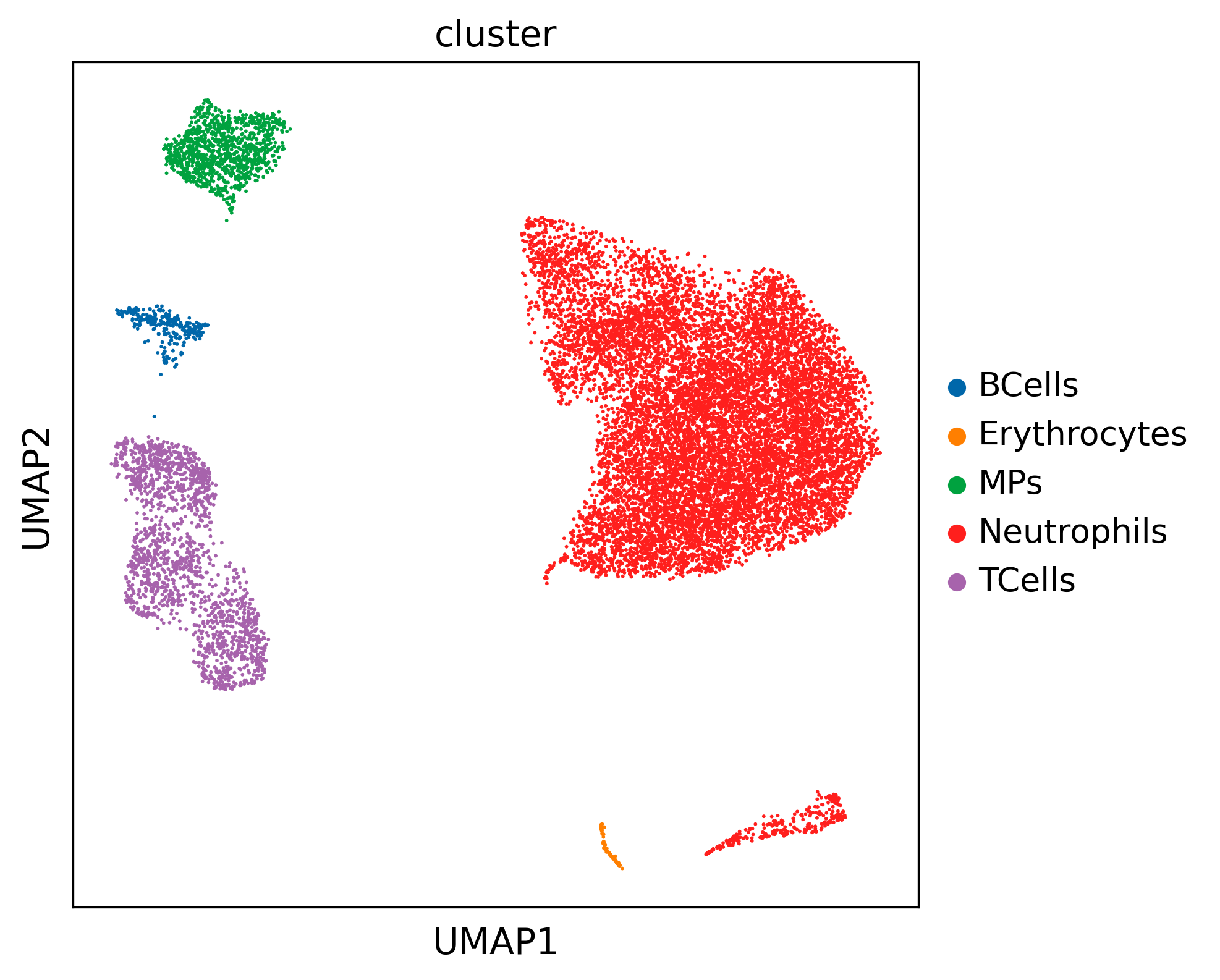

Supplement: Supplementary file 1 [file DataSheet_1.zip › Single-cell sequencing analysis/PBMCs/P22082602_rlabumap.png]

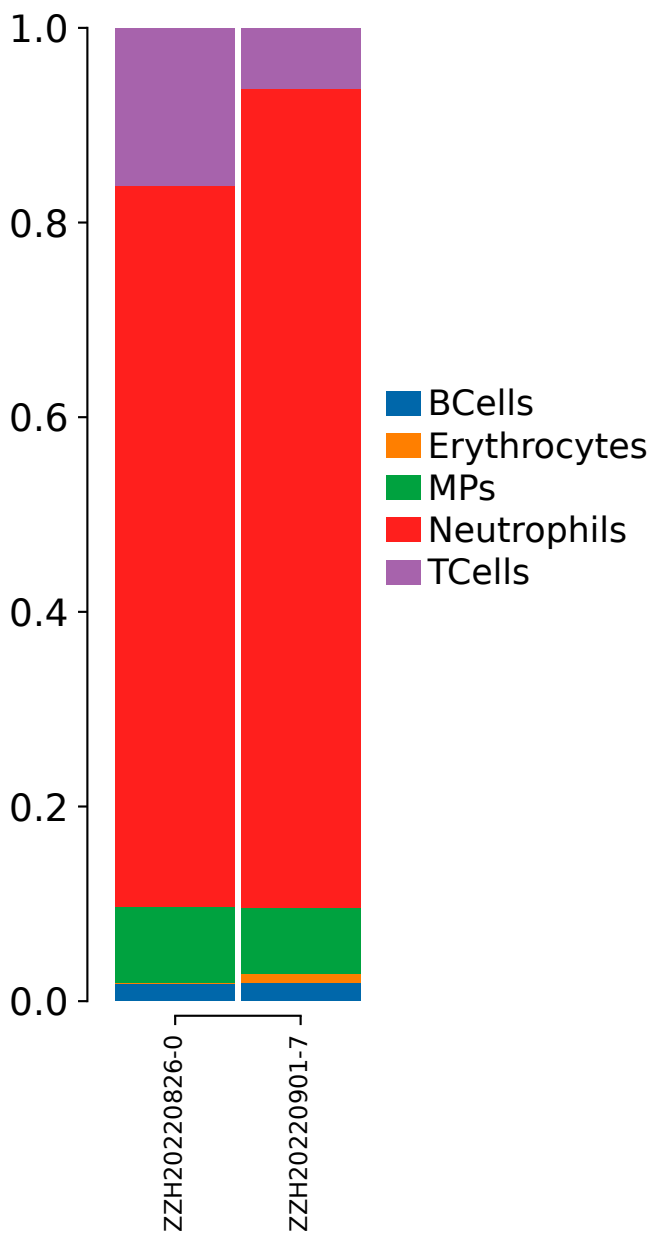

Supplement: Supplementary file 1 [file DataSheet_1.zip › Single-cell sequencing analysis/PBMCs/P22082602_sample_PercentPerCell.pdf]

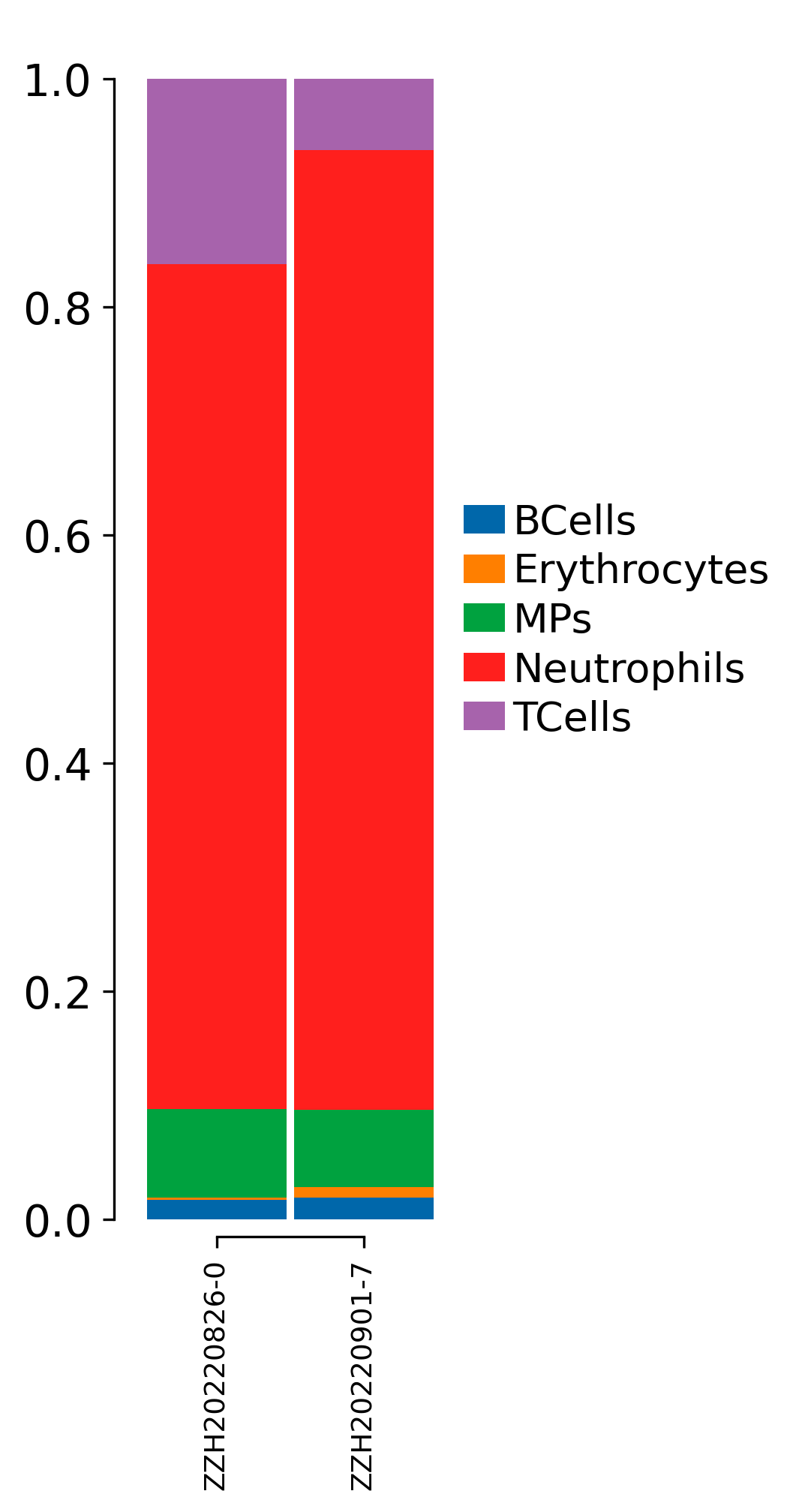

Supplement: Supplementary file 1 [file DataSheet_1.zip › Single-cell sequencing analysis/PBMCs/P22082602_sample_PercentPerCell.png]

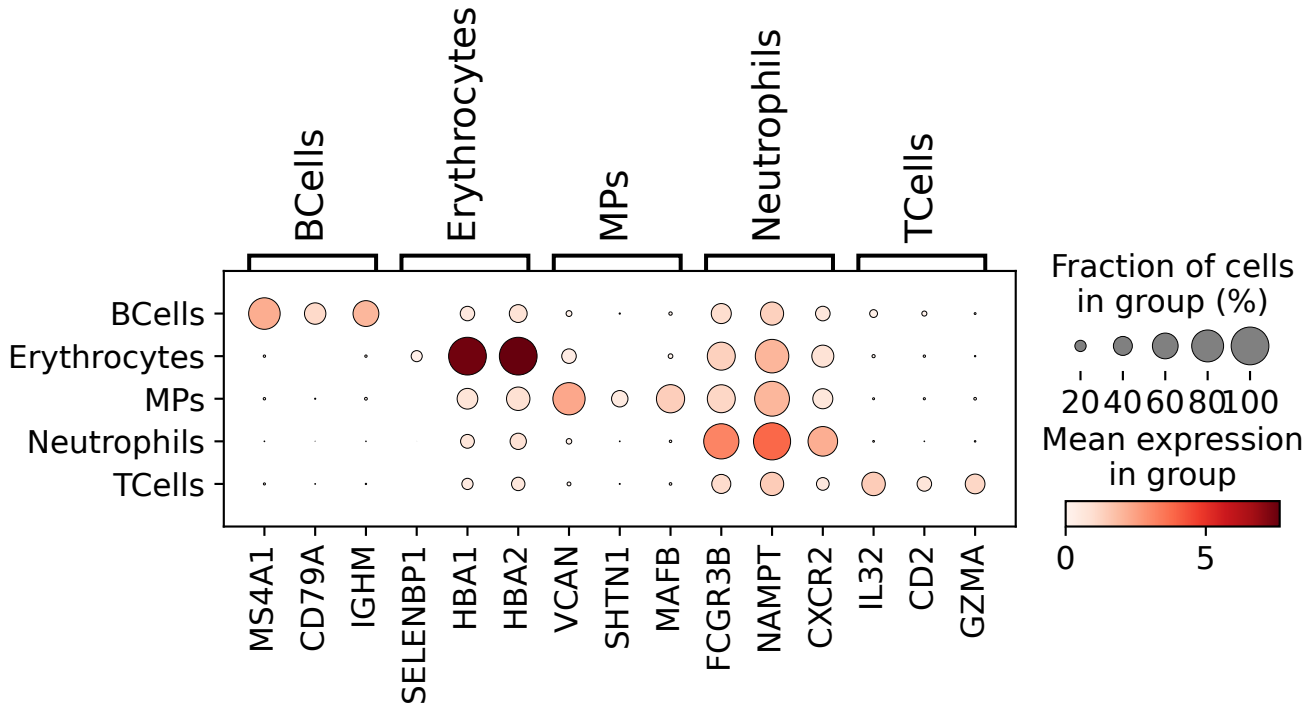

Supplement: Supplementary file 1 [file DataSheet_1.zip › Single-cell sequencing analysis/PBMCs/P22082602_TopMarkergenedotplot.pdf]

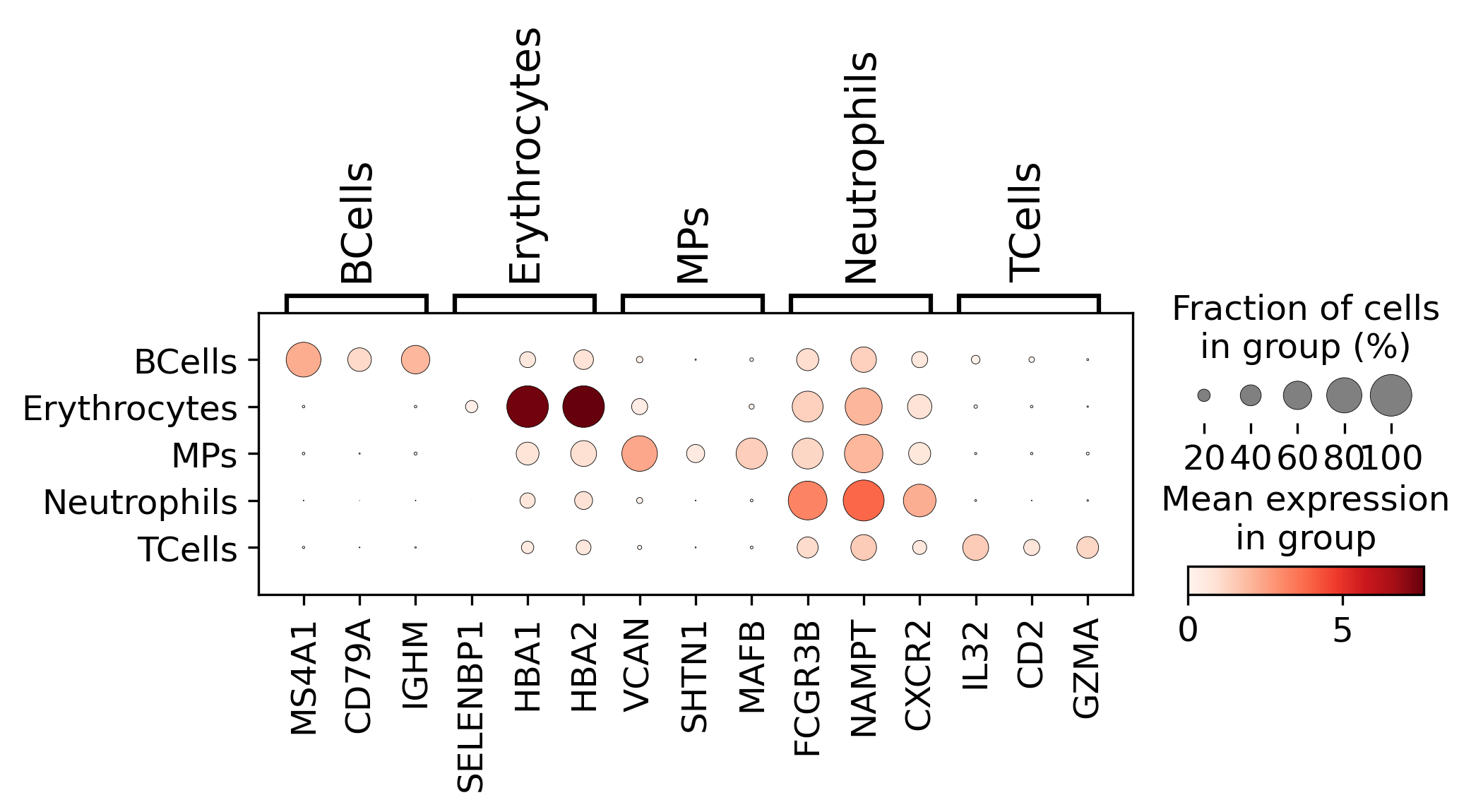

Supplement: Supplementary file 1 [file DataSheet_1.zip › Single-cell sequencing analysis/PBMCs/P22082602_TopMarkergenedotplot.png]

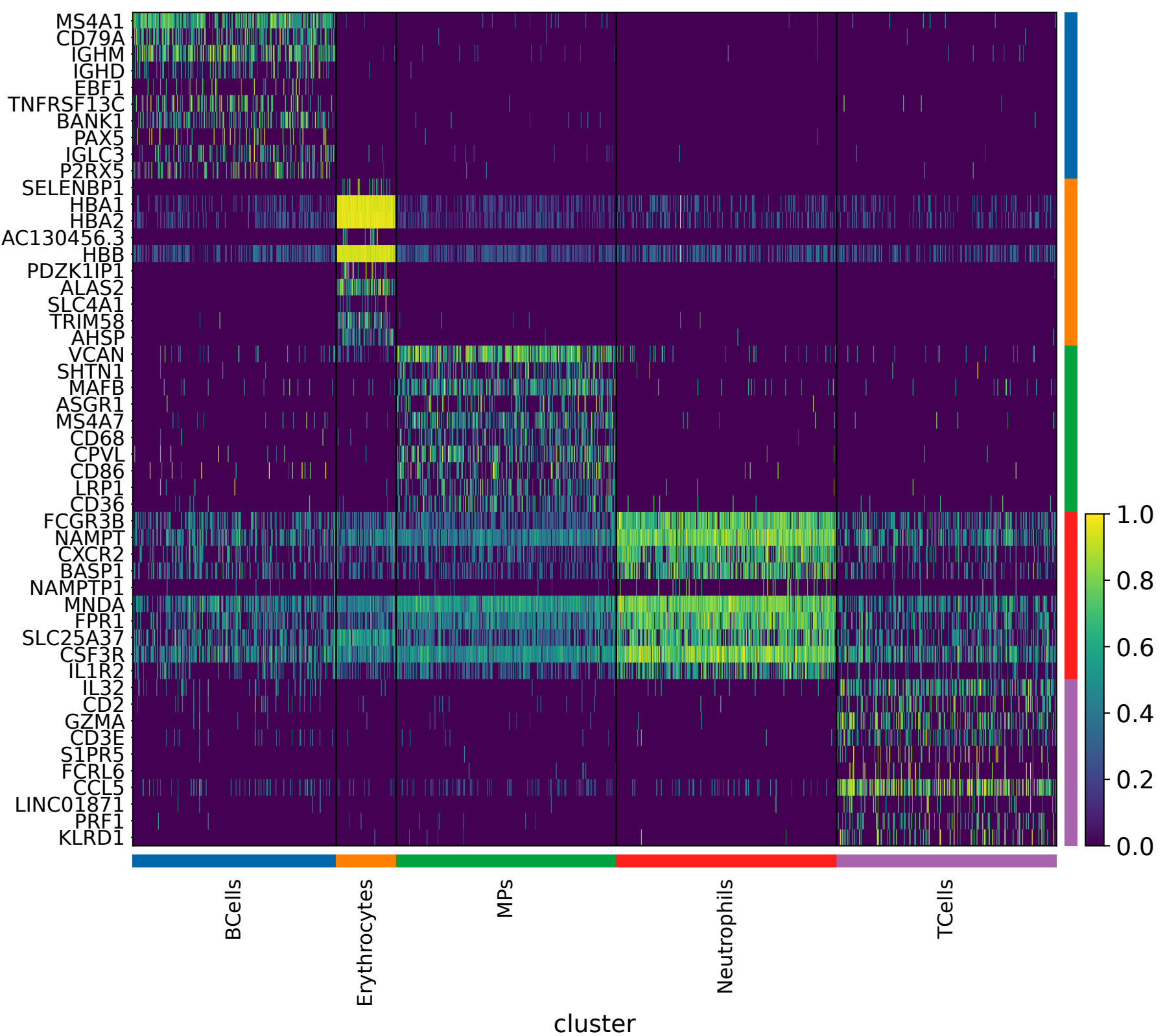

Supplement: Supplementary file 1 [file DataSheet_1.zip › Single-cell sequencing analysis/PBMCs/P22082602_TopMarkergeneHeatmap.pdf]

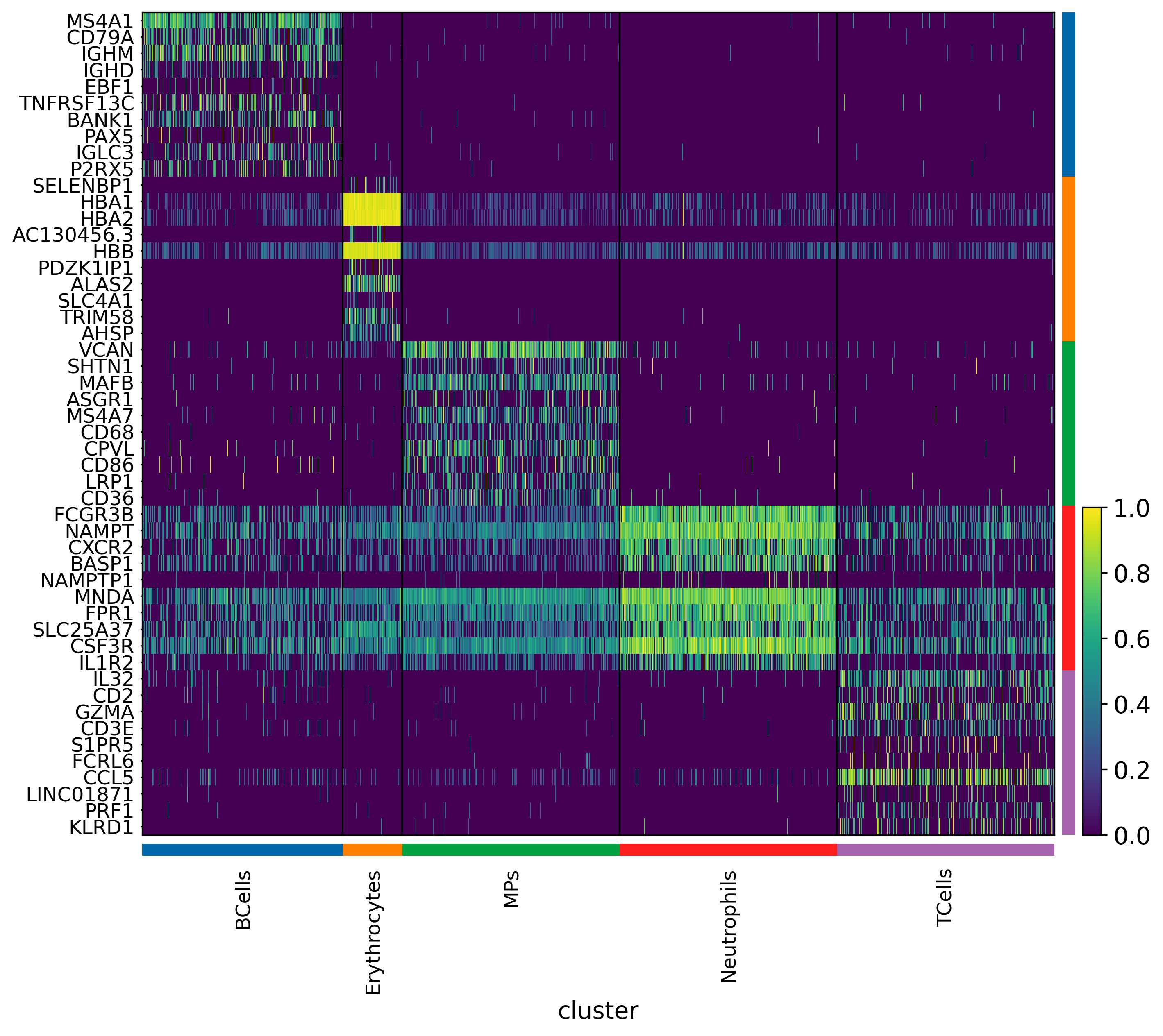

Supplement: Supplementary file 1 [file DataSheet_1.zip › Single-cell sequencing analysis/PBMCs/P22082602_TopMarkergeneHeatmap.png]

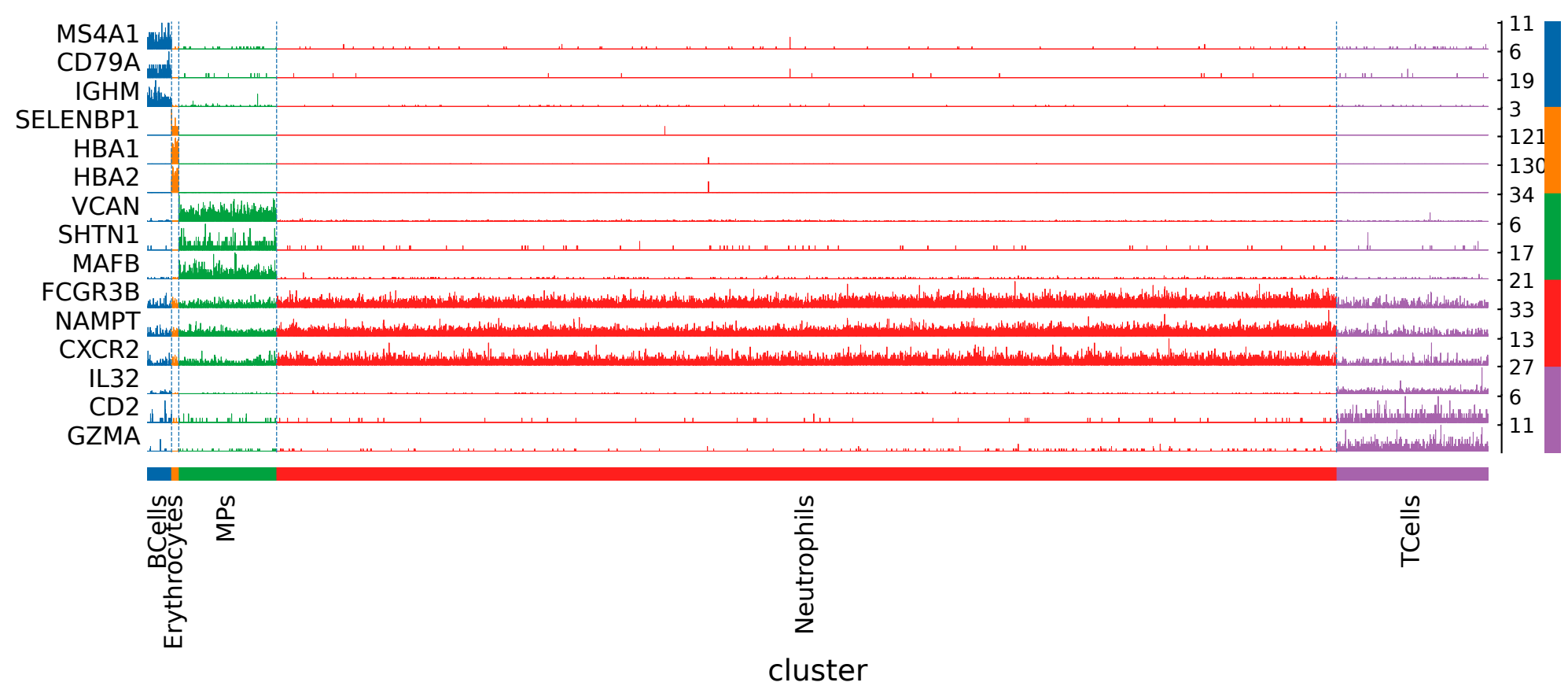

Supplement: Supplementary file 1 [file DataSheet_1.zip › Single-cell sequencing analysis/PBMCs/P22082602_TopMarkergeneTracksplot.pdf]

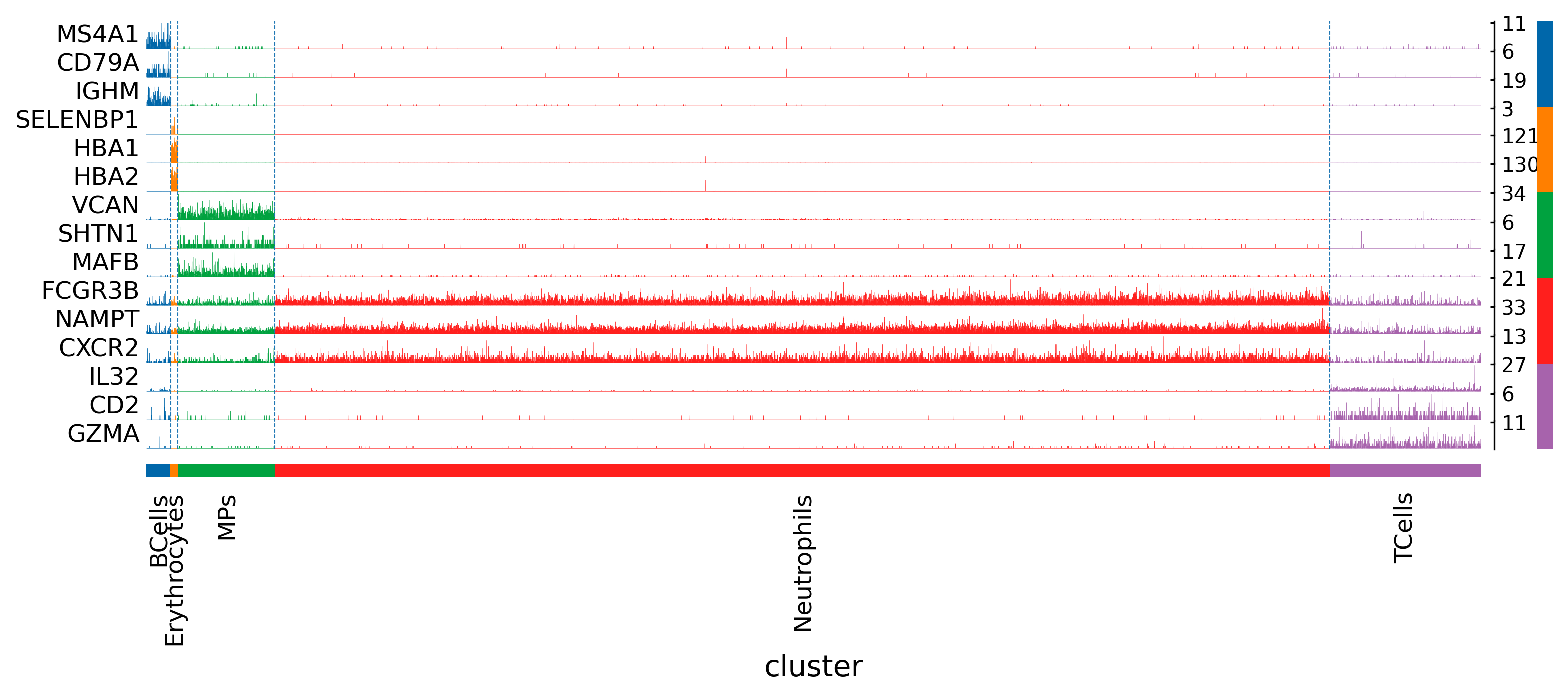

Supplement: Supplementary file 1 [file DataSheet_1.zip › Single-cell sequencing analysis/PBMCs/P22082602_TopMarkergeneTracksplot.png]

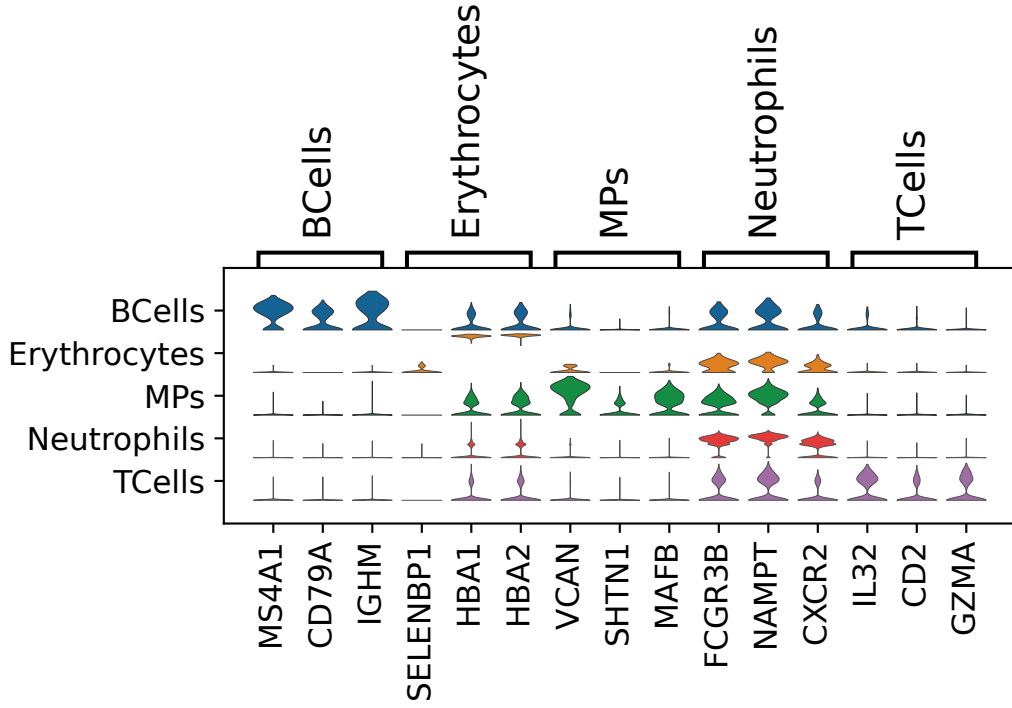

Supplement: Supplementary file 1 [file DataSheet_1.zip › Single-cell sequencing analysis/PBMCs/P22082602_TopStackedViolin.pdf]

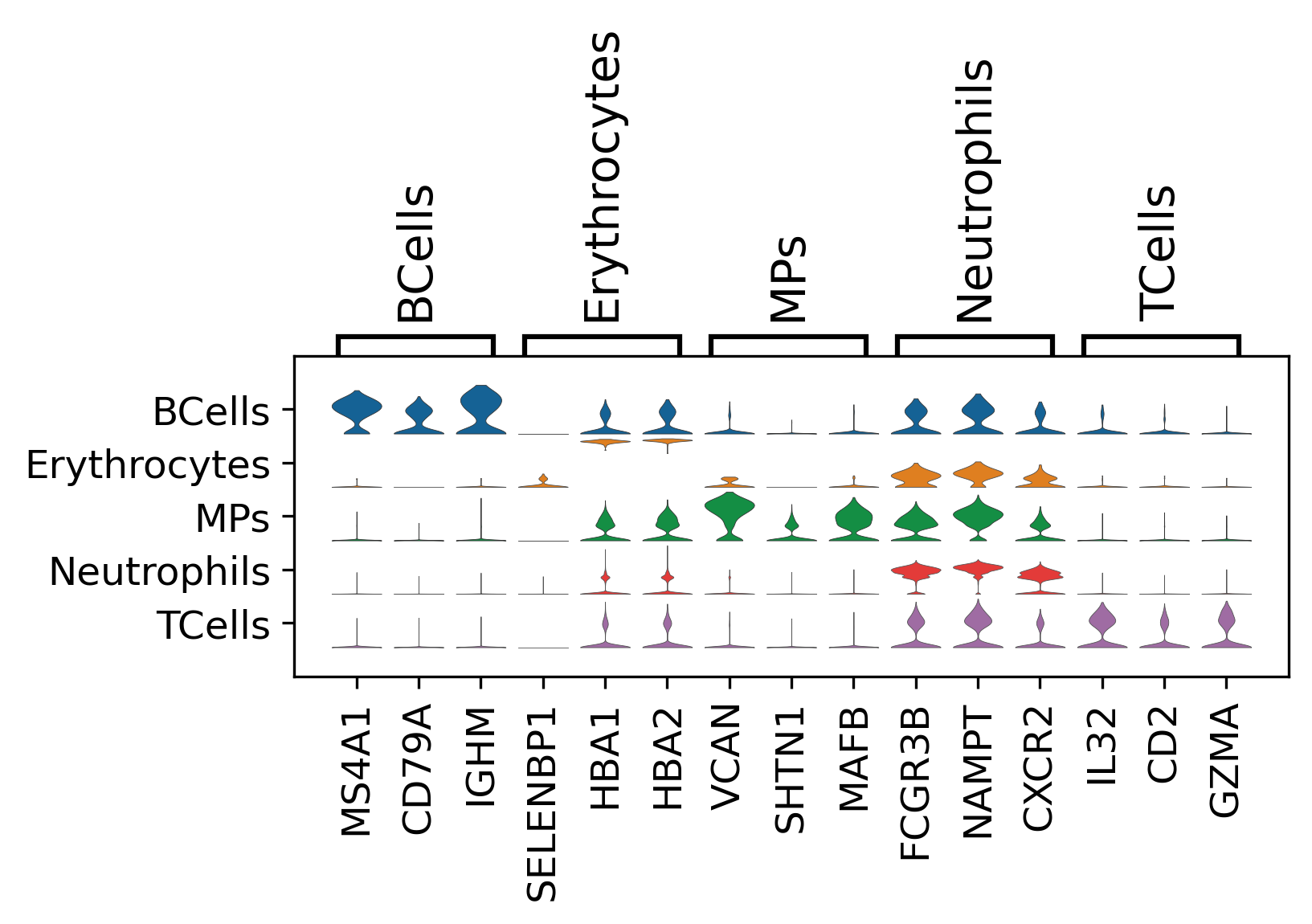

Supplement: Supplementary file 1 [file DataSheet_1.zip › Single-cell sequencing analysis/PBMCs/P22082602_TopStackedViolin.png]

gname

UMAP2

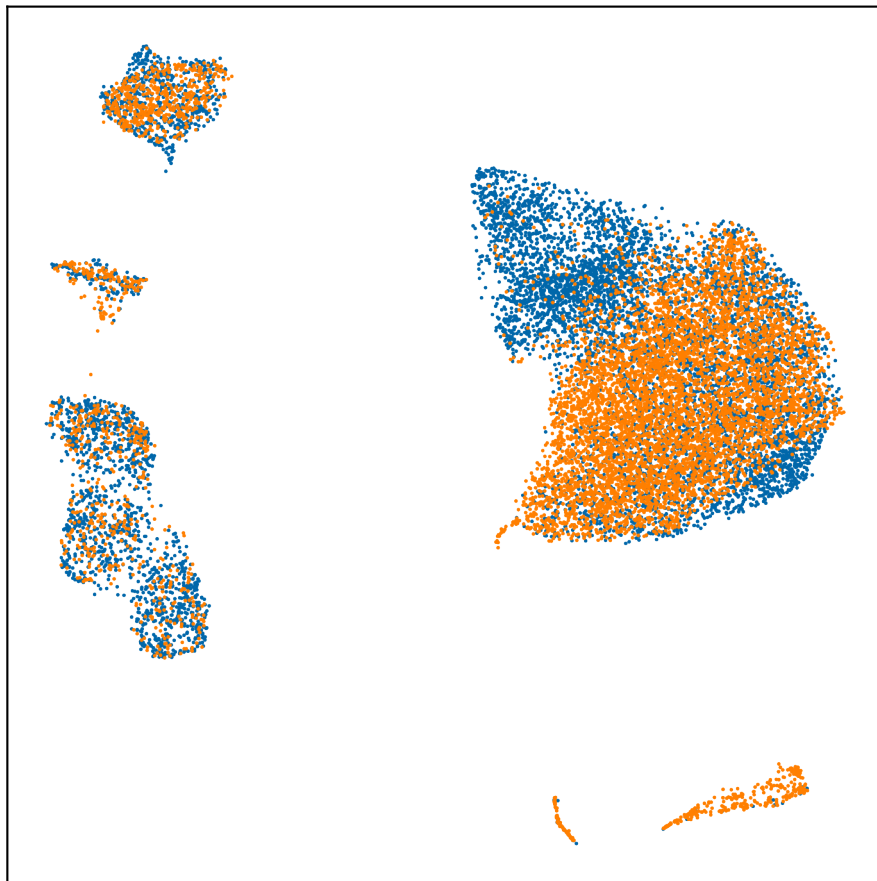

UMAP1

- ZZH20220826-0
- ZZH20220901-7

Supplement: Supplementary file 1 [file DataSheet_1.zip › Single-cell sequencing analysis/PBMCs/P22082602_umap_groups.pdf]

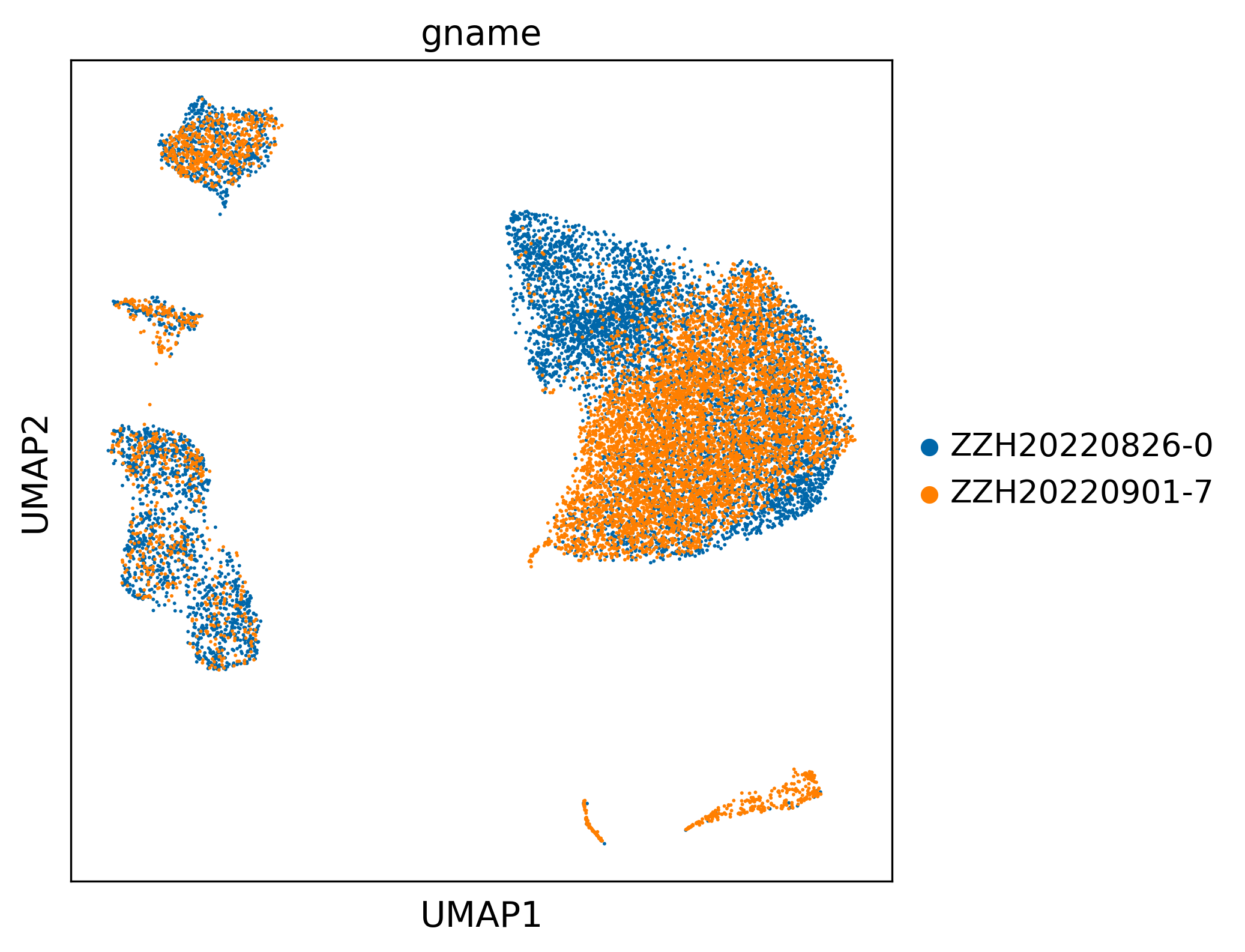

Supplement: Supplementary file 1 [file DataSheet_1.zip › Single-cell sequencing analysis/PBMCs/P22082602_umap_groups.png]

sample

UMAP2

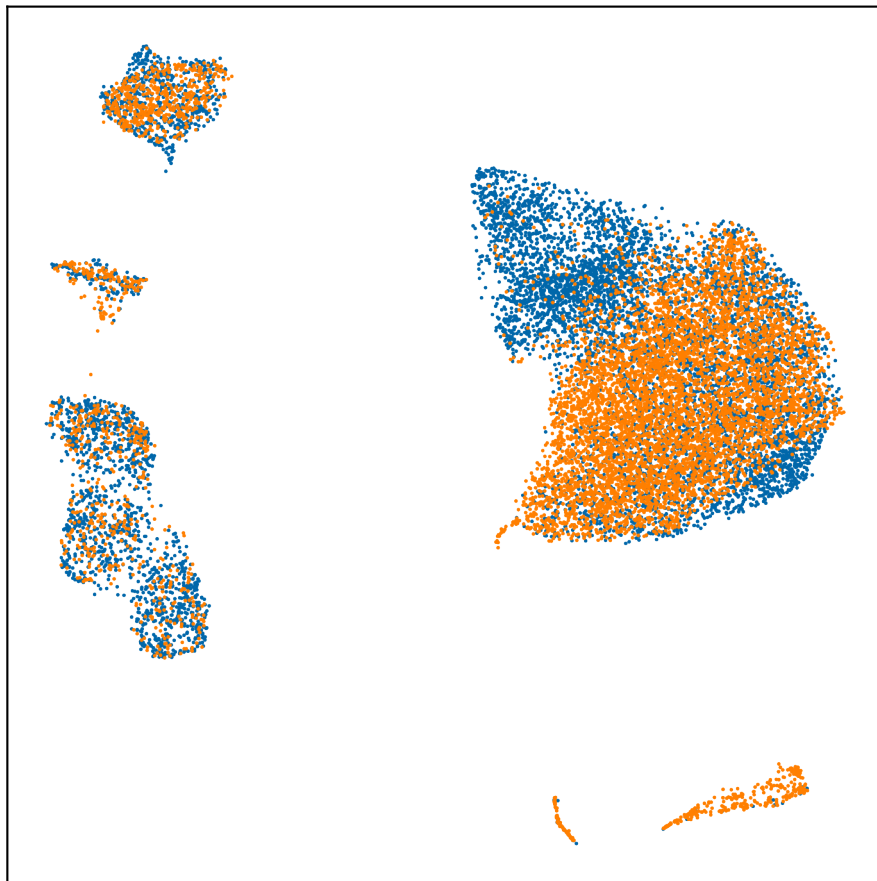

UMAP1

- ZZH20220826-0
- ZZH20220901-7

Supplement: Supplementary file 1 [file DataSheet_1.zip › Single-cell sequencing analysis/PBMCs/P22082602_umap_samples.pdf]

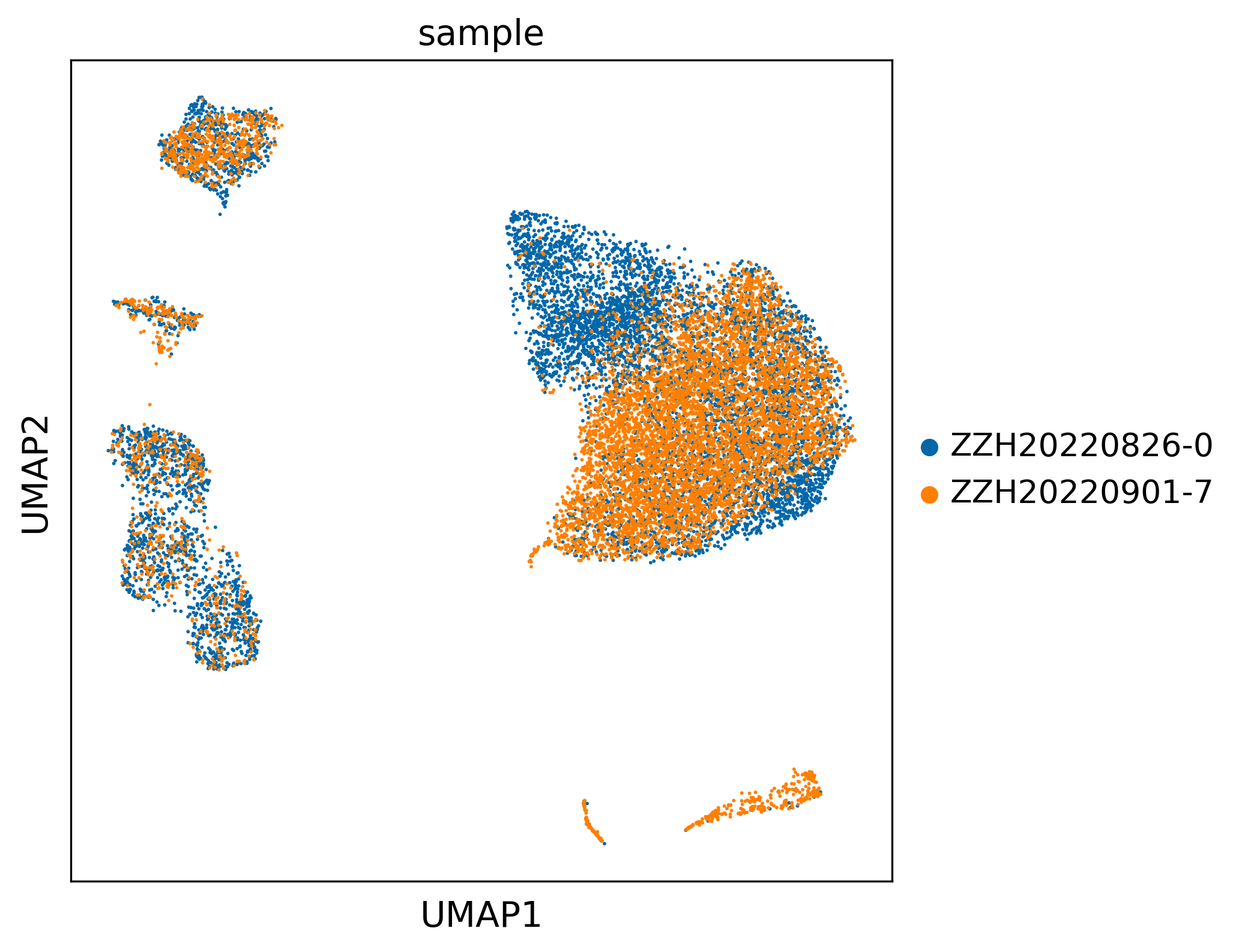

Supplement: Supplementary file 1 [file DataSheet_1.zip › Single-cell sequencing analysis/PBMCs/P22082602_umap_samples.png]

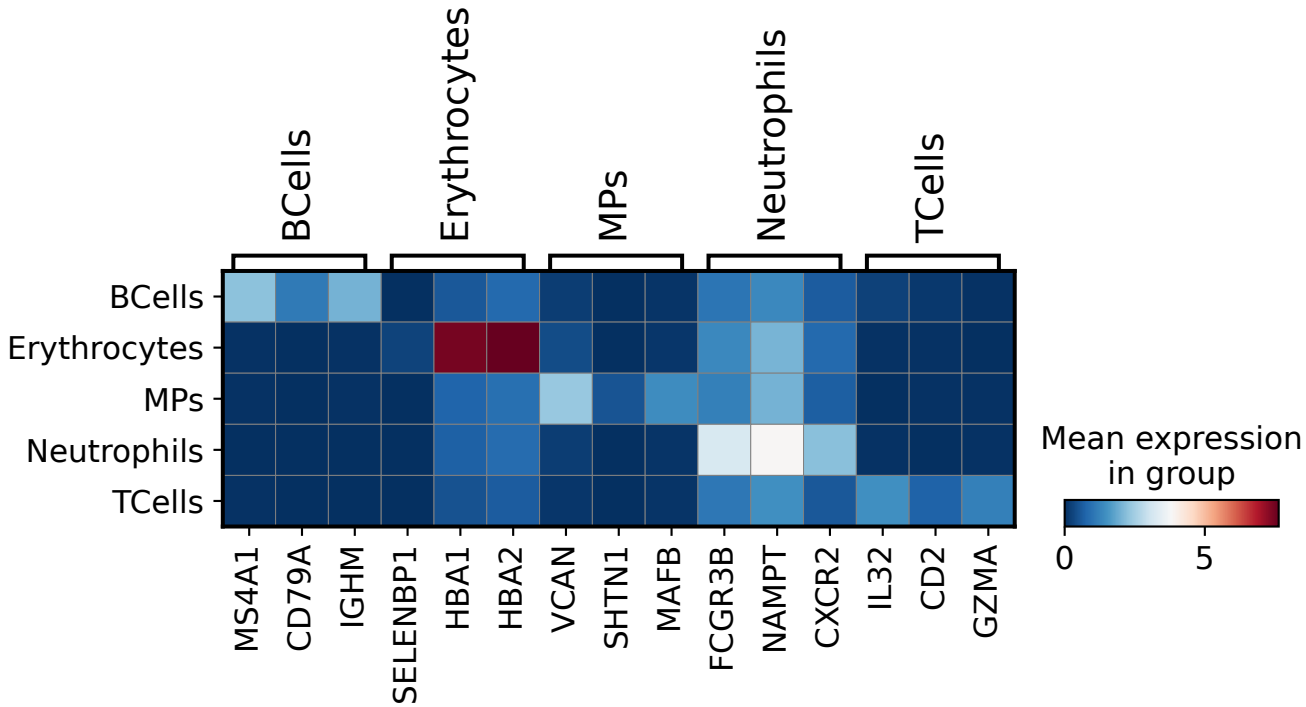

Supplement: Supplementary file 1 [file DataSheet_1.zip › Single-cell sequencing analysis/PBMCs/P22082602_Zscore_matrixplot.pdf]

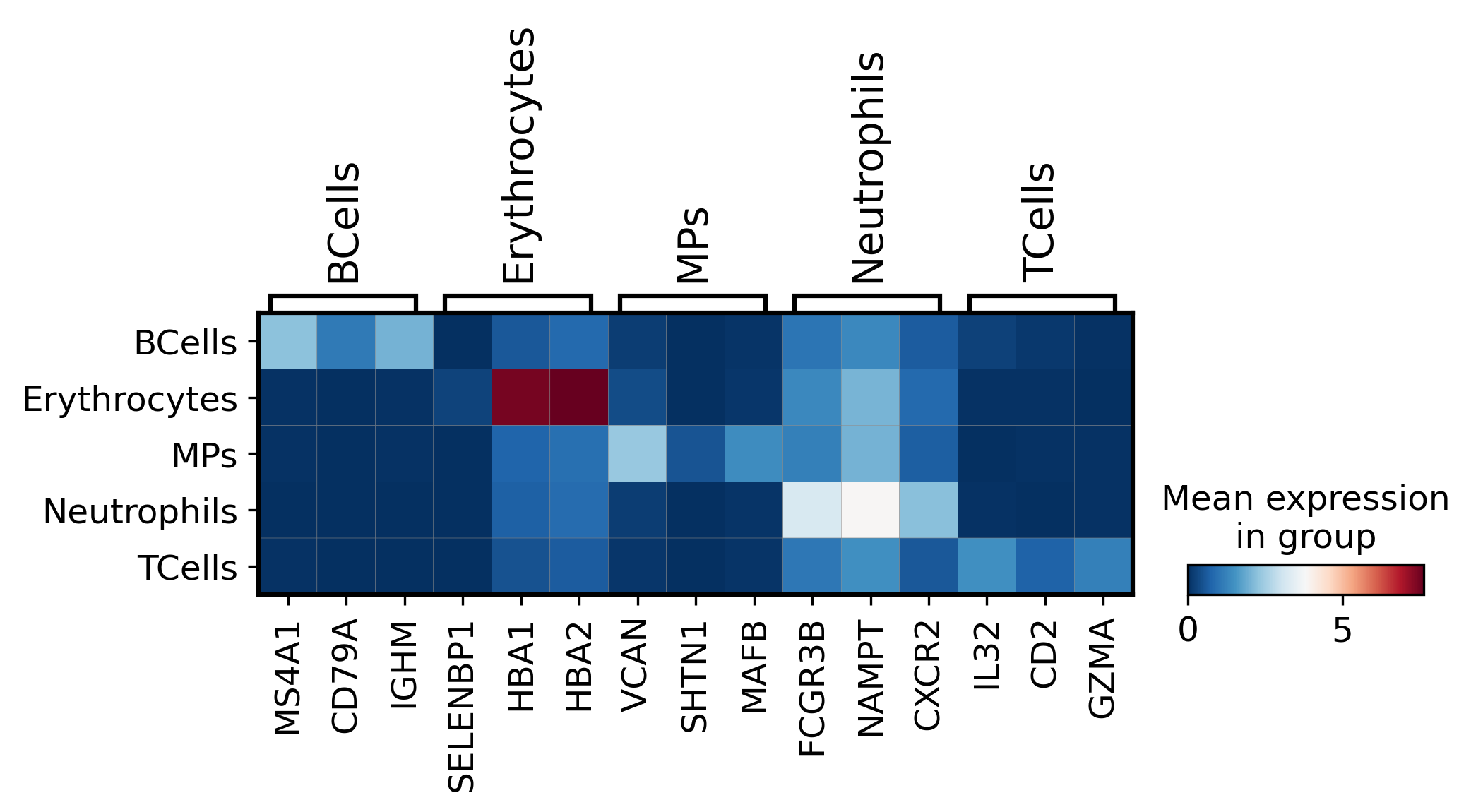

Supplement: Supplementary file 1 [file DataSheet_1.zip › Single-cell sequencing analysis/PBMCs/P22082602_Zscore_matrixplot.png]

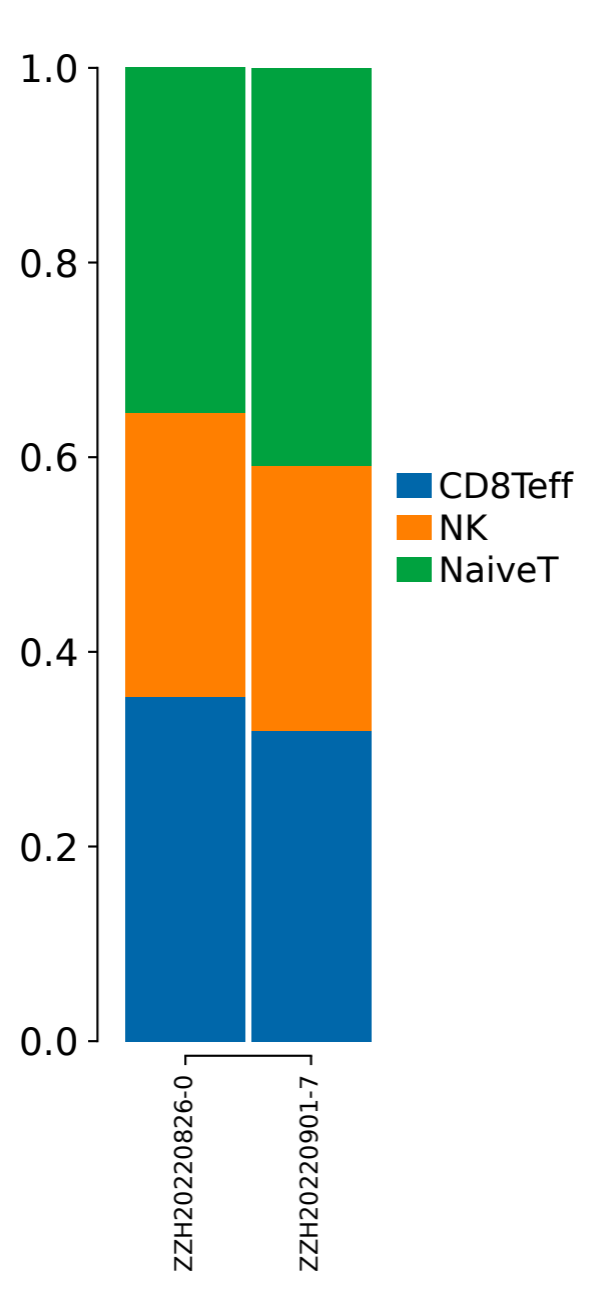

Supplement: Supplementary file 1 [file DataSheet_1.zip › Single-cell sequencing analysis/T cells/P22082602_group_PercentPerCell.pdf]

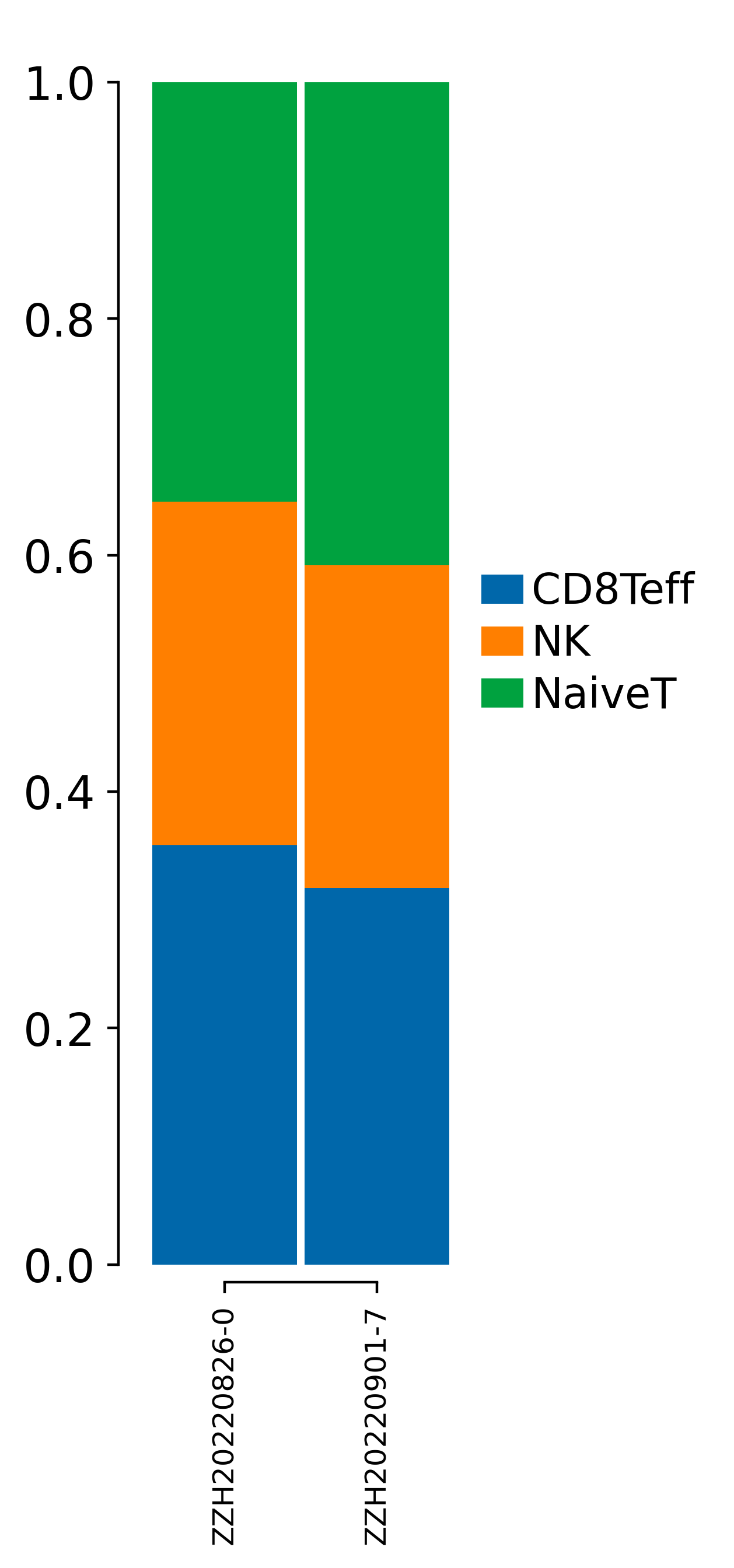

Supplement: Supplementary file 1 [file DataSheet_1.zip › Single-cell sequencing analysis/T cells/P22082602_group_PercentPerCell.png]

cluster

UMAP2

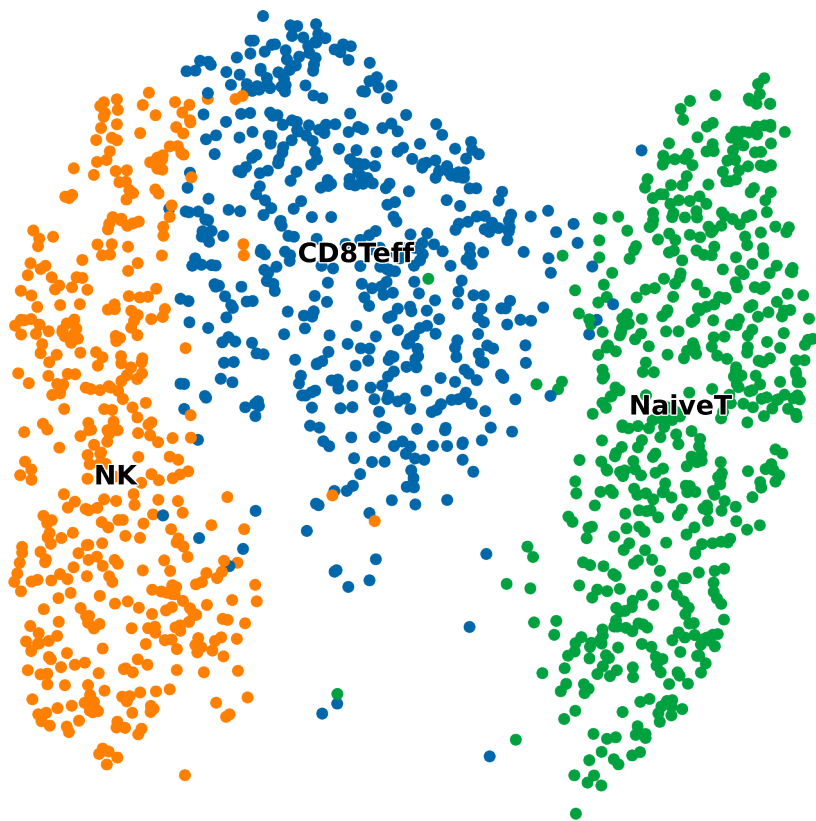

UMAP1

Supplement: Supplementary file 1 [file DataSheet_1.zip › Single-cell sequencing analysis/T cells/P22082602_labumap.pdf]

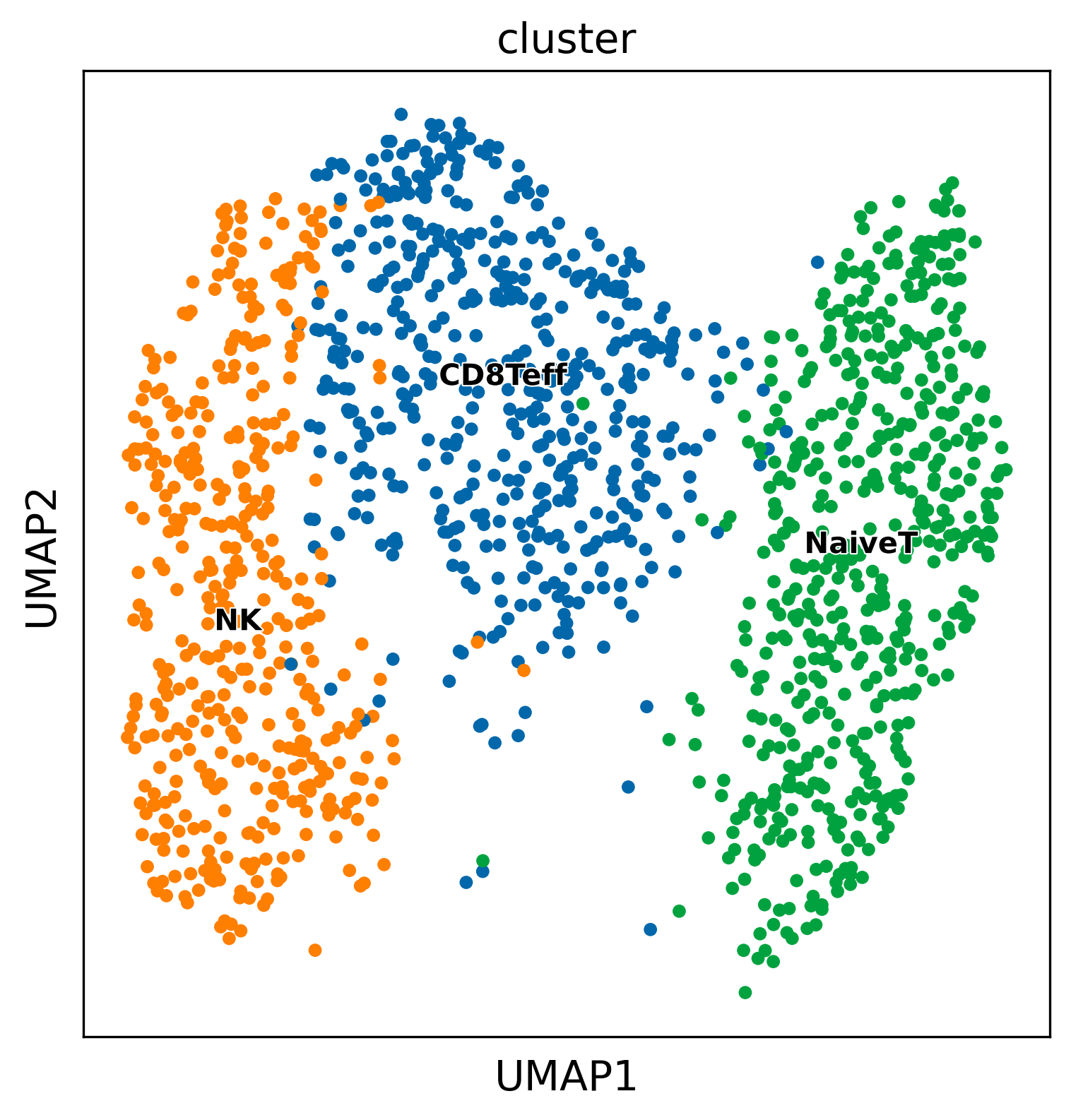

Supplement: Supplementary file 1 [file DataSheet_1.zip › Single-cell sequencing analysis/T cells/P22082602_labumap.png]

cluster

UMAP2

CD8Teff  
NK  
NaiveT

UMAP1

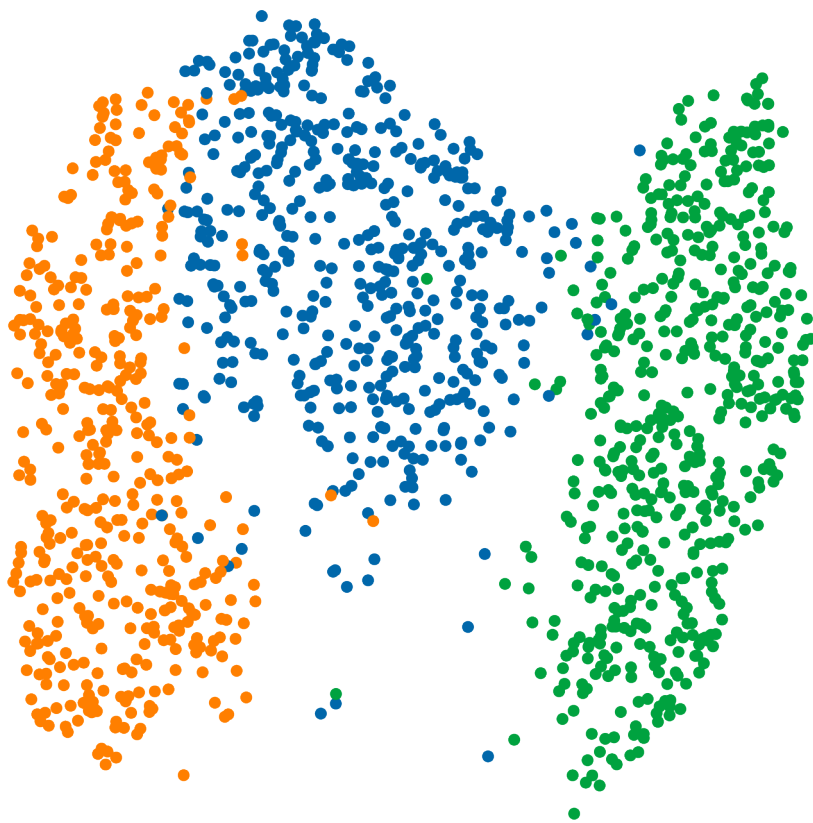

Supplement: Supplementary file 1 [file DataSheet_1.zip › Single-cell sequencing analysis/T cells/P22082602_rlabumap.pdf]

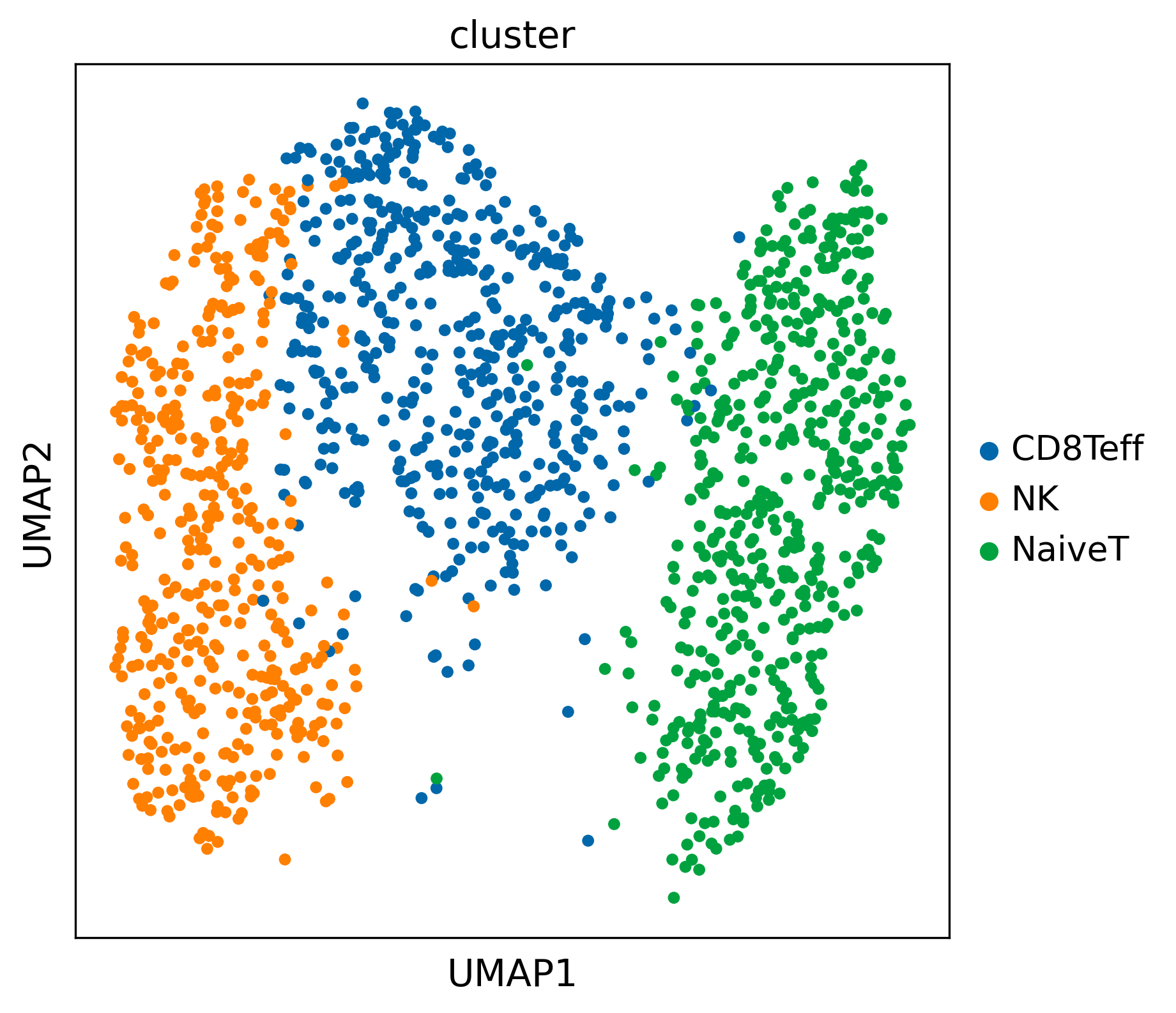

Supplement: Supplementary file 1 [file DataSheet_1.zip › Single-cell sequencing analysis/T cells/P22082602_rlabumap.png]

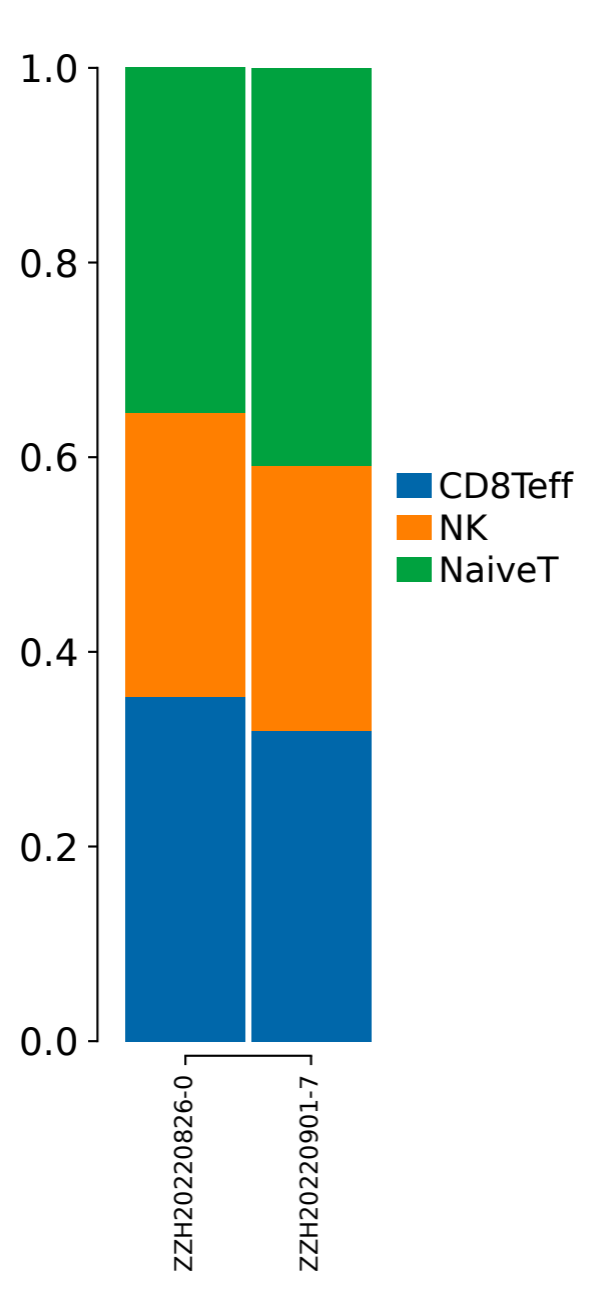

Supplement: Supplementary file 1 [file DataSheet_1.zip › Single-cell sequencing analysis/T cells/P22082602_sample_PercentPerCell.pdf]

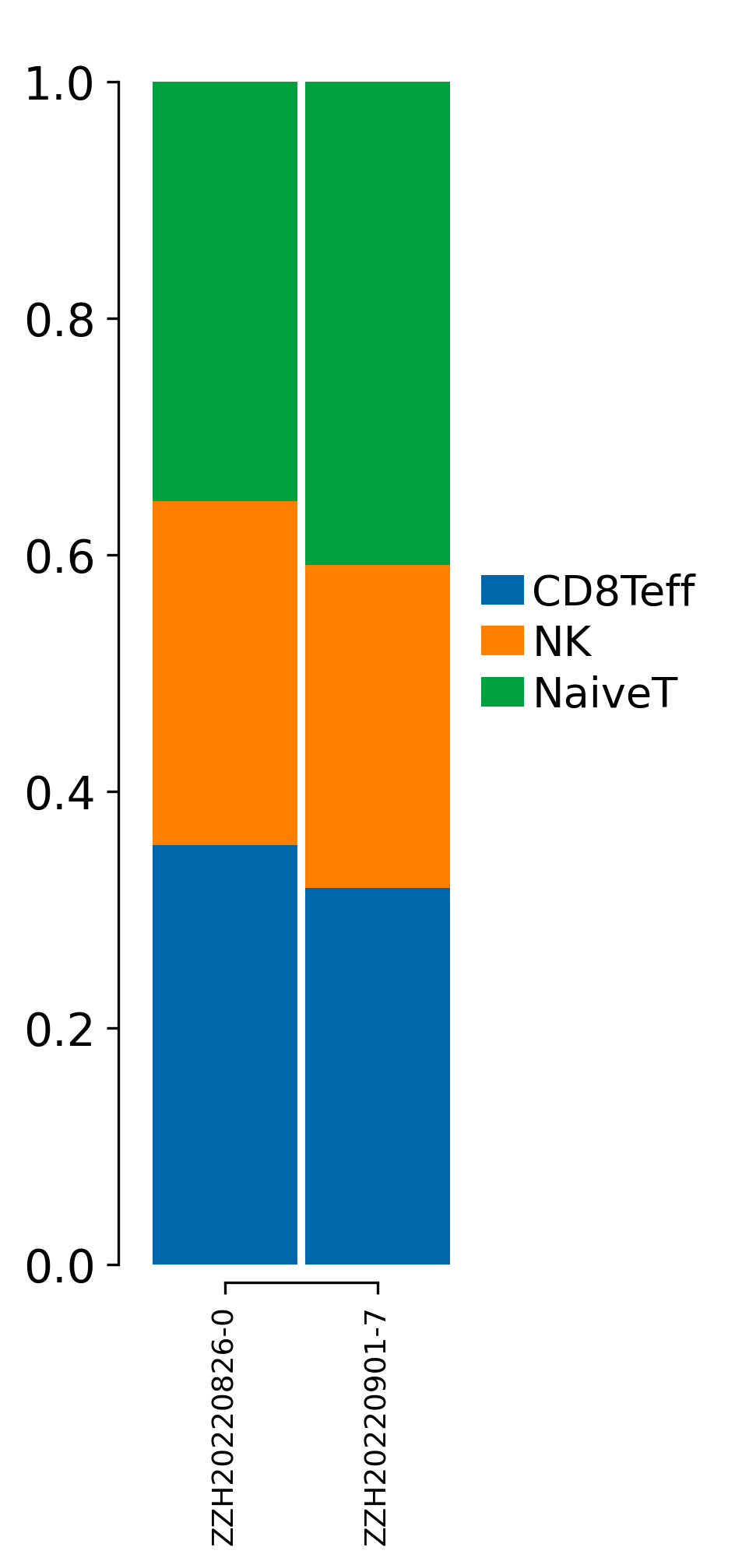

Supplement: Supplementary file 1 [file DataSheet_1.zip › Single-cell sequencing analysis/T cells/P22082602_sample_PercentPerCell.png]

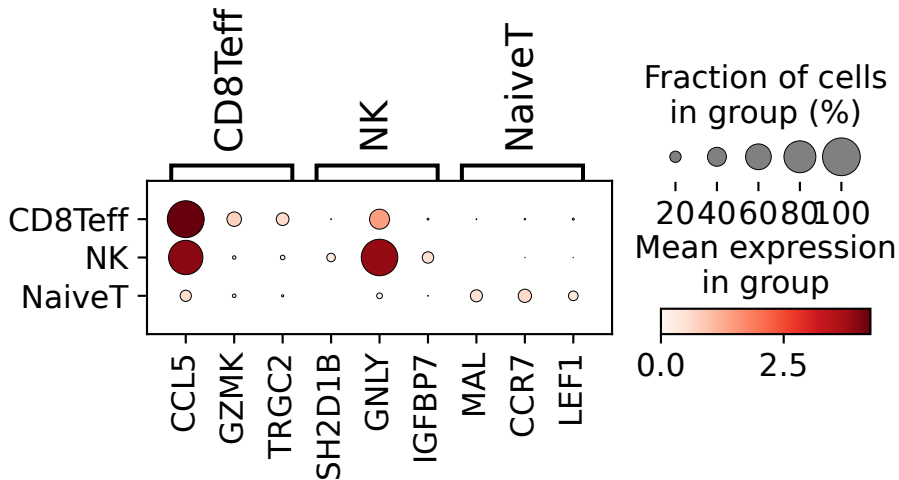

Supplement: Supplementary file 1 [file DataSheet_1.zip › Single-cell sequencing analysis/T cells/P22082602_TopMarkergenedotplot.pdf]

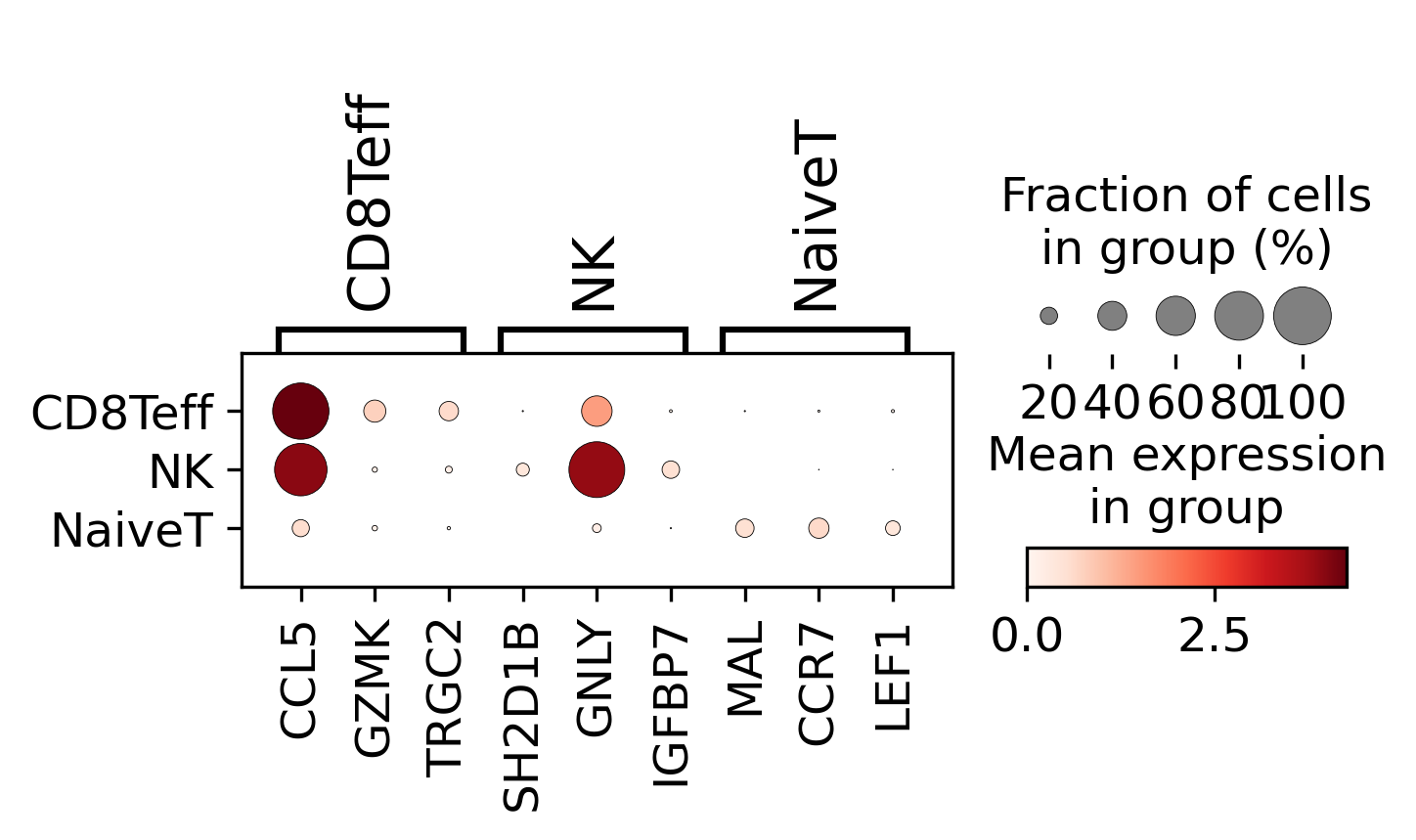

Supplement: Supplementary file 1 [file DataSheet_1.zip › Single-cell sequencing analysis/T cells/P22082602_TopMarkergenedotplot.png]

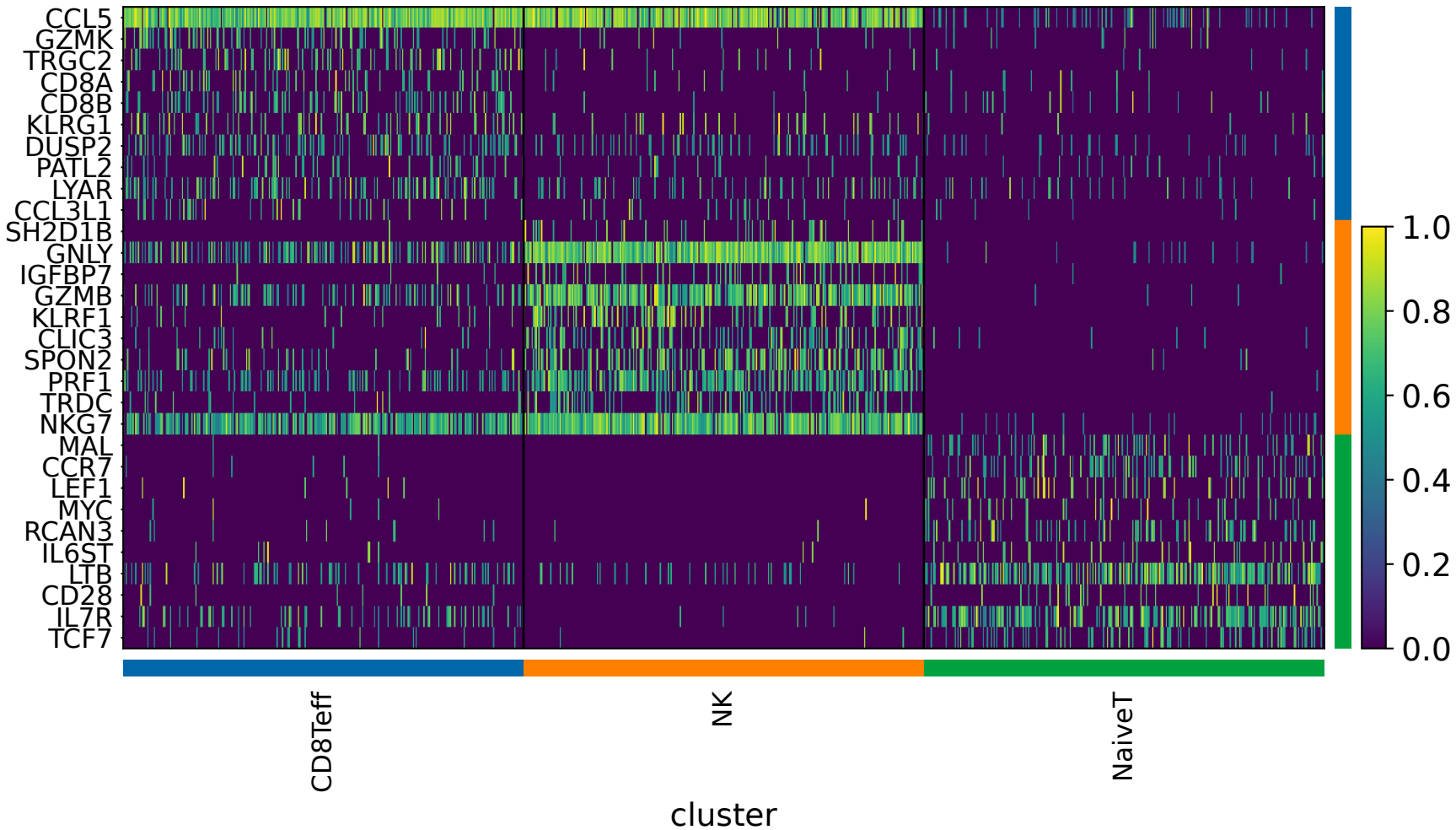

Supplement: Supplementary file 1 [file DataSheet_1.zip › Single-cell sequencing analysis/T cells/P22082602_TopMarkergeneHeatmap.pdf]

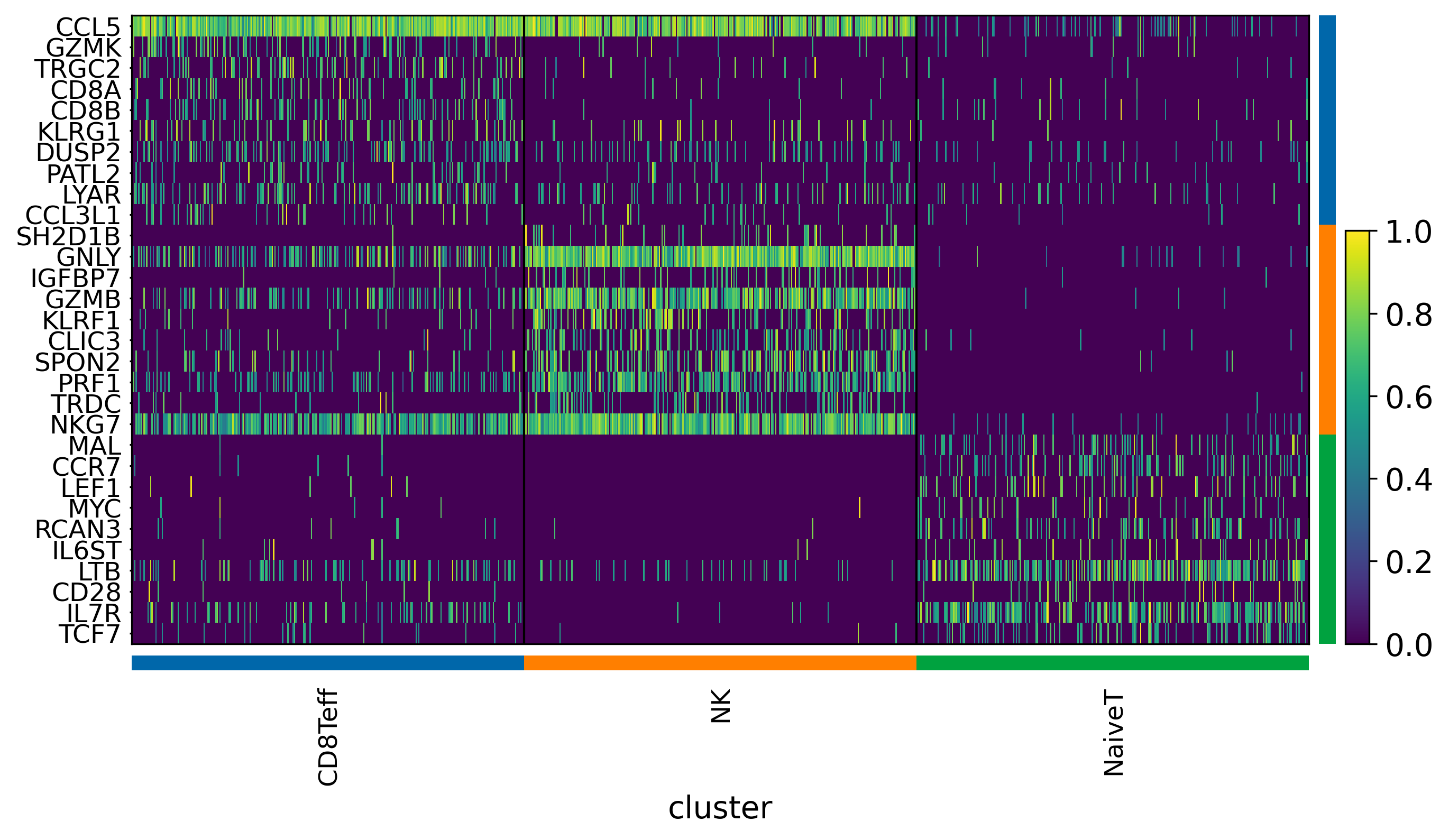

Supplement: Supplementary file 1 [file DataSheet_1.zip › Single-cell sequencing analysis/T cells/P22082602_TopMarkergeneHeatmap.png]

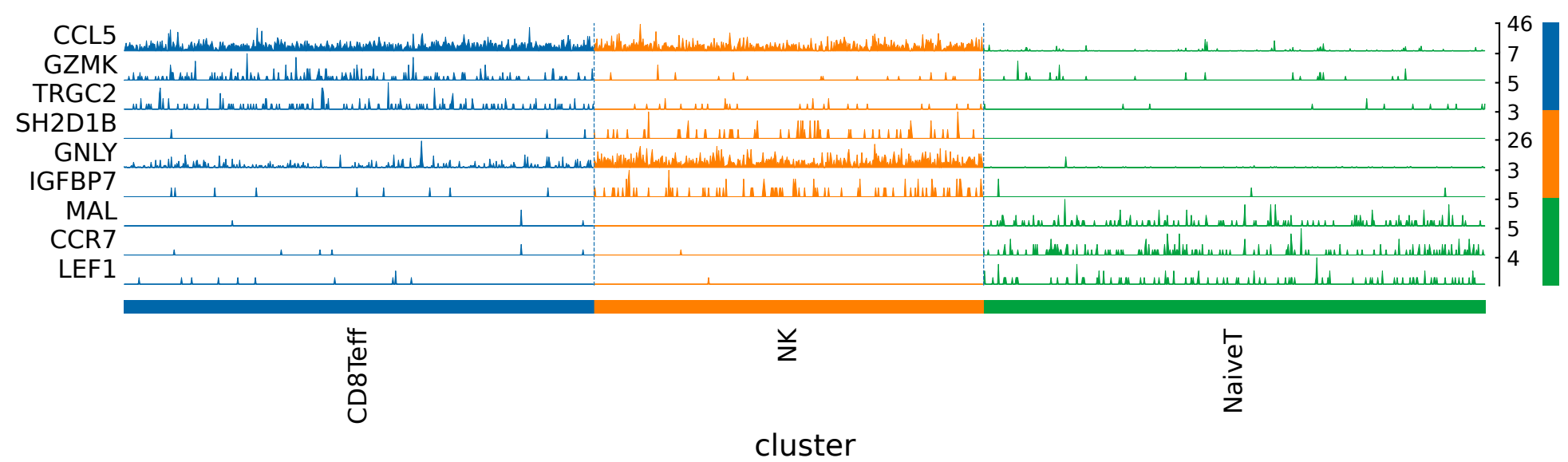

Supplement: Supplementary file 1 [file DataSheet_1.zip › Single-cell sequencing analysis/T cells/P22082602_TopMarkergeneTracksplot.pdf]

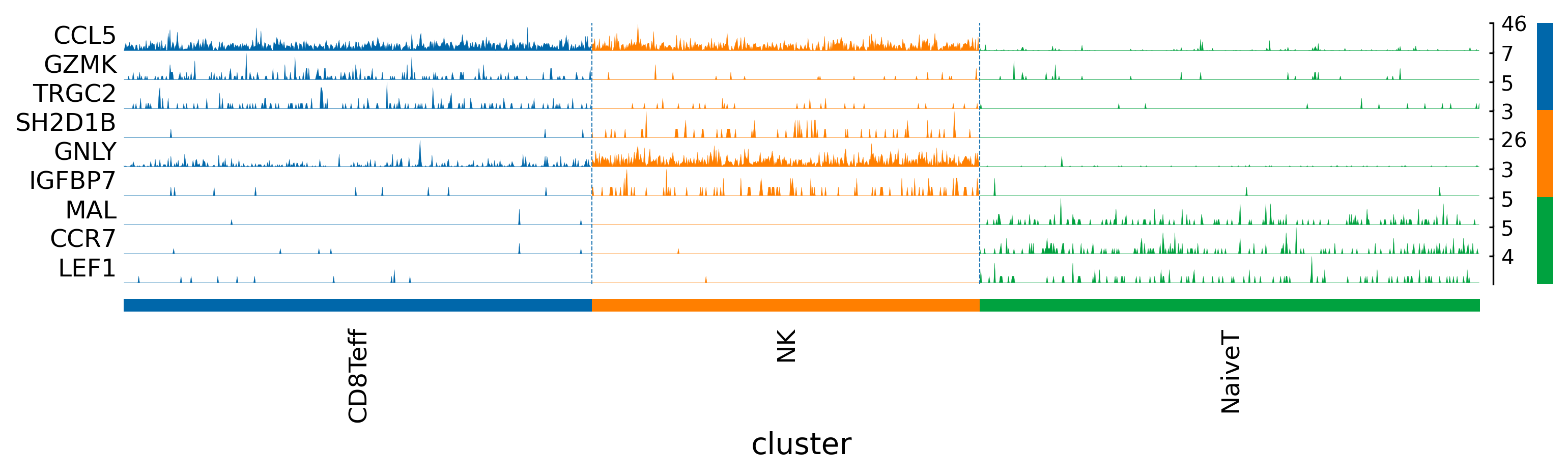

Supplement: Supplementary file 1 [file DataSheet_1.zip › Single-cell sequencing analysis/T cells/P22082602_TopMarkergeneTracksplot.png]

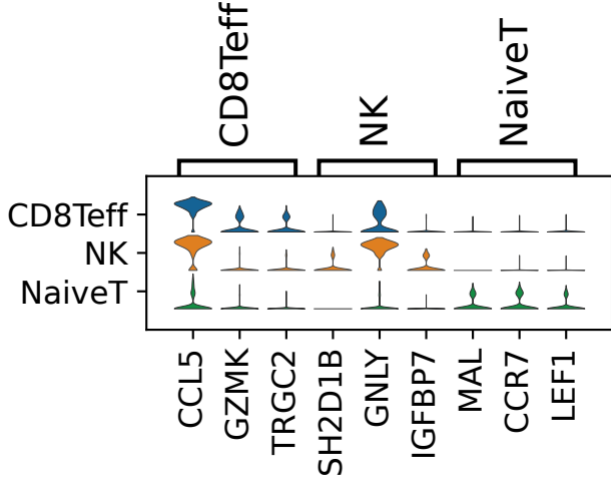

Supplement: Supplementary file 1 [file DataSheet_1.zip › Single-cell sequencing analysis/T cells/P22082602_TopStackedViolin.pdf]
