# Supplementary material for: Porcine anti-human lymphocyte immunoglobulin depletes the lymphocyte population to promote successful kidney transplantation
Source: Front Immunol. 2023 Mar 9;14:1124790. doi: 10.3389/fimmu.2023.1124790 (PMC10033525; doi:10.3389/fimmu.2023.1124790)
Supplement: Supplementary file 2 [file Table_1.docx]

| **Table 1. The number of cells** | | |
| --- | --- | --- |
|  | **0 day** | **7 days** |
| Total cells | 7762 | 7481 |
| T cells | 770 | 283 |
| B cells | 135 | 142 |
| Monocytes | 567 | 503 |
| cDCs | 37 | 5 |
| Neutrophils | 5726 | 6278 |
| NK cells | 316 | 106 |
| *Abbreviations: cDCs, conventional DCs* | | |
