# Supplementary material for: Porcine anti-human lymphocyte immunoglobulin depletes the lymphocyte population to promote successful kidney transplantation
Source: Front Immunol. 2023 Mar 9;14:1124790. doi: 10.3389/fimmu.2023.1124790 (PMC10033525; doi:10.3389/fimmu.2023.1124790)
Supplement: Supplementary file 3 [file Table_2.docx]

**Table 2. The catalog numbers of the antibodies**

| **Catalog number** | **Antibodies** |
| --- | --- |
| FHN045-01-100 | CD45 |
| FHW003-01-100 | CD3 |
| FHC004-01-100 | CD4 |
| FHP056-02-100 | CD56 |
| FHP016-01-100 | CD16 |
| FHA019-01-100 | CD19 |
| FHODR-02-100 | HLA-DR |
| FHF008-01-100 | CD8 |
| FHO127-02-100 | CD127 |
| FHP025-02-100 | CD25 |
| FHF068-01-100 | CD68 |
| FHP011c-01-100 | CD11c |
